# Immunomodulatory Potential of Cannabidiol in Multiple Sclerosis: a Systematic Review

**DOI:** 10.1007/s11481-021-09982-7

**Published:** 2021-01-25

**Authors:** Alessia Furgiuele, Marco Cosentino, Marco Ferrari, Franca Marino

**Affiliations:** Center for Research in Medical Pharmacology and Center for Research in Neuroscience, University of Insubria, Via Ottorino Rossi n. 9, 21100 Varese, VA Italy

**Keywords:** Multiple sclerosis, Experimental autoimmune encephalomyelitis, Cannabidiol, Immunomodulation

## Abstract

Multiple sclerosis (MS) is the most common chronic autoimmune disease of the central nervous system. Efficacy of treatments for MS is associated with risk of adverse effects, and effective and well-tolerated drugs remain a major unmet need. Cannabis (*Cannabis sativa* L., fam. Cannabaceae) and cannabinoids are popular among MS patients to treat spasticity and pain. Cannabinoids are endowed with remarkable immunomodulating properties, and in particular the non-psychotropic cannabinoid cannabidiol (CBD) is increasingly recognized as anti-inflammatory and immunosuppressive, nevertheless with excellent tolerability even at high doses. In this systematic review, we retrieved and critically evaluated available evidence regarding the immune and disease-modifying effects of CBD in experimental autoimmune encephalomyelitis (EAE) and in MS. Evidence in rodent models of EAE strongly supports CBD as effective, while clinical evidence is still limited and usually negative, due to paucity of studies and possibly to the use of suboptimal dosing regimens. Better characterization of targets acted upon by CBD in MS should be obtained in ex vivo*/*in vitro studies in human immune cells, and higher doses should be tested in well-designed clinical trials with clinically relevant efficacy endpoints.

**Supplementary Information:**

The online version contains supplementary material available at 10.1007/s11481-021-09982-7.

**Figure:**
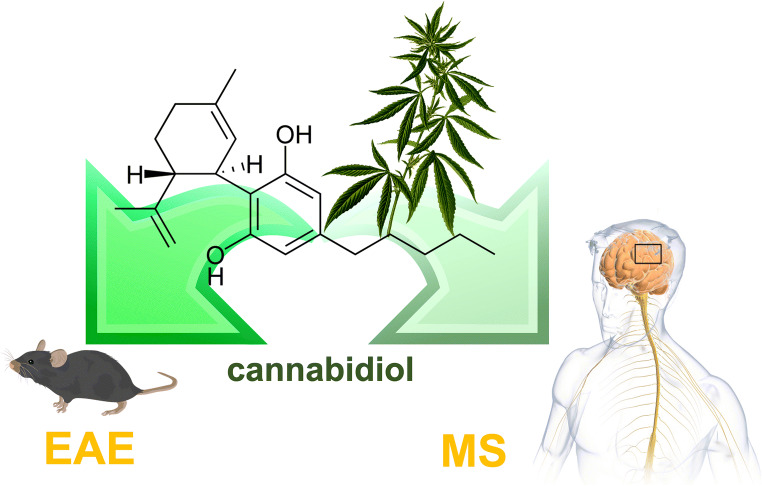
Graphical Abstract

## Introduction

Multiple sclerosis (MS) is the most common chronic autoimmune disease of the central nervous system (CNS), affecting more than two million people worldwide. MS has unknown etiology, is at least as twice as common in women than in men, and usually begins in adults 20–45 years of age, developing through a highly heterogeneous and unpredictable course: neurological deficits are usually reversible in the early phases but over time evolve in progressive neurological deterioration. Based on the clinical course, MS is usually divided in four major forms: (i) relapsing-remitting MS (RRMS), which affects 85% of MS patients, (ii) secondary progressive MS (SPMS), which may develop in some RRMS patients, (iii) primary progressive MS (PPMS), which affects approximately 10% of MS patients, and (iv) progressive-relapsing MS (PRMS), occurring in fewer than 5% of patients (Dobson and Giovannoni 2019; Reich et al. 2018; Oh et al. 2018; Thompson et al. 2018).

MS is characterized by inflammation, demyelination and neurodegeneration, which are regarded as resulting from autoreactive myelin-specific T lymphocytes entering the CNS. T cells undergo reactivation in the CNS by local antigen presenting cells, eventually triggering an inflammatory cascade including release of proinflammatory cytokines such as tumor necrosis factor (TNF)-α, and interferon (IFN)-γ, recruitment of additional inflammatory cells (T cells, monocytes, B cells), persistent activation of macrophages resulting in oligodendrocyte death and further demyelination (Yamout and Alroughani 2018; Hemmer et al. 2002).

MS has no known cure so far, nonetheless several immunomodulatory and immunosuppressive treatments have proven helpful at slowing disease progression and reducing relapse rates, including IFN- β, glatiramer acetate, dimethyl fumarate, the type II topoisomerase inhibitor mitoxantrone, the inhibitor of pyrimidine synthesis teriflunomide, the purine analog cladribine, the sphingosine-1-phosphate (S1P) receptor agonists fingolimod, siponimod, and ozanimod, and several monoclonal antibodies such as natalizumab, alemtuzumab, ocrelizumab. The clinical efficacy and risk-benefit ratio of all these treatments are however still far from optimal, and the more effective medications have a higher risk of serious adverse reactions (Gholamzad et al. 2019; Thompson et al. 2018).

Besides disease-modifying treatments targeting pathogenetic mechanisms, management of MS includes a wide array of pharmacological and non-pharmacological approaches aimed at minimising disease impact while maximising quality of life (Gholamzad et al. 2019; Thompson et al. 2018). Among pharmacological treatments for the symptomatic management of MS, cannabis (*Cannabis sativa* L., fam. Cannabaceae) and its derivatives, such as Δ^9^-tetrahydrocannabinol (Δ^9^-THC) and the non-psychotropic cannabinoid cannabidiol (CBD), are increasingly recognized as effective to treat spasticity and pain (Yadav et al. 2014). In 2010, nabiximols – a formulated cannabis extract containing Δ^9^-THC and CBD in a 1:1 ratio – was licensed in UK for the treatment of spasticity due to MS, and it is currently marketed under the trade name of Sativex® in more than 25 countries outside the USA (https://www.gwpharm.co.uk/healthcare-professionals/sativex). The use of cannabis and cannabinoids is widespread and well accepted among patients with MS. Epidemiological studies show that MS patients increasingly use cannabis preparations for a range of symptoms, including sleep disturbances, pain, anxiety, spasticity and even depression. Across the surveys, current use of cannabis is reported by 20–60% of people with MS, and 50–90% are in favour of legalization, would consider usage if it were legal, and ask for more scientific evidence (Schabas et al. 2019; Brenton et al. 2018; Loraschi et al. 2016; Banwell et al. 2016).

Several lines of evidence indicate that cannabinoids have immunomodulatory and immunosuppressive properties, suggesting these drugs as potential therapeutics in chronic inflammatory diseases (Klein 2005), and cannabinoid receptors have been recently proposed as therapeutic targets for autoimmune diseases including MS (Gonçalves and Dutra 2019). Cannabis use in clinical practice has been historically hampered by the addictive potential of Δ^9^-THC, as well as by its psychoactive effects, such as cognitive impairment, psychosis, dysphoria, and anxiety. CBD however is devoid of any drug abuse liability (Babalonis et al. 2017) and is well tolerated in humans up to 6000 mg/day p.o. (Taylor et al. 2018; Iffland and Grotenhermen 2017; Bergamaschi et al. 2011). CBD has recently received Food and Drug Administration (FDA) and European Medicines Agency (EMA) approval for seizures associated with Lennox-Gastaut syndrome or Dravet syndrome (https://www.epidiolex.com/, Chen et al. 2019). CBD has prominent anti-inflammatory and even immunosuppressive effects (Nichols and Kaplan 2020; Zurier and Burstein 2016; Burstein 2015), and evidence exists that it could be beneficial in chronic inflammatory conditions, such as inflammatory bowel disease (Esposito et al. 2013), rheumatoid arthritis (Lowin et al. 2019), neurodegenerative disorders (Cassano et al. 2020), and even in acute inflammation due to SARS-CoV-2 infection (Costiniuk and Jenabian 2020). Despite the widespread use of CBD for the symptomatic management of MS, the possible relevance of its immunomodulatory properties and its potential as disease-modifying drug in MS patients has so far received little consideration.

In the present review, after a thorough description of the complex pharmacology of CBD, which includes several molecular targets besides cannabinoid receptors, available preclinical and clinical evidence about the immune effects of CBD in MS is presented and discussed, to provide a summary of available knowledge and define a roadmap for the extensive assessment of the immunomodulatory potential of CBD in MS patients.

## Pharmacology of CBD

### Pharmacodynamics

**Fig. 1:**
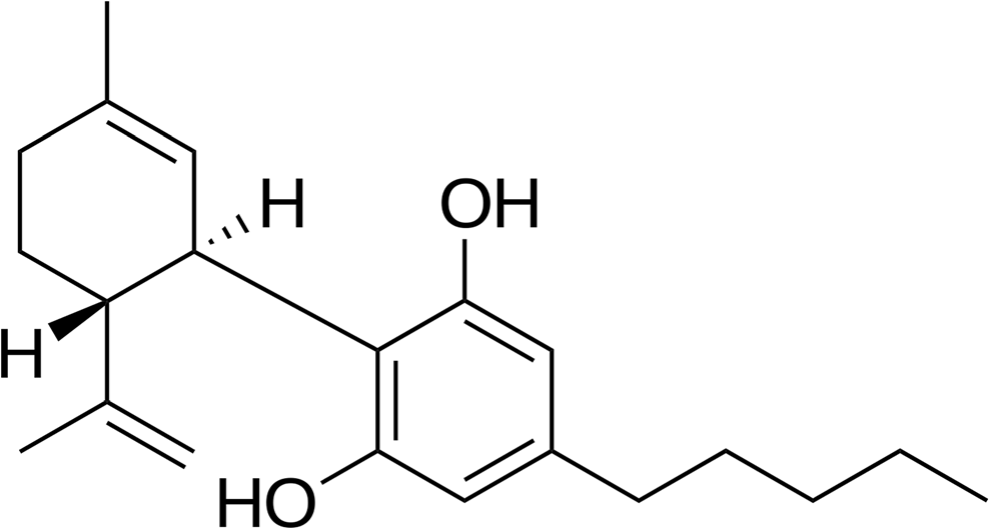
Chemical structure of CBD

CBD is a natural cannabinoid isolated in 1940 from cannabis plants (Mechoulam et al. 1970) (Fig. 1). It is the major non-psychoactive cannabinoid and occurs naturally in appreciable amounts in the plant leaves and flowers, accounting for up to 40% of the plant’s extracts obtained from newly developed varieties poor in Δ^9^-THC (Andre et al. 2016).

CBD has a quite complex receptor pharmacology (Table 1). CBD is indeed a weak activator of cannabinoid receptors type 1 (CB1) and type 2 (CB2). Actually, CBD may also act as a negative allosteric modulator of the CB1 receptor, and as an inverse agonist of the CB2 receptor (Pertwee 2008). CBD however also acts on several mammalian transient receptor potential (TRP) channels, including TRPV (“V” for vanilloid), TRPA (“A” for ankyrin), and TRPM (“M” for melastatin) (Muller et al. 2019). CBD acts as an agonist on TRPV1, resulting in capsaicin-like analgesia (Iannotti et al. 2014). CBD may also bind and activate TRPV2, TRPV3, and TRPA1, while being an antagonist at TRPM8 (Muller et al. 2019). CBD is an agonist of the peroxisome proliferator-activated receptor (PPAR) γ, which a ligand-inducible transcription factor belonging to the superfamily of nuclear receptors (O’Sullivan et al. 2009). CBD also binds some G protein-coupled orphan receptors (GPR). In particular, it has been reported to act as an antagonist at GPR55, and as an inverse agonist at GPR3, GPR6 and GPR12 (Atalay et al. 2019). Finally, CBD may be an agonist at serotonin (5-hydroxytryptamine, 5-HT) receptors 1a (Russo et al. 2005), and at the adenosine A_2A_ receptors (Ribeiro et al. 2012), and possibly an allosteric modulator at μ and δ opioid receptors (Kathmann et al. 2006).

**Table 1:** CBD pharmacology

Target	Action	Reference
CB1	Weak agonist negative Allosteric modulator	Pertwee (2008)
CB2	Weak agonist inverse agonist	Pertwee (2008)
TRPV1, TRPV2, TRPV3, TRPA1	Agonist	Muller et al. (2019)
TRPM8	Antagonist	Muller et al. (2019)
PPARγ	Agonist	O’Sullivan et al. (2009)
GPR55	Antagonist	Atalay et al. (2019)
GPR3, GPR6, GPR12	Inverse agonist	Atalay et al. (2019)
5-HT1a	Agonist	Russo et al. (2005)
A_2A_	Agonist	Ribeiro et al. (2012)
μ and δ opioid receptors	Allosteric modulator	Kathmann et al. (2006)

Abbreviations: *CB* cannabinoid receptors, *TRP* transient receptor potential channels, “*V*” for vanilloid, “*A*” for ankyrin, and “*M*” for melastatin, *PPARγ* peroxisome proliferator-activated receptor γ, *GPR* G protein-coupled orphan receptors, *5-HT1a* 5-hydroxytryptamine receptor 1a, *A*_*2A*_ adenosine receptor 2A

Remarkably, besides its direct effects on multiple receptor targets, CBD has prominent direct and indirect antioxidant effects (Atalay et al. 2019) as well as the ability to block the enzyme fatty acid amide hydrolase, resulting in an inhibited degradation and therefore increased levels of anandamide. a fatty acid neurotransmitter acting as agonist on CB1 and CB2, as well as on several other receptor targets, including among others TRPV1, TRPM8, and GPR55 (Lim et al. 2017).

### Pharmacokinetics

CBD pharmacokinetics (PK) has been recently systematically reviewed by Millar et al. (2018), who retrieved, summarized and discussed all articles reporting PK data of CBD in humans. The authors conclude that, despite the widespread clinical use of CBD, information about its PK is limited and inconsistent, and highlight the need for thorough studies aimed at the better understanding of key PK parameters such as bioavailability and half-life.

### Pharmacogenetics

CBD acts on many molecular targets (Table 1), most of them with evidence of genetic variability linked to some functional consequences. For instance, CB1 and CB2 have been extensively studied for involvement in cannabis dependence (Hryhorowicz et al. 2018), mutations in TRPV channels are known from genetic pain research and may modulate the effects of experimental analgesics targeting TRPV1 or TRPV3 (Lötsch and Geisslinger 2011), PPARγ genetic variants are a promising target for precision medicine in Type 2 diabetes mellitus (Khatami et al. 2019). No studies exist so far investigating the role of such genetic variants in the effects of CBD, nevertheless, pharmacogenomic clinical trials of cannabinoids are currently ongoing, such as those examining the effects of the catechol-O-methyl-transferase (COMT) gene on the effects of CBD (NCT02116010 n.d.; NCT02492074 n.d.).

Compared to the lack of pharmacogenetic studies about CBD targets, more evidence exists concerning CBD PK. CBD absorption and distribution are influenced by P-glycoprotein (P-gp), an efflux protein encoded by *ABCB1* gene, also known as multidrug resistance gene (MDR1), located in chromosome7q21 and composed of 28 exons (Hoffmeyer et al. 2000). SNPs in the *ABCB1* gene such as rs2032582 (c.2677G T > A), rs1045642 (c.3435C > T), and rs1128503 (c.1236 C > T) are known to modify P-gp expression and activity and in turn PK of many drugs. No information is however available about their potential relevance for CBD PK (Rui-Jian et al. 2017). CBD is metabolized by cytochrome P450 (CYP450) superfamily enzymes, and in particular by CYP3A4 and CYP2C9 (Stout et al., 2014), which are encoded by *CYP2C9* and *CYP3A4* genes. To date, 60 polymorphic alleles of the CYP2C9 gene have been described, the most frequent being CYP2C9*2 (c.430 C > T), and CYP2C9*3 (c.1075 A > C) which lead to decreased enzyme activity and poor metabolizer phenotype (Jarrar and Lee 2014). In the case of CYP3A4 gene, 26 polymorphic alleles are known, and CYP3A4*2, CYP3A4*11, CYP3A4*12, CYP3A4*17 are the most common, resulting in reduced enzyme activity (Werk and Cascorbi 2014). Unfortunately, no information is so far available on the effect of these SNPs on CBD PK in humans. UDP-glucuronosyltransferase (UGT) enzyme family is also involved in CBD biotransformation (Stout and Cimino 2014), in particular UGT1A9, UGT2B7, and UGT2B17. Important SNPs in the *UGT1A9* gene such as UGT1A9 *3, *4, and UGT1A9 *5 lead to the reduction or suppression of enzymatic activity (Olson et al. 2009). However, CBD glucuronidation has a minor role in overall elimination of the drug (Mazur et al. 2009), therefore genetic variants in UGT enzymes are unlikely to affect CBD PK to a major extent.

### Aim

In the present review, we systematically retrieved and critically evaluated available evidence regarding the immune effects and the disease-modifying activity of CBD in MS and in experimental autoimmune encephalomyelitis (EAE), its preclinical animal model, to provide a state-of-the-art compendium of the immunomodulatory potential of CBD in MS.

### Search Strategy

**Fig. 2:**
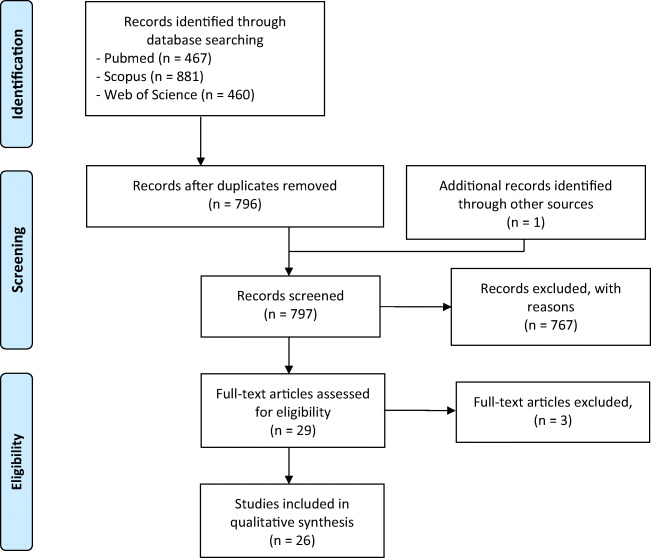
Flow diagram of literature search

This systematic review was conducted in accordance with the PRISMA statement (Moher et al. 2009). Search algorithm was obtained by combining terms related to “cannabidiol” with those related to “multiple sclerosis” or “experimental allergic encephalomyelitis” as shown in Table 2, and search was thereafter performed in PubMed, Scopus and Web of Science databases (Fig. 2). References identified through this process were subsequently scanned for selection criteria. Inclusion criteria included studies of the peripheral and central immune effects of CBD, either pure or in botanical extracts, alone or together with other drugs. Excluded topics included review articles, duplicates, and studies of synthetic analogues, or metabolites of CBD. Thereafter, reference lists of the included articles were screened for additional reports. Neither language nor year restrictions was applied and all reports issued in the period up to July 29, 2020 were included.

**Table 2:** Search algorithm for database screening

Cannabidiol	Multiple sclerosis
Cannabidiol	Multiple sclerosis
Cannabidiol-3-monomethyl ether	Multiple sclerosis, relapsing-remitting
5-(1,1-dimethylheptyl)cannabidiol	Multiple sclerosis, chronic progressive
Nabiximols	Experimental autoimmune Encephalomyelitis
6″-azidohex-2″-yne-cannabidiol	Experimental allergic encephalomyelitis
Cannabidiol (abn-cbd, (−)-4-(3–3,4-trans-p-menthadien-(1,8)-yl)olivetol)	
4-(3–3,4-p-menthadien-(1,8)-yl)olivetol	
Desoxycannabidiol	
Cannabidiol hydroxyquinone	
Cannabidiol dimethyl ether	
HUF-101	

https://www.ncbi.nlm.nih.gov/mesh/?term=cannabidiol

https://www.ncbi.nlm.nih.gov/mesh/?term=multiple+sclerosis

https://www.ncbi.nlm.nih.gov/mesh/?term=experimental+allergic+encephalomyelitis

## Results

Our literature search led to a total of 1808 reports. After screening for relevant titles and abstracts, 29 papers were assessed for full-text eligibility, and 26 studies were finally included in the review (Fig. 2). All the records screened are listed as supplementary material (Supplementary Table 1).

### Preclinical Studies

We found a total of 20 in vivo and ex vivo*/*in vitro studies of CBD in preclinical models of MS (Table 3). Most animal studies were performed in (MOG_35–55_)-induced EAE in C57BL/6 J mice. Individual studies however were also performed in EAE induced in mice by means of MSCH (Buccellato et al. 2011; Duchi et al. 2013), PLP_139–151_ (Gallily and Yekhtin 2019), TMEV (Mecha et al. 2013), and cuprizone (Sajjadian et al. 2017). One study made use of C57BL/6 J mice with adoptively transferred EAE (González-García et al. 2017), and another one was performed in Lewis rats with protein gp (69–88)-induced EAE (Zhou et al. 2019).

**Table 3:** Effect of CBD in preclinical models of MS

Experimental model	Treatment	Main findings	Mechanisms/biological target	Ref
In vivo				
(MOG_35–55_)-induced EAE in C57BL/6 J mice	CBD (75 mg/kg/day by oral gavage) 24 h after EAE induction and subsequently for 5 days	Reduction of clinical score at day 18 in severe but not in mild EAE	No change in percentage of Treg isolated from the lymph nodes and spleen, or of MDSC from spleen	Nichols et al. (2020)
		Reduction of neuroinflammation and T cell infiltration in white matter tracts of brain and spinal cord	In ex vivo splenocytes restimulated with MOG_35–55_ for 48 h, CBD decreased percentage of IFN-γ producing CD8+ T cells but did not affect IL-17-producing CD8+ T cells	
			In ex vivo splenocytes and lymphocytes from lymph nodes restimulated with MOG_35–55_ for 48 h, CBD did not affect IFN-γ and IL-17A production on day 3 and 10, but increased IFN-γ production on day 18	
(MOG_35–55_)-induced EAE in C57BL/6 J mice	CBD (10 mg/kg/day i.p.) or Δ9-THC + CBD (10 mg/kg/day i.p.) from day 10 after EAE induction until day 15/27	Δ9-THC + CBD (but not CBD alone) reduced clinical symptoms, brain infiltration of MNCs, CD3+ T cells and CD3 + CD4+ T cells, and demyelination	Reduced IL-17A and IFN-γ production in iLN cell supernatants	Al-Ghezi et al. (2019b)
			Changes in the expression in brain CD4 + T cells of several miRNAs involved in Th polarization and cell cycle arrest/apoptosis	
			Upregulation of genes for FoxP3, STAT5, IL-10 and IL-4 and downregulation of genes for Tbet-1, IFN-γ, STAT3 and IL-17A in brain MNCs	
			Increased production by brain MNCs of IL-10 and TGF-β and reduced production IL-17A, IFN-γ, TNF-α, IL-6, and IL-1b	
			In brain MNCs increased apoptosis and decreased cell frequency in G0/G1 phase and increased frequency in G2/M phase	
			Possible involvement of CB1 and CB2 receptors, based on results in CB1−/− CB2−/− animals	
(MOG_35–55_)-induced EAE in C57BL/6 J mice	Δ9-THC + CBD (1:1 ratio) (10 + 10 mg/kg/day i.p.) from day 10 after EAE induction until day 15/18	Decreased EAE severity, including reduced cell infiltration and tissue damage in the brain	Increased spleen MDSC and reduced pro-inflammatory cytokines (IL-17A and IFN-γ) and increased anti-inflammatory cytokines (TGF-β and IL-10) in serum and spleen cells supernatants	Al-Ghezi et al. (2019a)
			Reduced abundance of *Akkermansia muciniphila* (A. muc) and decreased LPS biosynthesis in the gut	
			Increased levels of SCFAs such as butyric, isovaleric, and valeric acids	
(MOG_35–55_)-induced EAE in C57BL/6 J mice	CBD (20 mg/kg/day i.p.) from day 9 to 25 post-EAE induction	Delayed onset and attenuated clinical signs of EAE	Reduced MNCs in the spinal cord and reduced CD3 + CD4+ and CD3 + CD8+ T cells in the CNS	Elliott et al. (2018)
			Reduced IFN-γ and IL-17 in serum	
			Reduced gene expression of T-bet and ROR-γ and increased gene expression of IL-10 in splenic CD4 + T cells	
			In ex vivo splenocytes restimulated with MOG35–55, reduced production of IFN-γ and IL-17, and increased production of IL-10	
			Induction of highly immunosuppressive CD11b + Gr-1+ MDSCs which reduced clinical scores of EAE and cellular infiltration in the CNS	
			MDSC depletion reversed CBD effects	
(MOG_35–55_)-induced EAE in C57BL/6 J mice	CBD (10 mg/kg/day i.p.) from day 14 until day 28 after EAE induction	Improved EAE clinical and histological score with reduced infiltration of inflammatory cells in the white matter of spinal cord	Suppressed IFN-γ and IL-17 staining and increased BDNF and PPARγ staining in spinal cord sections	Giacoppo et al. (2017)
			Upregulation of the PI3K/Akt/mTOR pathway and reduced expression of JNK and p-p38 in the MAPK pathway in the spinal cord	
(MOG_35–55_)-induced EAE in C57BL/6 J mice	Δ9-THC-BDS + CBD-BDS (30.8 mg/kg/day i.p., equivalent to about 10 mg/kg/day of pure Δ9-THC + 10 mg/kg/day of pure CBD) or CBD-BDS alone (20 mg/kg/day i.p., equivalent to about 13 mg/kg/day of pure CBD) at days 10–31 post MOG-inoculation	All treatments delayed symptoms onset but only Δ9-THC-BDS + CBD-BDS also improved disease progression	Activation of CB1 receptors, but not of PPAR-γ	Moreno-Martet et al. (2015)
(MOG_35–55_)-induced EAE in C57BL/6 J mice	CBD cream 1% applied on lower limbs every 24 h up to 28 days after disease onset	EAE clinical score improvement and avoidance of EAE-associated body weight loss	Reduced FoxP3 staining in spinal cord and reduced CD4 and CD8α staining in spleen	Giacoppo et al. (2015)
		Reduced demyelination and axonal loss and complete resolution of inflammatory cells infiltration	Reduced TNF-α, IL-6, TGF-β and INF-γ and increased IL-10 in spinal cord	
		Increased response to mechanical stimuli	Reduced production of nitrotyrosine, iNOS and PARP, and reduced cleaved-caspase 3 expression in spinal cord	
(MOG_35–55_)-induced EAE in C57BL/6 J mice	CBD (5 mg/kg/day i.p.) or CBD + PEA (5 mg/kg/day each i.p.) for 3 days after disease onset.	All treatments attenuated cell infiltration and microglia activation in the spinal cord, but only CBD alone ameliorated neurological signs and disease progression, and decreased demyelination severity in the spinal cord	All treatments decreased TNF-α, IFN-γ and IL-17 mRNA levels in spinal cord	Rahimi et al. (2015)
(MOG_35–55_)-induced EAE in C57BL/6 J mice	CBD (5 mg/kg/day i.p.) on days 19, 20 and 21 after EAE induction	Ameliorated clinical signs and disease progression, decreased axonal damage, inflammation and CD3+ T cells infiltration in the spinal cord	Not assessed	Kozela et al. (2011)
		Decreased MOG-induced microglia/macrophage activation		
Cuprizone-induced demyelination in C57Bl/6 mice	CBD (5 mg/kg/day i.p.) for 5 weeks	Restoration of cuprizone-induced myelin loss and attenuated microglial accumulation in the corpus callosum midline	Reversal of cuprizone-induced reduction of GSH, CAT, and SOD and increase of lipid peroxidation in the corpus callosum	Sajjadian et al. (2017)
MSCH-induced CREAE in Biozzi AB/H mice	Δ9-THC-rich chemovar (ratio of Δ9-THC: CBD 9:1) (50 mg/kg i.p.) or CBD-rich chemovar (ratio of Δ9-THC: CBD 1:12) (50 mg/kg i.p.) or both (25 + 25 mg/kg i.p.) as single injection 32 days after CREAE induction or one injection/day from day 68 after CREAE induction for 7 days	Δ9-THC-rich chemovar alone, for 7 days but not as single injection, reduced the overall neurological deficits	Not assessed	Buccellato et al. (2011)
		CBD-rich chemovar alone, either as single injection or for 7 days, decreased neurological deficit during the relapse phase		
		Δ9-THC- + CBD-rich chemovar had no effect, except an unexpected increased in neurological deficits in some animals		
MSCH-induced EAE in C57Bl/6 J mice	CBD (7 mg/kg s.c.) alone or + GA (6.7 mg/kg s.c. or intranasally) on the first day of disease onset	CBD s.c. or intranasally reduced the clinical signs of disease	CBD and CBD + GA in intranasally reduced IL-6 and TNF-α expression in cerebellum	Duchi et al. (2013)
		CBD + GA intranasally had more effect than CBD alone or CBD + GA s.c.	CBD + GA intranasally induced proliferation of newly generated neurons in SVZ and SGZ of the hippocampus	
PLP_139–151_-induced relapsing–remitting EAE in SJL/J mice.	CBD (5 mg/kg i.p.) or CBD-rich (18%)/Δ9-THC-poor (1%) *Cannabis indica* extract (50 mg/kg i.p.) 5 days/week for 60 days, in comparison to glatiramer acetate (50 mg/kg s.c.)	CBD and *C. indica* extract inhibited EAE clinical symptoms with a rapid onset and to the same extent as glatiramer	Not assessed	Gallily and Yekhtin (2019)
TMEV-IDD SJL/J mice	CBD (5 mg/kg/day i.p.) from day 1 to 7 or 10 post-infection	Decreased leukocyte infiltration and attenuated microglia activation in brain and spinal cord	Blockade of TMEV-induced release of sVCAM-1 in endothelial cells, and consequently reduced leukocyte adhesion to endothelial cells	Mecha et al. (2013)
			Reduced expression of CCL2, CCL5 and CCR2 mRNA in prefrontal cortex	
Adoptively transferred EAE (at-EAE) in C57BL/6 J mice	CBD (5–10 mg/kg/3 times per week or 50 mg/kg/day i.p.) from day 0 until day 23 after at-EAE induction	Dose-dependent reduction of clinical signs and tissue lesions in the *subiculum*	Reduction of at-EAE-induced infiltration of microglia	González-García et al. (2017)
			No effect on at-EAE-induced decrease of CB1 receptors, but reduction of at-EAE-induced increase of CB2 and GPR55 receptors	
			Highest CBD doses also reduced T cells and macrophages in the lumbar spinal cord as well as axonal damage and demyelination	
Protein gp (69–88)-induced EAE in Lewis rats	CanniMed Oil® Huile (Δ9-THC:CBD 10:10 and 1:20) (215 mg/kg/day by oral gavage) from day 6 to 18 post-EAE induction	Both formulations improved neurological disability score (NDS)	Reduced TNF-α expression and enhanced BDNF production in spinal cord	Zhou et al. (2019)
Ex vivo*/*In vitro				
MOG_35–55_-specific T cell line from lymph node cells of C57BL/6 mice	In APC/T cells cocultures, CBD (5 μM) + MOG_35–55_ (5 μg/ml) for 8 h (gene profiling) or 24 h (ELISA)	Decreased Xcl1, Il12a, Dusp6 and increased Btla, Lag3, and Irf4 mRNA levels	Downregulation of proinflammatory transcription factors and pathways favouring Th17 differentiation and function	Kozela et al. (2016a)
		Decreased IL-1β and IL-3 secretion	Upregulation of gene expression patterns promoting T cell exhaustion/tolerance and IFN-dependent anti-proliferative program	
			Downregulation of APC/T cells interactions and induction of antioxidant mechanisms	
MOG_35–55_-specific T cell line from lymph node cells of C57BL/6 mice	In APC/T cells cocultures, CBD (5 μM) + MOG_35–55_ (5 μg/ml) for 8 h	Increased CD4 + CD25 − CD69+ and CD4 + CD25 − LAG3+ T cells, and no effect on CD4 + CD25+ T cells	Decreased STAT3 and Akt phosphorylation and increased STAT5 phosphorylation	Kozela et al. (2015)
			Increased EGR2, LAG3, STAT5 and IL-10 mRNA levels	
			Increased levels of anergy promoting genes (Lag3, Icos, Nfatc1, Ndrg1, Cdkn1a, Casp4 and Fas)	
			Decrease CD19^high^MHCII^high^, CD19 + CD25+ and CD19^high^CD69^high^ B cells	
MOG_35–55_-specific T cell line from lymph node cells of C57BL/6 mice	In APC/T cells cocultures, CBD (0.1–1-5 μM) + MOG_35–55_ (5 μg/ml) for 8 h (gene profiling) or 24 h (ELISA and FACS analysis)	Dose-dependent decrease of IL-6 and IL-17, but not of TNF-α and IFN-γ, gene expression and secretion	No involvement of CB1, CB2, 5-HT1A, TRPV1 or PPARγ in the effects of CBD on IL-17 secretion	Kozela et al. (2013)
		Increased death of CD4+ T cells and CD19+ B cells, but not of CD11b + monocytes		
MOG_35–55_-specific T cell line from lymph node cells of C57BL/6 mice	In APC/T cells cocultures, CBD (1, 5, 10 μM) + MOG_35–55_ peptide (1 or 2.5 μg/ml) for 72 h	Inhibition of T cell proliferation	No involvement of either CB1 or CB2 receptors	Kozela et al. (2011)
CD4+ T cells from spleen of (MOG_35–55_)-induced EAE in C57BL/6 J mice	CBD (10 mg/kg/day i.p.) for 7 days after EAE induction	Reversal of EAE-induced proinflammatory phenotype of CD4+ T cells	Modulation of histone methylation (H3K4me3 and H3K27me3) and non-coding RNA (miRNA and lncRNA)	Yang et al. (2019)
	Isolation of CD4+ T cells and 48 h treatment with 30 μg/ml MOG without/with CBD 10 μM			
Encephalitogenic spleen cells (MOG_35–55_ + IL-12) from C57BL/6 J mice with (MOG35–55)-induced EAE	CBD (5–10 μM) for 24 or 48 h	Inhibition of MOG_35–55_/IL-12-induced IL-6 secretion and increased apoptosis	Increased ROS levels	González-García et al. (2017)
			No involvement of GPR55, CB1, or CB2 receptors	
Astrocytes from TMEV-IDD SJL/J mice	CBD (1–5 μM) and IL-1β (10 ng/ml) + TNF-α (10 ng/ml) for 6 h	Reduced CCL2 secretion	A2A receptors involved	Mecha et al. (2013)

Abbreviations: *AB/H* antibody high, *APC* antigen-presenting cell, *BDNF* brain-derived neurotrophic factor, *Btla* B- and T lymphocyte attenuator, *Casp4* caspase 4, *CAT* catalase, *CB1* cannabinoid receptor type 1, *CB1−/−CB2−/−* CB1 and CB2 double-knockout, *CB2* cannabinoid receptor type 2, *CBD-BDS* CBD- botanical drug substance, *CBD* cannabidiol, *MDSCs* myeloid-derived suppressor cells, *CCL2* C-C Motif Chemokine Ligand, *CCL5* C-C Motif Chemokine Ligand 5, *CCR2* C-C Motif Chemokine Receptor 2, *Cdkn1a* cyclin dependent kinase inhibitor 1a, *CNS* central nervous system, *CREAE* chronic relapsing experimental autoimmune encephalomyelitis, *Dusp6* dual specificity phosphatase 6, *EAE* experimental autoimmune encephalomyelitis, *EGR2* early growth response protein 2, *Foxp3* forkhead box P3, *G0* gap 0, *G1* gap 1, *G2* gap 2, *GA* glatiramer acetate, *GPR55* G protein-coupled receptor 55, *GSH* glutathione, *i.p*. intraperitoneal, *Icos* inducible T cell costimulatory, *IFN-γ* interferon-gamma, *IL* interleukin, *IL12a* Interleukin-12 subunit alpha, *iLN* inguinal lymph nodes, *iNOS* inducible nitric oxide synthase, *Irf4* Interferon regulatory factor 4, *JNK* c-Jun N-terminal kinase, *Lag3* lymphocyte-activation gene 3, *M* mitosis, *MAPK* mitogen-activated protein kinase, *miRNAs* microRNAs, *MNCs* mononuclear cells, *MOG* myelin oligodendrocyte glycoprotein, *MS* multiple sclerosis, *MSCH* mouse spinal cord homogenate, *mTOR* mammalian target of rapamycin, *Ndrg1* N-Myc downstream regulated 1, *NDS* nasal delivery system, *Nfatc1* nuclear factor of activated T cells, *p-p38* phosphorylated p38, *PARP* poly (ADP-ribose) polymerase, *PEA* palmitoylethanolamide, *PI3K* phosphoinositide 3-kinase, *PLP* proteolipid protein, *PPAR-γ* peroxisome proliferator-activated receptor gamma, *RORγ* RAR-related orphan receptor gamma, *s.c*: subcutaneous, *SCFAs* shorth chain fatty acids, *SGZ* subgranular zone, *SOD* Cu/Zn superoxide dismutase, *STAT3* signal transducer and activator of transcription 3, *STAT5* signal transducer and activator of transcription 5, *sVCAM-1* soluble vascular cell adhesion molecule-1, *SVZ* subventricular zone, *Tbet* T-box expressed in T cells, *TGF-β* transforming growth factor beta, *Th* T helper, *TMEV-IDD* Theiler’s murine encephalomyelitis virus-induced demyelinating disease, *XCl2* Chemokine (C motif) ligand 2, *Δ9-THC BDS* Δ9-THC- botanical drug substance, *Δ9-THC* delta-9-tetrahydrocannabinol

CBD was given i.p. in 12 out of 15 studies, most often at the dose of 5 mg/kg/day (6 studies), however also up to 20 mg/kg/day (Elliott et al. 2018), with highly variable schedules, administration beginning from immediately up to even 32–68 days after EAE induction (Buccellato et al. 2011), and lasting from 3 up to 60 days (Gallily and Yekhtin 2019). In some studies, CBD was given by oral gavage (Nichols et al. 2020; Zhou et al. 2019), s.c. or intranasally (Duchi et al. 2013), or even as cream 1% applied on lower limbs (Giacoppo et al. 2015). In most cases, CBD was given as pure substance (12 studies), however in some cases it was administered as cannabis extract, together with Δ9-THC in variable proportions (Al-Ghezi et al. 2019a, b; Moreno-Martet et al. 2015; Buccellato et al. 2011; Gallily and Yekhtin 2019; Zhou et al. 2019).

Despite such heterogeneity, treatment with CBD was consistently effective usually resulting in reduced severity of EAE, including delayed onset of symptoms, attenuation of clinical signs and reduced disease progression. Many studies reported also improved CNS histology, with reduced neuroinflammation, microglia activation and peripheral monocyte and lymphocyte infiltration, as well as decreased demyelination.

Experimental evidence about biological mechanisms contributing to CBD-induced beneficial effects in EAE consistently pointed to reduction of proinflammatory cytokines such as IL-17A, IFN-γ, TNF-α, IL-6, and IL-1b, and increase of anti-inflammatory cytokines such as IL-4, IL-10 and TGF-β (Nichols et al. 2020; Al-Ghezi et al. 2019a, b; Elliott et al. 2018; Giacoppo et al. 2017; Giacoppo et al. 2015; Rahimi et al. 2015; Duchi et al. 2013; Zhou et al. 2019), as well as to induction of immunosuppressive MDSC (Al-Ghezi et al. 2019a; Elliott et al. 2018). Very few studies addressed the issue of target receptors involved in the effects of CBD (Moreno-Martet et al. 2015; Al-Ghezi et al. 2019b).

One study (Gallily and Yekhtin 2019) compared CBD to the anti-MS drug glatiramer showing that they were effective to the same extent in reducing EAE.

Preclinical investigation of CBD in EAE also included seven studies performed in ex vivo*/*in vitro models of encephalitogenic lymphocytes (Table 3), all based on T cells from lymph nodes or spleen of mice with (MOG_35–55_)-induced EAE, except for one which used astrocytes from TMEV-IDD SJL/J mice (Mecha et al. 2013). CBD was always used at concentrations ranging from 0,1 to 10 μM, usually resulting in decreased proliferation and increased apoptosis of cells, as well as in inhibition of proinflammatory and activation of anti-inflammatory pathways. Only few studies investigated the molecular targets mediating CBD effects. Kozela et al. excluded the contribution of either CB1, CB2, 5-HT1A, TRPV1 or PPARγ in CBD-dependent reduction of IL-17 secretion from T cells (Kozela et al. 2013), or of CB1 or CB2 in CBD-dependent inhibition of T cell proliferation (Kozela et al. 2011). No involvement of GPR55, CB1, or CB2 receptors was reported also by González-García et al. (2017), who studied CBD-induced inhibition of MOG_35–55_/IL-12-induced IL-6 secretion and increased apoptosis in mouse encephalitogenic spleen cells, while Mecha et al. (2013) suggested a contribution by A2A receptors in CBD-induced reduced of CCL2 secretion from mouse astrocytes.

### Clinical Studies

Our search provided a total of six studies performed in MS patients and/or on immune cells obtained from patients (Table 4).Of these, four studies examined the peripheral immune profile of patients treated with various cannabinoid preparations, one used CBD in ex vivo cultures of PBMC from patients and one did both.

**Table 4:** Studies in MS patients

Type of study	Gender (m/f)	Age (years) mean ± SD (range)	Disease duration (years) mean ± SD	EDSS score mean (min-max)	Treatment	Main findings	Ref
Clinical							
19 MS patients (1 RRMS, 16 SPMS, 2 PPMS) with spasticity responding to 4-weeks nabiximols	8/11	52.0 ± 7.5	21.5 ± 9.9	7.0 (5–8.5)	Nabiximols (on average 7 puffs/each)	In whole blood, upregulation of genes belonging to the ribosome pathway and downregulation of genes related to immune system, cell motility/migration and nervous system	Sorosina et al. (2018)
						Key differentially expressed genes included *NFKB1, RPS3, FYN, MAPK14, TP53*	
30 SPMS patients (7 treated with IFN-β1b; 12 previously treated with IFN-β1; 11 never treated with IFN-β1b)	10/20	54.2 ± 11.7	15.4 ± 8.5	6.4	Nabiximols titration during 4 weeks, with dose increase according to a fixed scheme	No difference in the CB1 or CB2 mRNA levels in peripheral blood leukocytes, before treatment and after one and three months of treatment in any groups, except for patients currently on IFN-β1b that showed decrease CB2 mRNA levels after one and three months of nabiximols treatment	Santoro et al. (2017)
						No effect on methylation of CNR1 or CNR2 promoter regions	
20 MS patients with chronic refractory neuropathic pain (no information on MS type)	7/13	(21–51)	No information provided	No information provided	Nabiximols for 6 weeks	No improvement of pain and spasticity	Centonze et al. (2009)
					Patients were instructed to titrate their daily dose steadily as required over 2 weeks, to a maximum of 40 puffs/day	No modification of CD3+, CD14+, CD19+, CD56+, CD4+, CD8+ cell frequency in peripheral blood	
						No modification of FAAH or NAPE-PLD activity in circulating lymphocytes	
						No modification of CB1 or CB2 expression on CD14+, CD19+, CD56+, CD4+, CD8+ circulating cells	
100 MS patients (74 SPMS and 26 PPMS)	25/75	52 ± 7.7	No information provided	No information provided	Natural cannabis oil extract (Cannador), containing Δ9-THC:CBD 2:1 (0,25:0,125 mg)	No effect on serum levels of IFN-γ, IL-10, IL-12 or CRP	Katona et al. (2005)
						No effect on frequency of circulating IFN-γ-expressing CD3+ T cells	
					Treatment duration was 15 weeks, and doses were adjusted according to side effects, with maximal oral dose of 0,25 mg/kg/day of Δ9-THC		
16 MS patients (10 SPMS and 6 PPMS)	11/5	46 ± 7.9	15 ± 10.7	6.2	The following treatments were administered to all patients in a two-fold crossover study, separated by 4-weeks washout: Dronabinol ((−)-*trans*-Δ9-THC, 2,5 mg);	All treatments had no effects on ex vivo PHA-, anti-CD2/anti-CD28-, anti-CD3/anti-CD28- or anti-CD3-induced proliferation of T cells (but data not shown), or on circulating leukocyte subsets (CD4, CD8, CD14, CD15, CD16, CD19, CD45RA, CD45RO and CD56 (but data not shown) or on plasma levels of TNF-α, IL-12p40, IL-12p70 and IL-10	Killestein et al. (2003)
					*C. sativa* whole plant standardized extract (Δ9-THC 2,5 mg, 20–30% CBD, <5% other cannabinoids);		
					Placebo (containing oil vehicle only)		
					Doses were one capsule twice a day for two weeks and two capsules twice a day for another 2 weeks	Treatment with *C. sativa* whole plant standardized extract (but not other treatments) increased TNF-α production in ex vivo LPS-stimulated whole blood	
						7 MS patients with adverse event scores above median had also an increase in plasma IL-12p40	
Ex vivo*/*In vitro							
PBMC from 3 HC, 18 MS patients naïve to nabiximols and 11 MS patients treated with nabiximols for (mean ± SD) 29.1 ± 8.2 months (5 ± 2 puffs/day)	HC: 2/1 MS (naïve): 7/11 MS (nabiximols): 5/6	HC: 37.1 ± 7.0 MS (naïve): 44.6 ± 12.4 MS (nabiximols): 57.4 ± 6.9	MS (naïve): 9.1 ± 8.5 MS (nabiximols): 26.9 ± 14.1	MS (naïve): 4.4 (1–8.5) MS (nabiximols): 6.9 (5–8)	30 min pre-treatment with nabiximols (1, 5 and 20 μM) + stimulation with LPS or ConA for 12 h	Dose-dependent inhibition of TNF-α release in cells from HC and from MS patients, with no differences between naïve and treated with nabiximols. Similar results were observed for IL-6 and IL-10 (but data not shown)	Sorosina et al. (2018)
PBMC from 7 HC and 7 RRMS patients	HC: no information provided. RRMS: 1/6	HC: no information provided. RRMS: 40.7 ± 12.5	No information provided	2.6 (1.5–4)	CBD (1–20 μg/mL) + PHA (10 μg/mL)	CBD (2,5–20 μg/mL) suppressed proliferation in PBMC from MS patients more effectively than in PBMC from HC	Zgair et al. (2017)
PBMC from 10 HC, 4 RRMS patients, 2 SPMS patients	HC: no information provided. RRMS: 0/4 SPMS: 0/2	HC: no information provided. RRMS: 42.8 ± 13.1 SPMS: 71.5 ± 3.5	No information provided	RRMS: 2.9 (2–4.5) SPMS: 6 (5.5–6.5)	30 min pre-incubation with CBD (1–20 μg/mL) + PMA/ionomycin (concentrations not provided)	Decreased TNF-α-, IFN-γ-, and IL-17A-expressing CD3+ T cells in PBMC from HC at 20 μg/mL and in PBMC from MS patients at 2,5 μg/mL	Zgair et al. (2017)
						Decreased IL-2- and GM-CSF-expressing CD3+ T cells in PBMC from HC at 5 μg/mL and in PBMC from MS patients at 1–2,5 μg/mL	

Abbreviations: *CB1* cannabinoid receptor type 1, *CB2* cannabinoid receptor type 2, *ConA* concanavalin A, *CRP* C-reactive protein, *FAAH* fatty acid amide hydrolase, G*M-CSF* granulocyte-macrophage colony stimulating factor, *HC* healthy control, *IFN-β1b* interferon beta-1b, *IFN-γ* interferon-gamma, *IL* interleukin, *LPS* lipopolysaccharide, *MAPK14* mitogen-activated protein kinase 14, *MS* multiple sclerosis, *NAPE-PLD* N-acyl phosphatidylethanolamine phospholipase D, *NFKB1* nuclear factor kappa B subunit 1, *PBMC* peripheral blood mononuclear cells, *PHA* phytohaemagglutinin, *PPMS* primary progressive multiple sclerosis, *RPMS* relapsing-remitting multiple sclerosis, *RPS3* ribosomal protein S3, *SPMS* secondary progressive multiple sclerosis, *TNF-α* tumor necrosis factor-alpha, *TP53* tumor protein p53

Out of the five studies in patients, three were performed in small groups of subjects treated with nabiximols (a specific Cannabis extract approved in 2010 as a botanical drug with the trade name of Sativex to treat spasticity and pain in MS, and which is administered by mouth spray containing 2,7 mg of Δ9-THC and 2,5 mg of CBD per puff). All the three studies were observational, nabiximols being given according to approved indications for periods of 4–6 weeks (Sorosina et al. 2018; Santoro et al. 2017; Centonze et al. 2009). As such, they included patients with different types of MS (for instance, RRMS, PPMS, SPMS in the study by Sorosina et al. (2018)), or both untreated and treated with IFN-β (for instance, in the study by Santoro et al. (2017)). None of these studies reported any significant effect on peripheral immunity, and in particular Centonze et al. (2009) included detailed results on the immune profile of the 20 patients recruited, showing no modification of either CD3+, CD14+, CD19+, CD56+, CD4+, or CD8+ cell frequency in peripheral blood, as well as no modification of CB1 or CB2 expression on those same cells. Quite interestingly, Centonze et al. (2009) also reported no efficacy of nabiximols on pain or spasticity in their patient cohort. In this regard, Sorosina et al. (2018) in their study performed an analysis of MS patients with spasticity responding to nabiximols, reporting in whole blood upregulation of genes belonging to the ribosome pathway and downregulation of genes related to immune system, cell motility/migration and nervous system.

The remaining two studies are on the contrary clinical trials aimed at evaluating the effects of cannabinoids on MS symptoms. Neither studies employed pure CBD as test drug, nonetheless they were included in the analysis since both employed preparations containing significant amounts of CBD and reported data on patients’ peripheral immune functions. The first one (Killestein et al. 2003) is a crossover study including 16 MS patients (10 with SPMS and 6 with PPMS), receiving the following treatments each for 4 consecutive weeks, separated by 4-weeks washout: dronabinol, *C. sativa* whole plant standardized extract (containing THC 2.5 mg and 20–30% CBD), and placebo. All treatments had no effects either on the frequency of circulating T and B cells, monocytes and NK cells, or on plasma levels of TNF-α, IL-12p40, IL-12p70 and IL-10, or on ex vivo proliferation of T cells. Remarkably, treatment with the *C. sativa* whole plant extract resulted in increased TNF-α production in ex vivo LPS-stimulated whole blood, and 7 MS patients with dronabinol- and/or *C. sativa* whole plant extract-related adverse event scores above median had also an increase in plasma IL-12p40 (Killestein et al. 2003).

The second one (Katona et al. 2005) reports data derived from the Cannabinoids in MS (CAMS) study, a large randomized controlled trial to evaluate the therapeutic efficacy of cannabinoids (Zajicek et al. 2005). In the original study, 630 patients with stable MS with muscle spasticity from 33 UK centres were randomised to receive oral Δ9-THC, a whole plant extract standardized to Δ9-THC:CBD 2:1 (0,25:0,125 mg, Cannador), or placebo. Results of the whole study showed evidence of a small treatment effect on muscle spasticity (Zajicek et al. 2005). Katona et al. (2005) report data from 100 of those patients (74 SPMS and 26 PPMS), showing no effect on serum levels of IFN-γ, IL-10, IL-12 or CRP, or on frequency of circulating IFN-γ-expressing CD3+ T cells.

Ex vivo*/*in vitro studies include a report showing that nabiximols dose-dependently reduces TNF-α, IL-6 and IL-10 release in cultured PBMC from both healthy subjects and from MS patients, ether untreated and treated with nabiximols for pain and spasticity (Sorosina et al. 2018), as well as an investigation showing that CBD in the μM concentration range suppressed proliferation, decreased TNF-α-, IFN-γ-, and IL-17A-expressing CD3+ T cells as well as IL-2- and GM-CSF-expressing CD3+ T cells more effectively in cells from MS patients than from healthy subjects (Zgair et al. 2017). In both studies, CBD alone (Zgair et al. 2017) or together with Δ9-THC (Sorosina et al. 2018), was active in the μM concentration range.

## Discussion

Several lines of evidence strongly support the general immunomodulatory properties of CBD, which is an established anti-inflammatory agent endowed even with some immunosuppressive properties (reviewed in Nichols and Kaplan (2020) and in Peyravian et al. (2020)). In agreement with such favourable premises, our systematic review retrieved a total of 20 in vivo and ex vivo*/*in vitro studies of CBD in preclinical models of MS, all in rodents and including several different animal models of EAE, consistently pointing to CBD as effective in reducing the clinical and histological severity of EAE in animals, as well as to inhibit relevant encephalitogenic cellular activities in in vitro models. On the contrary, just a few studies could be identified in the clinical setting, the vast majority of them reporting no effects on immune profiles or functions. Such a major discrepancy between preclinical and clinical studies requires careful consideration, in order to identify likely explanations.

Most of the animal studies were performed in C57BL/6 J mice immunized with MOG_35–55_, a chronic animal model of MS which resembles primary and secondary progressive MS, and which mostly involves CD8+, CD4+, Th17, and regulatory T cells, B cells, as well as monocytes and macrophages (Procaccini et al. 2015; Kipp et al. 2017). CBD was however also effective in SJL/J mice immunized with PLP_139–151_, which better recapitulates relapsing–remitting MS, as well as in SJL/J mice with TMEV-induced demyelinating disease and in C57Bl/6 mice with cuprizone-induced demyelination, which involve oligodendrocytes, astrocytes, and microglia, and allow the study of axonal damage and of inflammatory-induced demyelination and remyelination processes (Procaccini et al. 2015; Kipp et al. 2017). In summary, the efficacy of CBD has been documented in the most relevant animal models of MS, which are representative of the different clinical types of disease, involve both peripheral and central immune mechanisms, and are well established for the preclinical testing of therapeutic agents.

In comparison to in vivo studies in animals, ex vivo/in vitro studies with CBD are just a few, and the majority of them is performed on encephalitogenic T lymphocytes from lymph nodes or spleen of mice with (MOG_35–55_)-induced EAE, and only one study tested CBD on astrocytes from TMEV-induced demyelinating disease SJL/J mice (Mecha et al. 2013). No information exists so far therefore on the possible direct effects of CBD on other peripheral immune cells involved in MS such as CD8+ T cells, B cells, monocytes and macrophages, nor on other CNS resident immune cells such as oligodendrocytes, or microglia. Moreover, no studies so far tested CBD on the differentiation and function of CD4+ T cell lineages such as leading to autoimmunity in MS, such as Th1 and Th17, or playing protective roles, such as Th2 and Treg, despite preliminary evidence that CBD may downregulate molecular pathways leading to Th17 (Kozela et al. 2016a).

In spite of consistent preclinical evidence, studies in MS patients are scarce and affected by major limitations, which include, besides limited sample sizes and observational designs in most of them, lack of clinically relevant endpoints, short treatment durations and doses likely insufficient to affect targets and mechanisms involved in MS pathogenesis and progression. Against this background, it is not at all surprising that results obtained in MS patients were usually negative. Indeed, all the five studies in MS patients assessed just a few parameters related to the peripheral immune profile and function, and none of them included endpoints related to disease activity and/or disability progression. While it can be argued that no clinically relevant effects would follow without underlying modifications of immune functions, the main question is why no immune effects occurred in MS patients, despite extensive and convincing evidence about the activity of CBD in animal models, and even in vitro in human cells (Zgair et al. 2017; Sorosina et al. 2018). In this regard, detailed analysis of preclinical studies suggests that the key issue could be CBD dose levels. In animal models, CBD doses reducing EAE severity were consistently at least 5 mg/kg/day or higher. Although no studies assessed plasma and/or tissue levels of CBD, considering that treatments were usually administered i.p., a very rough estimation of tissue (peak) concentrations might be in the 10–15 μM range. Such an estimate is consistent with results from in vitro experiments, where 0,1–10 μM CBD was commonly used. Remarkably, at those concentrations CBD is effective on encephalitogenic cells from rodents (Kozela et al. 2011, 2013, 2015, 2016a; Mecha et al. 2013; González-García et al. 2017; Yang et al. 2019) as well as on T cells from healthy subjects and MS patients (Zgair et al. 2017; Sorosina et al. 2018).

In clinical studies, on the contrary, CBD doses were consistently lower. In studies where nabiximols was used, a maximum of 40 puffs/day was administered by Centonze et al. (2009), while Sorosina et al. (2018) and Santoro et al. (2017) used lower daily doses. Nabiximols contains 2,5 mg of CBD per puff, which makes 100 mg/day (or about 1,4 mg/kg/day for a 70-kg subject). Katona et al. (2005) administered natural cannabis oil extract with maximal oral dose of 0,25 mg/kg/day of Δ9-THC. The oil extract contains Δ9-THC:CBD 2:1, thus it is inferred that the maximal oral dose of CBD was 0,125 mg/kg/day (or about 8,75 mg/day for a 70-kg subject). Finally, Killestein et al. (2003) used a *C. sativa* whole plant extract standardized to Δ9-THC 2,5 mg/capsule, with 20–30% CBD, and administered two capsules twice a day, which makes a total of 10 mg/day Δ9-THC and a putative 2–3 mg/day CBD. Available pharmacokinetic studies in humans (reviewed by Millar et al. 2018) show that administration of CBD, either by oromucosal spray in 5 to 20 mg doses (but, at least in one study, also up to 60–90 mg) or by oral capsules containing CBD 10 mg, consistently provide peak plasma concentration in the 1–4 ng/mL range, corresponding to about 0,01 μM, thus well below theoretical concentrations reached in animal studies as well as, most importantly, well below concentrations which are effective in in vitro models based on either animal or human cells.

On these basis, it is proposed that – for CBD to be effective in humans as an immunomodulatory drug – higher doses should be considered. Indeed, also from a general point of view which doses of CBD are more effective in different disease states remain a matter of debate, nonetheless a recent review investigating what doses have been applied in clinical populations in a variety of medical contexts showed that CBD was well tolerated at oral doses up to 50 mg/kg/day (Millar et al. 2019), corresponding to a total amount 3,5 g/day for a 70-kg subject. Recently the U.S. FDA and the EMA recently approved CBD (as Epidiolex®, GW Pharmaceuticals) to treat rare forms of epilepsy in children, with maximum doses of 10 mg/kg twice a day. Remarkably, a recent study in children and adults with treatment-refractory epilepsy showed that Epidiolex® could be safely increased up to a maximum dosage of 50 mg/kg/day depending on tolerance and seizure control, with a positive linear correlation between CBD dosage (range from 5 to 50 mg/kg/day) and level (range from 7.1 to 1200 ng/mL) (Szaflarski et al. 2019). The concentration of 1200 ng/mL corresponds to about 3,8 μM, thus quite close to the about 8 μM CBD which was shown by Zgair et al. (2017) to suppress proliferation and proinflammatory cytokine production in CD3+ T cells from MS patients. The study by Szaflarski et al. (2019) should thus be taken as a proof of concept that CBD concentrations, which in vitro exert immunomodulatory effects relevant for MS, can be safely reached in humans provided that appropriate doses are used. Studying the peripheral immune profile and function in people with epilepsy receiving Epidiolex®, and in particular in those on high dose regimens, could also provide useful information to properly design clinical studies of CBD as immune modulator in MS patients, in terms of dosing regimens as well as of relevant endpoints to be measured.

CBD has a complex pharmacological profile (Table 1), however the molecular targets acted upon by CBD were examined in just a few studies, and only in in vitro models based on rodent cells. Available results suggest no involvement of either CB1, CB2, 5-HT1A, TRPV1 or PPARγ in CBD-dependent reduction of IL-17 secretion from T cells (Kozela et al. 2013), or of CB1 or CB2 in CBD-induced inhibition of T cell proliferation (Kozela et al. 2011), or of CB1, CB2 or GPR55 in CBD-induced inhibition of MOG_35–55_/IL-12-induced IL-6 secretion and increased apoptosis in mouse encephalitogenic spleen cells (González-García et al. 2017). The only positive evidence presently available suggests a role for A_2A_ receptors in CBD-induced reduction of CCL2 secretion from mouse astrocytes (Mecha et al. 2013). In this regard, it may be of interest that EHP-101, a new chemical entity derived from CBD, acting as dual PPARγ and CB2 agonist as well as activator of the hypoxia inducible factor (HIF) pathway, has been shown to exert anti-inflammatory effects in vitro in murine RAW264.7 and BV2 cell lines and rat primary microglia cells, and to reduce EAE severity in C57BL/6 J mice with either (MOG_35–55_)-induced EAE or with cuprizone-induced demyelination, as well as in the TMEV-IDD SJL/J mouse model (Navarrete et al. 2018, 2020). Taken as a whole, available evidence does not allow any meaningful conclusion about molecular targets involved in the effects of CBD in EAE and possibly in MS, unless that apparently its therapeutic potential cannot be explained just by means of a single target. Meanwhile, evidence about the activity of synthetic derivatives of CBD, such as HU-446 and HU-465 which exert inhibitory effects on encephalitogenic MOG_35–55_-specific T cell line from lymph nodes of C57BL/6 mice (Kozela et al. 2016b), emphasize the relevance of CBD also as a molecular scaffold to develop novel drugs targeting the immune system.

In summary, available preclinical evidence in rodent models of EAE strongly support CBD as an effective immunomodulating and disease-modifying drug, although its cellular and molecular targets remain largely uninvestigated. In contrast, despite the established use of CBD-containing drugs in MS, evidence in patients is limited and usually negative, possibly due mainly to inadequate therapeutic regimens, in terms of both dose and duration. A research agenda aiming at the proper assessment of CBD as an immunomodulating drug for MS should include, first of all, a detailed characterization of the effects of CBD on the key cellular and molecular mechanisms involved in MS pathogenesis and progression, including for example: (i) peripheral activation of pro-inflammatory T cells resulting from their interaction with antigen-presenting cells, such as macrophages; (ii) migration of activated T cells through the blood–brain barrier, mediated by adhesion molecules, proteases and chemokines; (iii) reactivation of T cells in the CNS through interaction with microglia, with subsequent secretion of pro-inflammatory cytokines, such as IFN-γ or IL-2, leading to activation of macrophages, other T cells and B cells; (iv) inflammation-induced damage of oligodendrocytes, resulting in destruction of the myelin sheath by cytotoxic mediators, such as TNF-α and oxidative radicals; (v) differentiation of B cells into plasma cells, secreting demyelinating antibodies in turn attracting macrophages, and triggering the complement cascade (Yamout and Alroughani 2018; Hemmer et al. 2002). Only fragmentary evidence exists so far, nearly only in T cells and mostly in rodent models, and much more work is needed, primarily in human cells. The most important and urgent needs regards however the development of well-designed clinical trials, aimed at testing adequate doses of CBD on clinically relevant efficacy endpoints Indeed, based on available pharmacokinetic and therapeutic studies in other disease conditions, and in particular in epilepsy, doses higher than those used so far should be tested to properly assess the immunomodulatory potential of CBD in MS. Future studies should always include careful monitoring of plasma concentration in relation to dosing regimens, to collect key information which will allow to deal with the inherent pharmacokinetic heterogeneity of CBD, which is likely due at least in part to pharmacogenetic factors. Most importantly, such trials should include as primary efficacy endpoints clinically relevant measures of disease activity and/or disability progression, or at least evidence of magnetic resonance imaging-assessed disease activity, relapses and progression, neurological rating scales, measures of cognitive impairment, fatigue scales, as assessed by patient and physician, as well as patient reported outcomes (CHMP, 2015). Nevertheless, even based on the limited evidence so far available, CBD appears as a highly promising drug with significant immunomodulating and disease-modifying potential for MS, added benefits residing in its well established safety and tolerability profile.

## Supplementary Information

ESM 1 (PDF 1304 kb)
