# Supplementary material for: Immunomodulatory Potential of Cannabidiol in Multiple Sclerosis: a Systematic Review
Source: J Neuroimmune Pharmacol. 2021 Jan 25;16(2):251–69. doi: 10.1007/s11481-021-09982-7 (PMC7829325; doi:10.1007/s11481-021-09982-7)
Supplement: Supplementary file 1 — (PDF 1304 kb) [file 11481_2021_9982_MOESM1_ESM.pdf]

Supplementary Table S1. References retrieved on PubMed, Scopus, Web of Science.

Ref #	Citation	Database	Decision
1.	A breakthrough for the treatment of spasticity in multiple sclerosis<br>Lebrun C, Vermersch P.","Rev Neurol (Paris). 2015<br>171(4):327-8. doi: 10.1016/j.neurol.2015.03.002.	PubMed<br>Scopus	Discard<br>Reasons:<br>Not of interest
2.	A brief history of cannabinoid and endocannabinoid pharmacology as inspired by the work of British scientists.<br>Di Marzo V.<br>Trends in Pharmacological Sciences. 2006<br>27(3):134-140. doi:10.1016/j.tips.2006.01.010	Scopus	Discard<br>Reasons:<br>Not of interest
3.	A Cannabigerol Quinone Alleviates Neuroinflammation in a Chronic Model of Multiple Sclerosis.<br>Granja AG, Carrillo-Salinas F, Pagani A, Gomez-Canas M, Negri R, Navarrete C, Mecha M, Mestre L, Fiebich, BL, Cantarero I, Calzado, MA, Bellido ML, Fernandez-Ruiz J, Appendino G, Guaza C, Munoz E.<br>JOURNAL OF NEUROIMMUNE PHARMACOLOGY. 2012<br>7(4):1002-1016. doi: 10.1007/s11481-012-9399-3	Pubmed<br>Scopus<br>Web of Science	Discard<br>Reasons:<br>Not of interest
4.	A cost-effectiveness model for the use of a cannabis-derived oromucosal spray for the treatment of spasticity in multiple sclerosis<br>Gras A, Broughton J.<br>Expert Rev Pharmacoecon Outcomes Res. 2016<br>16(6):771-779 doi:10.1586/14737167.2016.1140574	Pubmed<br>Scopus<br>Web of Science	Discard<br>Reasons:<br>Not of interest
5.	A Cross-Sectional Study of the Impact of Spasticity on Daily Activities in Multiple Sclerosis<br>Bethoux F, Marrie RA<br>Patient. 2016<br>9(6):537-546. doi:10.1007/s40271-016-0173-0.	Scopus	Discard<br>Reasons:<br>Not of interest
6.	A double-blind, randomized, placebo-controlled, parallel group study of THC/CBD spray in peripheral neuropathic pain treatment.<br>Serpell M, Ratcliffe S, Hovorka J, Schofield M, Taylor L, Lauder H, Ehler E.<br>European Journal of pain 2014<br>18(7): 999-1012. doi: 10.1002/j.1532-2149.2013.00445.x	Web of Science	Discard<br>Reasons:<br>Not of interest

7.	A double-blind, randomized, placebo-controlled, parallel-group study of THC/CBD oromucosal spray in combination with the existing treatment regimen, in the relief of central neuropathic pain in patients with multiple sclerosis.<br>Langford RM, Mares J, Novotna A, Vachova M, Novakova I, Notcutt W, Ratcliffe S.<br>J Neurol. 2013<br>260(4):984-97. doi: 10.1007/s00415-012-6739-4.	Pubmed<br>Scopus<br>Web of Science	Discard<br>Reasons:<br>Not of interest
8.	A double-blind, randomized, placebo-controlled, parallel-group study of Sativex, in subjects with symptoms of spasticity due to multiple sclerosis.<br>Collin C, Ehler E, Waberzinek G, Alsindi Z, Davies P, Powell K, Notcutt W, O'Leary C, Ratcliffe S, Nováková I, Zapletalova O, Piková J, Ambler Z.<br>Neurol Res. 2010<br>32(5):451-9. doi: 10.1179/016164109X12590518685660.	Pubmed<br>Scopus<br>Web of Science	Discard<br>Reasons:<br>Not of interest
9.	A drug discovery case history of 'delta-9- tetrahydrocannabinol, cannabidiol.<br>Tanasescu R, Rog D, Constantinescu CS<br>Expert Opinion on Drug Discovery. 2011<br>6(4):437-452. doi:10.1517/17460441.2011.560935.	Scopus<br>Web of Science	Discard<br>Reasons:<br>Not of interest
10.	A growing concern.<br>Lancet Neurology. 2006<br>5(1):1. doi:10.1016/S1474-4422(05)70255-6.	Scopus	Discard<br>Reasons:<br>Not of interest
11.	A man with MS asking about medicinal cannabis.<br>Huynh W, Kiernan MC<br>Medicine Today. 2017<br>18(7):56-59.	Scopus	Discard<br>Reasons:<br>Not of interest
12.	A multicentre, open-label, follow-on study to assess the long-term maintenance of effect, tolerance and safety of THC/CBD oromucosal spray in the management of neuropathic pain.<br>Hoggart B, Ratcliffe S, Ehler E, Simpson KH, Hovorka J, Lejcko J, Taylor L, Lauder H, Serpell M.<br>Jornal of Neurology 2015<br>262(1):27-40. doi: 10.1007/s00415-014-7502-9.	Web of Science	Discard<br>Reasons:<br>Not of interest
13.	A Multiple-Dose, Randomized, Double-Blind, Placebo-Controlled, Parallel-Group QT/QTc Study to Evaluate the Electrophysiologic Effects of THC/CBD Spray.<br>Sellers EM, Schoedel K, Bartlett C, Romach M, Russo EB, Stott CG, Wright S, White L, Duncombe P, Chen CF<br>Clin Pharmacol Drug Dev. 2013<br>(3):285-94. doi: 10.1002/cpdd.36.	Pubmed<br>Scopus<br>Web of Science	Discard<br>Reasons:<br>Not of interest

14.	A new formulation of cannabidiol in cream shows therapeutic effects in a mouse model of experimental autoimmune encephalomyelitis.<br>Giacoppo S, Galuppo M, Pollastro F, Grassi G, Bramanti P, Mazzon E.<br>Daru. 2015<br>23:48. doi: 10.1186/s40199-015-0131-8.	Pubmed<br>Scopus<br>Web of Science	Keep
15.	A new multiple sclerosis spasticity treatment option: effect in everyday clinical practice and cost-effectiveness in Germany.<br>Flachenecker P.<br>Expert Rev Neurother. 2013<br>13(3 Suppl 1):15-9. doi: 10.1586/ern.13.1.	Pubmed<br>Scopus<br>Web of Science	Discard<br>Reasons:<br>Not of interest
16.	A novel analgesics made from Cannabis [Uj típusú analgetikum cannabisból.]<br>Szendrei K.<br>Ideggyógyászati szemle. 2004<br>57(1-2):36-40.	Pubmed<br>Scopus	Discard<br>Reasons:<br>Not of interest
17.	A phase I study to assess the single and multiple dose pharmacokinetics of THC/CBD oromucosal spray.<br>Stott CG, White L, Wright S, Wilbraham D, Guy GW.<br>European Journal of Clinical Pharmacology 2013<br>69(5): 1135-1147. doi: 10.1007/s00228-012-1441-0.	Web of Science	Discard<br>Reasons:<br>Not of interest
18.	A Phase I, double blind, three-way crossover study to assess the pharmacokinetic profile of cannabis based medicine extract (CBME) administered sublingually in variant cannabinoid ratios in normal healthy male volunteers (GWPK0215).<br>Guy G.W, Robson P.J.<br>Journal of Cannabis Therapeutics. 2003<br>3(4):121-152. doi:10.1300/J175v03n04_02	Scopus	Discard<br>Reasons:<br>Not of interest
19.	A phase I, open label, four-way crossover study to compare the pharmacokinetic profiles of a single dose of 20 mg of a Cannabis Based Medicine Extract (CBME) administered on 3 different areas of the Buccal Mucosa and to investigate the pharmacokinetics of CBME per oral in healthy male and female volunteers (GWPK0112)<br>Journal of Cannabis Therapeutics. 2004<br>3(4):79-120. doi:10.1300/J175v03n04_01	Scopus	Discard<br>Reasons:<br>Not of interest
20.	A Phase I, open-label, randomized, crossover study in three parallel groups to evaluate the effect of Rifampicin, Ketoconazole, and Omeprazole on the pharmacokinetics of THC/CBD oromucosal spray in healthy volunteers.<br>Stott C, White L, Wright S, Wilbraham D, Guy G.<br>Springerplus 2013<br>2(1):236. doi:10.1186/2193-1801-2-236	Web of Science	Discard<br>Reasons:<br>Not of interest

21.	A placebo-controlled, parallel-group, randomized withdrawal study of subjects with symptoms of spasticity due to multiple sclerosis who are receiving long-term SativexÂ® (nabiximols).<br>Notcutt W, Langford R, Davies P, Ratcliffe S, Potts R.<br>Mult Scler. 2012<br>18(2):219-28. doi: 10.1177/1352458511419700.	Pubmed<br>Scopus<br>Web of Science	Discard<br>Reasons:<br>Not of interest
22.	A potted history<br>Pain S.<br>Nature. 2015<br>525, S10-S11. doi:10.1038/525S10a	Pubmed<br>Scopus	Discard<br>Reasons:<br>Not of interest
23.	A preliminary controlled study to determine whether whole-plant cannabis extracts can improve intractable neurogenic symptoms.<br>Wade DT, Robson P, House H, Makela P, Aram<br>J. Clin Rehabil. 2003<br>17(1):21-9 doi:10.1191/0269215503cr581oa	Pubmed<br>Scopus<br>Web of Science	Discard<br>Reasons:<br>Not of interest
24.	A questionnaire survey of patients and carers of patients prescribed Sativex as an unlicensed medicine.<br>Notcutt WG.<br>Prim Health Care Res Dev. 2013<br>14(2):192-9. doi: 10.1017/S1463423612000333	Pubmed<br>Scopus	Discard<br>Reasons:<br>Not of interest
25.	A randomised controlled cross-over double-blind pilot study protocol on THC:CBD oromucosal spray efficacy as an add-on therapy for post-stroke spasticity.<br>Marinelli L, Balestrino M, Mori L, Puce L, Rosa GM, Giorello L, Currá A, Fattapposta F, Serrati C, Gandolfo C, Abbruzzese G, Trompetto C.<br>BMJ Open. 2017<br>7(9):e016843. doi: 10.1136/bmjopen-2017-016843	Pubmed<br>Scopus<br>Web of Science	Discard<br>Reasons:<br>Not of interest
26.	A randomized, double-blind, placebo-controlled, parallel-group, enriched-design study of nabiximols* (Sativex(Â®)), as add-on therapy, in subjects with refractory spasticity caused by multiple sclerosis.<br>Novotna A, Mares J, Ratcliffe S, Novakova I, Vachova M, Zapletalova O, Gasperini C, Pozzilli C, Cefaro L, Comi G, Rossi P, Ambler Z, Stelmasiak Z, Erdmann A, Montalban X, Klimek A, Davies P, Sativex Spasticity Study Group<br>Eur J Neurol. 2011<br>18(9):1122-31. doi: 10.1111/j.1468-1331.2010.03328.x	Pubmed<br>Scopus<br>Web of Science	Discard<br>Reasons:<br>Not of interest
27.	A reliable and validated LC-MS/MS method for the simultaneous quantification of 4 cannabinoids in 40 consumer products.<br>Meng QF, Buchanan B, Zuccolo J, Poulin MM, Gabriele J, Baranowski DC.	Web of Science	Discard<br>Reasons:<br>Not of interest

	PLoS One. 2018<br>13(5): e0196396. doi: 10.1371/journal.pone.0196396.		
28.	A Review of Recent Advances in the Therapeutic Uses of Secondary Cannabinoids.<br>Morabito D, Soyster P, Ramey-Wright S, Belendiuk KA, Bonn-Miller MO<br>Current Addiction Reports 2016<br>3(2):230-238. doi:10.1007/s40429-016-0096-9	Scopus	Discard<br>Reasons:<br>Not of interest
29.	A review of scientific evidence for THC:CBD oromucosal spray (nabiximols) in the management of chronic pain<br>Überall MA<br>Journal of Pain Research 2020<br>13: 399-410. doi: 10.2147/JPR.S240011	Scopus<br>Web of Science	Discard<br>Reasons: Not of interest
30.	A review of the cultivation and processing of cannabis (Cannabis sativa L.) for production of prescription medicines in the UK.<br>Potter DJ<br>Drug Test Anal. 2014<br>6(1-2):31-8. doi: 10.1002/dta.1531.	Pubmed<br>Scopus<br>Web of Science	Discard<br>Reasons:<br>Not of interest
31.	A review of the effects of baclofen and of THC:CBD oromucosal spray on spasticity-related walking impairment in multiple sclerosis.<br>Arroyo González R.<br>Expert Rev Neurother. 2018<br>18(10):785-791. doi: 10.1080/14737175.2018.1510772.	Pubmed<br>Scopus<br>Web of Science	Discard<br>Reasons:<br>Not of interest
32.	A review on agronomic, phytochemical and pharmacological aspects of cannabis (Cannabis sativa L.).<br>Asadi S, Moghadam H, Naghdi Badi H, Naghdi Badi H, Naghavi MR, Salami SAR<br>Journal of Medicinal Plants. 2019<br>2(70):1-20.	Scopus	Discard<br>Reasons:<br>Not of interest
33.	A Sativex(®)-like combination of phytocannabinoids as a disease-modifying therapy in a viral model of multiple sclerosis.<br>Feliu A, Moreno-Martet M, Mecha M, Carrillo-Salinas FJ, de Lago E, Fernandez-Ruiz J, Guaza C.<br>Br J Pharmacol. 2015<br>172(14):3579-3595. doi: 10.1111/bph.13159	Pubmed<br>Web of Science	Discard<br>Reasons:<br>Not of interest
34.	A Scoping Review on Clinical Trials of Pain Reduction With Cannabis Administration in Adults<br>Haleem R, Wright R<br>J Clin Med Res. 2020<br>12(6): 344–351. doi: 10.14740/jocmr4210	Pubmed	Discard<br>Reasons: Not of interest

35.	A single centre, placebo-controlled, four period, crossover, tolerability study assessing, pharmacodynamic effects, pharmacokinetic characteristics and cognitive profiles of a single dose of three formulations of cannabis based medicine extracts (CBMEs) (GWPD9901), plus a two period tolerability study comparing pharmacodynamic effects and pharmacokinetic characteristics of a single dose of a cannabis based medicine extract given via two administration routes (GWPD9901 EXT).<br>Guy G.W, Flint M.E.<br>Journal of Cannabis Therapeutics 2004<br>3(3):35-77. doi:10.1300/J175v03n03_03	Scopus	Discard<br>Reasons:<br>Not of interest
36.	A single dose of cannabidiol reduces blood pressure in healthy volunteers in a randomized crossover study.<br>Jadoon KA, Tan GD, O'Sullivan SE.<br>JCI Insight. 2017<br>2(12). doi: 10.1172/jci.insight.93760.	Pubmed<br>Scopus<br>Web of Science	Discard<br>Reasons:<br>Not of interest
37.	A systematic review of pharmacological pain management in multiple sclerosis.<br>Jawahar R, Oh U, Yang S, Lapane KL<br>Drugs. 2013<br>73(15):1711-1722. doi:10.1007/s40265-013-0125-0.	Scopus	Discard<br>Reasons:<br>Not of interest
38.	A systematic review of the effect of cannabidiol on cognitive function: Relevance to schizophrenia.<br>Osborne AL, Solowij N, Weston-Green K.<br>Neuroscience and Biobehavioral Reviews 2017<br>72, 310-324. doi: 10.1016/j.neubiorev.2016.11.012	Web of Science	Discard<br>Reasons:<br>Not of interest
39.	A systematic review of the evidence for medical marijuana in psychiatric indications.<br>Wilkinson ST, Radhakrishnan R, D'Souza DC.<br>Journal of Clinical Psychiatry. 2016<br>77(8):1050-1064 doi:10.4088/JCP.15r10036	Scopus	Discard<br>Reasons:<br>Not of interest
40.	A tale of two cannabinoids: the therapeutic rationale for combining tetrahydrocannabinol and cannabidiol.<br>Russo E, Guy GW.<br>Med Hypotheses 2006<br>66(2):234-46.	Pubmed<br>Scopus<br>Web of Science	Discard<br>Reasons:<br>Not of interest
41.	A urinary test procedure for identification of cannabidiol in patients undergoing medical therapy with marijuana.<br>Wertlake PT, Henson MD.<br>J Pain Res. 2016<br>9, 81-5. doi: 10.2147/JPR.S96856.	Pubmed<br>Scopus<br>Web of Science	Discard<br>Reasons:<br>Not of interest

42.	Abuse potential and psychoactive effects of delta-9-tetrahydrocannabinol and cannabidiol oromucosal spray (Sativex), a new cannabinoid medicine.<br>Robson P.<br>Expert Opinion on Drug Safety 2011<br>10(5): 675-685. doi: 10.1517/14740338.2011.575778	Pubmed<br>Scopus<br>Web of Science	Discard<br>Reasons:<br>Not of interest
43.	Acute and chronic cannabinoid extracts administration affects motor function in a CREAE model of multiple sclerosis.<br>Buccellato E, Carretta D, Utan A, Cavina C, Speroni E, Grassi G, Candeletti S, Romualdi P.<br>J Ethnopharmacol. 2011<br>133(3):1033-8. doi: 10.1016/j.jep.2010.11.035	Pubmed<br>Scopus<br>Web of Science	Keep
44.	Adherence to Consolidated Standards of Reporting Trials (CONSORT) Guidelines for Reporting Safety Outcomes in Trials of Cannabinoids for Chronic Pain: Protocol for a Systematic Review.<br>Mohiuddin MM, Mizubuti G, Haroutounian S, Smith S, Campbell F, Park R, Gilron I.<br>JMIR Res Protoc. 2019<br>8(1), e11637. doi: 10.2196/11637	Pubmed<br>Web of Science	Discard<br>Reasons:<br>Not of interest
45.	Adherence to Consolidated Standards of Reporting Trials (CONSORT) Guidelines for Reporting Safety Outcomes in Trials of Medical Cannabis and Cannabis-based Medicines for Chronic Noncancer Pain A Systematic Review.<br>Mohiuddin MM, Mizubuti GB, Haroutounian S, Smith SM, Rice ASC, Campbell F, Park R, Gilron I<br>Clin J Pain 2020<br>36(4):302-319. doi: 10.1097/AJP.0000000000000807.	Web of Science	Discard<br>Reasons: Not of interest
46.	Advances in the management of MS spasticity: recent observational studies.<br>Fernàndez O.<br>Eur Neurol. 2014<br>72 Suppl 1:12-4. doi: 10.1159/000367618.	Pubmed<br>Scopus	Discard<br>Reasons:<br>Not of interest
47.	Advances in the management of MS symptoms: real-life evidence.<br>Trojano M<br>Neurodegenerative disease management.2015<br>5(6):19-21. doi:10.2217/nmt.15.66	Pubmed<br>Scopus	Discard<br>Reasons:<br>Not of interest
48.	Advances in the management of MS symptoms: recently proposed clinical management algorithms.<br>Vermersch P.<br>Neurodegener Dis Manag. 2015<br>5(6 Suppl):23-6. doi: 10.2217/nmt.15.57.	Pubmed<br>Scopus	Discard<br>Reasons:<br>Not of interest

49.	Advances in the management of multiple sclerosis spasticity: experiences from recent studies and everyday clinical practice.<br>Pozzilli C.<br>Expert Rev Neurother. 2013<br>13(12 Suppl):49-54. doi: 10.1586/14737175.2013.865877.	Pubmed<br>Scopus<br>Web of Science	Discard<br>Reasons:<br>Not of interest
50.	Advances in the management of multiple sclerosis spasticity: multiple sclerosis spasticity nervous pathways.<br>Centonze D.<br>Eur Neurol. 2014<br>72 Suppl 1:6-8. doi: 10.1159/000367615.	Pubmed<br>Scopus	Discard<br>Reasons:<br>Not of interest
51.	Advances in the management of multiple sclerosis spasticity: Multiple sclerosis spasticity guidelines<br>Gold R, Oreja-Guevara C<br>Expert Review of Neurotherapeutics. 2013<br>13(12 SUPPL.):55-59. doi:10.1586/14737175.2013.865880	Scopus	Discard<br>Reasons:<br>Not of interest
52.	Advances in the management of multiple sclerosis spasticity: recent clinical trials.<br>Fernández O.<br>Eur Neurol. 2014<br>72 Suppl 1:9-11. doi: 10.1159/000367616.	Pubmed<br>Scopus	Discard<br>Reasons:<br>Not of interest
53.	Advances in the management of multiple sclerosis-related spasticity.<br>Hartung H-P<br>Expert Review of Neurotherapeutics. 2012<br>12(4) SUPPL.:1-2. doi:10.1586/ern.12.10	Scopus	Discard<br>Reasons:<br>Not of interest
54.	Advances in the Understanding and Management of Chronic Pain in Multiple Sclerosis: a Comprehensive Review<br>Urits I, Adamian L, Fiocchi J, Hoyt D, Ernst C, Kaye AD, Viswanath O<br>Current Pain and Headache Reports. 2019<br>23(8):59. doi:10.1007/s11916-019-0800-2	Scopus	Discard<br>Reasons:<br>Not of interest
55.	Adverse and palliative effects of the cannabinoids [Efectos adversos y paliativos de los canabinoides]<br>Contreras CM, Gutierrez Garcia AGG, Saavedra M, Bernal-Morales B, Landa JFR, Hernández-Lozano M<br>Salud Mental. 2003<br>26(6):62-75.	Scopus<br>Web of Science	Discard<br>Reasons:<br>Not of interest
56.	Adverse effects from a cannabis spray<br>Brand H<br>British Dental Journal. 2007	Scopus	Discard<br>Reasons:<br>Not of interest

	203(6):336-337. doi:10.1038/bdj.2007.852		
57.	Adverse effects of medical cannabinoids: A systematic review.<br>Wang T, Collet J-P, Shapiro S, Ware MA<br>CMAJ. 2008<br>178(13):1669-1678. doi:10.1503/cmaj.071178	Scopus	Discard<br>Reasons:<br>Not of interest
58.	Adverse health effects of marijuana use<br>Volkow N, Baler RD, Compton W, Weiss SRB<br>New England Journal of Medicine. 2014<br>370(23):2219-2227. doi:10.1056/NEJMra1402309	Scopus	Discard<br>Reasons:<br>Not of interest
59.	Agonistic properties of cannabidiol at 5-HT1a receptors.<br>Russo EB, Burnett A, Hall B, Parker KK.<br>Neurochemical Research 2005<br>30(8):1037-1043. doi: 10.1007/s11064-005-6978-1	Web of Science	Discard<br>Reasons:<br>Not of interest
60.	"ALS reversals": demographics, disease characteristics, treatments, and co-morbidities.<br>Harrison D, Mehta P, van Es MA, Stommel E, Drory VE, Nefussy B, van den Berg LH, Crayle J, Bedlack R.<br>Amytrophic Lateral Sclerosis and Frontotemporal Degeneration 2018<br>19(7-8): 495-499. doi: 10.1080/21678421.2018.1457059	Pubmed<br>Scopus<br>Web of Science	Discard<br>Reasons:<br>Not of interest
61.	Alzheimer disease: Crosstalk between the canonical Wnt/beta-catenin pathway and PPARs alpha and gamma<br>Vallèe A, Lecarpentier Y<br>Frontiers in Neuroscience. 2016<br>459(50). doi:10.3389/fnins.2016.00459	Scopus	Discard<br>Reasons:<br>Not of interest
62.	American academy of neurology – 70th annual meeting<br>Mila M<br>Drugs of the Future. 2018<br>43(6):457-465. doi:10.1358/dof.2018.043.06.2823637	Scopus	Discard<br>Reasons:<br>Not of interest
63.	An epileptic seizure in a patient with multiple sclerosis treated with THC/CBD (Sativex™)<br>Aparicio RR, Polo VO<br>Atencion Farmaceutica. 2013<br>15(6):439-442	Scopus<br>Web of Science	Discard<br>Reasons:<br>Not of interest
64.	An observational postmarketing safety registry of patients in the UK, Germany, and Switzerland who have been prescribed Sativex(®) (THC:CBD, nabiximols) oromucosal spray<br>Etges T, Karolia K, Grint T, Taylor A, Lauder H, Daka B, Wright S.<br>Ther Clin Risk Manag. 2016<br>12:1667-1675. doi: 10.2147/TCRM.S115014	Pubmed<br>Scopus<br>Web of Science	Discard<br>Reasons:<br>Not of interest

65.	An open-label pilot study of cannabis-based extracts for bladder dysfunction in advanced multiple sclerosis<br>Brady CM, DasGupta R, Dalton C, Wiseman OJ, Berkley KJ, Fowler CJ.<br>Mult Scler. 2004<br>10(4):425-33 doi:10.1191/1352458504ms1063oa	Pubmed<br>Scopus<br>Web of Science	Discard<br>Reasons:<br>Not of interest
66.	An Update of Current Cannabis-Based Pharmaceuticals in Pain Medicine<br>Urits I, Borchart M, Hasegawa M, Kochanski J, Orhurhu V, Viswanath O.<br>Pain Ther. 2019<br>8(1):41-51. doi: 10.1007/s40122-019-0114-4.	Pubmed<br>Web of Science	Discard<br>Reasons:<br>Not of interest
67.	An Update on the Management of Neurogenic Bladder<br>Sanford MT, Ginsberg DA<br>Current Bladder Dysfunction Reports. 2017<br>12(1):15-25. doi:10.1007/s11884-017-0409-4	Scopus	Discard<br>Reasons:<br>Not of interest
68.	Analytical and Pharmacological Challenges in Cannabis Research<br>Gertsch J<br>Planta Medica. 2018<br>84(4):213. doi:10.1055/s-0044-101051	Scopus	Discard<br>Reasons:<br>Not of interest
69.	Anandamide: An update<br>Smita K, Kumar VS, Premendran JS<br>Fundamental and Clinical Pharmacology 2007<br>21(1):1-8. doi:10.1111/j.1472-8206.2006.00454.x	Scopus	Discard<br>Reasons:<br>Not of interest
70.	Aplicaciones terapéuticas por acción de los cannabinoides<br>Plancarte-Sánchez R, Mansilla-Olivares A, De Los Reyes-Pacheco VA, Meneses-González F.<br>Gac Med Mex. 2019<br>155(3):307-318. doi: 10.24875/GMM.18004928	Pubmed<br>Scopus	Discard<br>Reasons:<br>Not of interest
71.	Application device for THC:CBD oromucosal spray in the management of resistant spasticity: pre-production testing.<br>Montero-Escribano P, Vila Silván C.<br>Expert Rev Med Devices. 2019<br>16(9):835-840. doi: 10.1080/17434440.2019.1653182.	Pubmed<br>Scopus<br>Web of Science	Discard<br>Reasons:<br>Not of interest
72.	Application of Medical Cannabis in Patients with Central Nerve System Disorders.<br>Kotula L, Petniak A, Kolodziej E, Amarowicz M, Urbanczuk M, Schab K, Gil-Kulik P, Karwat J, Kotula J, Mulawka P, Mulawka D, Kocki J. Modern Phytomorphology 2015<br>8: 65-70	Web of Science	Discard<br>Reasons:<br>Not of interest
73.	Assessment of blinding to treatment allocation in studies of a cannabis-based medicine (Sativex®) in people with multiple sclerosis: a new approach.	PubMed<br>Scopus	Discard<br>Reasons:

	Wright S, Duncombe P, Altman DG.<br>Trials 2012<br>13:189. doi: 10.1186/1745-6215-13-189		Not of interest
74.	Assessment of the effectiveness and safety of Sativex® in compassionate use<br>Cimas-Hernando I, Pato-Pato A, Lorenzo-GonzÃ¡lez JR, RodrÃ-guez-Constenla I.<br>Rev Neurol. 2015<br>60(5):202-6	PubMed<br>Scopus	Discard<br>Reasons:<br>Not of interest
75.	Author response<br>Robert MP, Plant GT<br>Neurology. 2014<br>83(16):1485-1486. doi:10.1212/WNL.0000000000000924	Scopus	Discard<br>Reasons:<br>Not of interest
76.	Availability and approval of cannabis-based medicines for chronic pain management and palliative/supportive care in Europe: A survey of the status in the chapters of the European Pain Federation<br>Krcevski-Skvarc N, Wells C, Häuser W.<br>Eur J Pain. 2018<br>22(3):440-454. doi: 10.1002/ejp.1147.	Pubmed<br>Scopus<br>Web of Science	Discard<br>Reasons:<br>Not of interest
77.	Avidekel Cannabis extracts and cannabidiol are as efficient as Copaxone in suppressing EAE in SJL/J mice<br>Gallily R, Yekhtin Z.<br>Inflammopharmacology 2019<br>27(1):167-173. doi: 10.1007/s10787-018-0536-3	Pubmed<br>Scopus<br>Web of Science	Keep
78.	Balance worsening associated with nabiximols in multiple sclerosis<br>Castelli L, Prosperini L, Pozzilli C.<br>Mult Scler. 2019<br>25(1):113-117. doi: 10.1177/1352458518765649.	Pubmed<br>Scopus<br>Web of Science	Discard<br>Reasons:<br>Not of interest
79.	Barriers to the wider adoption of medicinal Cannabis<br>Alexander SPH<br>British Journal of Pain 2020<br>14(2): 122-132 doi: 10.1177/2049463720922884	Scopus	Discard<br>Reasons: Not of interest
80.	Benefits and Risks of Therapeutic Cannabinoids for Neurologic Disorders<br>Patwa HS<br>Clinical Therapeutics. 2018<br>40(9):1436-1437 doi:10.1016/j.clinthera.2018.08.001	Scopus	Discard<br>Reasons:<br>Not of interest
81.	Beyond THC and Endocannabinoids<br>Pacher P, Kogan NM, Mechoulam R<br>Annual Review of Pharmacology and Toxicology 2020	Web of Science	Discard<br>Reasons: Not of interest

	60:637-659 doi: 10.1146/annurev-pharmtox-010818-021441		
82.	Big cannabis in the UK: is industry support for wider patient access motivated by promises of recreational market worth billions?<br>Gornall J<br>BMJ 2020<br>368:m1002 doi: https://doi.org/10.1136/bmj.m1002	Scopus	Discard<br>Reasons: Not of interest
83.	Bones and Joints: The Effects of Cannabinoids on the Skeleton.<br>Ehrenkranz J, Levine MA.<br>Journal Of Clinical Endocrinology & Metabolism 2019<br>104(10) 4683-4694 doi: 10.1210/jc.2019-00665	Web of Science	Discard<br>Reasons:<br>Not of interest
84.	Brain cannabinoid systems as targets for the therapy of neurological disorders<br>Consroe P<br>Neurobiology of Disease. 1998<br>5(6):534-551 doi:10.1006/nbdi.1998.0220	Scopus	Discard<br>Reasons:<br>Not of interest
85.	Brain functional MRI changes in multiple sclerosis patients treated with Tetrahydrocannabinol: Cannabidiol (THC:CBD) oromucosal spray for spasticity<br>Gajofatto A, Cardobi N, Gobbin F, Calabrese M, Barillari M, Turatti M, Benedetti MD.<br>MULTIPLE SCLEROSIS JOURNAL 2016<br>Meeting Abstract: P508 22:223-224 Supplement: 3	Web of Science	Discard<br>Reasons:<br>Not of interest
86.	Can pharmacological manipulation of LTP favor the effects of motor rehabilitation in multiple sclerosis?<br>Stampanoni Bassi M, Leocani L, Comi G, Iezzi E, Centonze D<br>Multiple Sclerosis Journal, 2018<br>24(7):902-907 doi:10.1177/1352458517721358	Scopus	Discard<br>Reasons:<br>Not of interest
87.	Cannabidiol (CBD) and its analogs: a review of their effects on inflammation.<br>Burstein S<br>Biorganic & Medical Chemistry 2015<br>23(7):1377-1385 doi: 10.1016/j.bmc.2015.01.059	Web of Science	Discard<br>Reasons:<br>Not of interest
88.	Cannabidiol (CBD) enhances lipopolysaccharide (LPS)-induced pulmonary inflammation in C57BL/6 mice<br>Karmaus PW, Wagner JG, Harkema JR, Kaminski NE, Kaplan BL.<br>J Immunotoxicol. 2013<br>10(3):321-8. doi: 10.3109/1547691X.2012.741628	Pubmed<br>Scopus<br>Web of Science	Discard Reasons: Not of interest
89.	Cannabidiol (CBD) for Treatment of Neurofibromatosis-related Pain and Concomitant Mood Disorder: A Case Report<br>Hegazy O, Platnick H<br>Cureus 2019	Scopus<br>Web of Science	Discard<br>Reasons:<br>Not of interest

	11(12): e6312. doi:10.7759/cureus.6312		
90.	Cannabidiol (CBD) induces functional Tregs in response to low-level T cell activation.<br>Dhital S, Stokes JV, Park N, Seo KS, Kaplan BLF.<br>Cellular Immunology 2017<br>312: 25-34 doi: 10.1016/j.cellimm.2016.11.006	Web of Science	Discard Reasons: Not of interest
91.	Cannabidiol and Multiple Sclerosis<br>Mecha M, Feliu A, Carrillo-Salinas FJ, Guaza C<br>Handbook of Cannabis and Related Pathologies: Biology, Pharmacology, Diagnosis, and Treatment. 2017<br>893:904 doi:10.1016/B978-0-12-800756-3.00108-3	Scopus	Discard<br>Reasons:<br>Not of interest
92.	Cannabidiol and Neuroprotection: Evidence from Preclinical Studies<br>Schröder N, da Silva VK, Hallak JEC, Zuardi AW<br>Handbook of Cannabis and Related Pathologies: Biology, Pharmacology, Diagnosis, and Treatment. 2017<br>802-812 doi:10.1016/B978-0-12-800756-3.00095-8	Scopus	Discard<br>Reasons:<br>Not of interest
93.	Cannabidiol as a Potential Treatment for Febrile Infection-Related Epilepsy Syndrome (FIRES) in the Acute and Chronic Phases<br>Gofshteyn JS, Wilfong A, Devinsky O, Bluvstein J, Charuta J, Ciliberto MA, Laux L, Marsh ED<br>Journal of Child Neurology. 2017<br>32(1):35-40 doi:10.1177/0883073816669450	Scopus	Discard<br>Reasons:<br>Not of interest
94.	Cannabidiol as a Promising Strategy to Treat and Prevent Movement Disorders?<br>Peres FF, Lima AC, Hallak JEC, Crippa JA, Silva RH, Abilio VC.<br>Frontiers in Pharmacology 2018<br>9: 482 doi: 10.3389/fphar.2018.00482	Web of Science	Discard<br>Reasons:<br>Not of interest
95.	Cannabidiol as a therapy for ASIA syndrome? An editorial on a novel study<br>Katz D, Shoenfeld Y<br>Israel Medical Association Journal 2017<br>19(2):98-99	Scopus	Discard<br>Reasons:<br>Not of interest
96.	Cannabidiol as a Treatment for Mood Disorders: A Systematic Review<br>Pinto JV, Saraf G, Frysch C, Vigo D, Keramatian K, Chakrabarty T, Lam RW, Kauer-Sant'Anna M, Yatham LN<br>The Canadian Journal of Psychiatry 2019<br>65(4) doi:10.1177/0706743719895195	Web of Science	Discard<br>Reasons:<br>Not of interest
97.	Cannabidiol attenuates cardiac dysfunction, oxidative stress, fibrosis, and inflammatory and cell death signaling pathways in diabetic cardiomyopathy	Pubmed<br>Scopus<br>Web of Science	Discard<br>Reasons:<br>Not of interest

	Rajesh M, Mukhopadhyay P, BÃ¡tkai S, Patel V, Saito K, Matsumoto S, Kashiwaya Y, HorvÃ¡th B, Mukhopadhyay B, Becker L, HaskÃ³ G, Liaudet L, Wink DA, Veves A, Mechoulam R, Pacher P.<br>J Am Coll Cardiol. 2010<br>56(25):2115-25. doi: 10.1016/j.jacc.2010.07.033		
98.	Cannabidiol attenuates cisplatin-induced nephrotoxicity by decreasing oxidative/nitrosative stress, inflammation, and cell death<br>Pan H, Mukhopadhyay P, Rajesh M, Patel V, Mukhopadhyay B, Gao B, Haskó G, Pacher P.<br>J Pharmacol Exp Ther. 2009<br>328(3):708-14. doi: 10.1124/jpet.108.147181.	Pubmed<br>Scopus<br>Web of Science	Discard<br>Reasons:<br>Not of interest
99.	Cannabidiol attenuates deficits of visuospatial associative memory induced by Delta 9tetrahydrocannabinol.<br>Wright MJ, Vandewater SA, Taffe MA.<br>British Journal of Pharmacology 2013<br>170(7):1365-1373 doi:10.1111/bph.12199	Web of Science	Discard<br>Reasons:<br>Not of interest
100.	Cannabidiol Attenuates Experimental Autoimmune Encephalomyelitis Model of Multiple Sclerosis Through Induction of Myeloid-Derived Suppressor Cells<br>Elliott DM, Singh N, Nagarkatti M, Nagarkatti PS.<br>Front Immunol. 2018<br>9:1782. doi: 10.3389/fimmu.2018.01782	Pubmed<br>Scopus<br>Web of Science	Keep
101.	Cannabidiol attenuates high glucose-induced endothelial cell inflammatory response and barrier disruption<br>Rajesh M, Mukhopadhyay P, Bátkai S, Haskó G, Liaudet L, Drel VR, Obrosova IG, Pacher P.<br>Am J Physiol Heart Circ Physiol. 2007<br>293(1):H610-9. doi:10.1152/ajpheart.00236.2007.	Pubmed<br>Scopus<br>Web of Science	Discard<br>Reasons:<br>Not of interest
102.	Cannabidiol bioavailability after nasal and transdermal application: effect of permeation enhancers.<br>Paudel KS, Hammell DC, Agu RU, Valiveti S, Stinchcomb AL<br>Drug Development and Industrial Pharmacy 2010<br>36(9): 1088-1097 doi: 10.3109/03639041003657295	Web of science	Discard<br>Reasons:<br>Not of interest
103.	Cannabidiol causes endothelium-dependent vasorelaxation of human mesenteric arteries via CB1 activation<br>Stanley CP, Hind WH, Tufarelli C, O'Sullivan SE.<br>Cardiovasc Res. 2015<br>107(4):568-78. doi: 10.1093/cvr/cvv179.	Pubmed<br>Scopus<br>Web of Science	Discard<br>Reasons:<br>Not of interest

104.	Cannabidiol Elevates Mechanistic Target of Rapamycin Inhibitor Levels in Patients With Tuberous Sclerosis Complex<br>Ebrahimi-Fakhari D, Agricola KD, Tudor C, Krueger D, Franz DN<br>Pediatr Neurol. 2020<br>105:59-61. doi: 10.1016/j.pediatrneurol.2019.11.017	Pubmed<br>Scopus<br>Web of Science	Discard<br>Reasons:<br>Not of interest
105.	Cannabidiol for Dravet and Lennox-Gastaut syndromes: NICE guidance<br>Gaber T<br>Progress in Neurology and Psychiatry 2020<br>24(2): 4-6	Scopus	Discard<br>Reasons:<br>Not of interest
106.	Cannabidiol for Viral Diseases: Hype or Hope?<br>Mabou Tagne A, Pacchetti B, Sodergren M, Cosentino M, Marino F.<br>Cannabis Cannabinoid Res. 2020<br>5(2): 121-131 doi:10.1089/can.2019.0060	Pubmed<br>Scopus	Discard<br>Reasons:<br>Not of interest
107.	Cannabidiol improves lung function and inflammation in mice submitted to LPS-induced acute lung injury.<br>Ribeiro A, Almeida VI, Costola-de-Souza C, Ferraz-de-Paula V, Pinheiro ML, Vitoretti LB, Gimenes JA, Akamine AT, Crippa JA, Tavares-de-Lima W, Palermo-Neto J.<br>Immunopharmacology and Immunotoxicology 2015<br>37(1): 35-41 doi: 10.3109/08923973.2014.976794	Web of Science	Discard<br>Reasons:<br>Not of interest
108.	Cannabidiol in humans-The quest for therapeutic targets<br>Zhornitsky S,Potvin S<br>Pharmaceuticals 2012<br>5(5):529-552 doi:10.3390/ph5050529	PubMed<br>Scopus	Discard<br>Reasons:<br>Not of interest
109.	Cannabidiol in medicine: A review of its therapeutic potential in CNS disorders<br>Scuderi C, De Filippis D, Iuvone T, Blasio A, Steardo A, Esposito G<br>Phytotherapy Research 2009<br>23(5):597-602 doi:10.1002/ptr.2625	Scopus	Discard<br>Reasons:<br>Not of interest
110.	Cannabidiol Increases Survival and Promotes Rescue of Cognitive Function in a Murine Model of Cerebral Malaria<br>Campos AC, Brant F, Miranda AS, Machado FS, Teixeira AL<br>NEUROSCIENCE 2015<br>289: 166-180 doi: 10.1016/j.neuroscience.2014.12.051	Web of Science	Discard<br>Reasons:<br>Not of interest
111.	Cannabidiol induced apoptosis in human monocytes through mitochondrial permeability transition pore-mediated ROS production.<br>Wu HY, Huang CH, Lin YH, Wang CC, Jan TR.<br>Free Radic Biol Med. 2018<br>124: 311-318 doi: 10.1016/j.freeradbiomed.2018.06.023	Web of Science	Discard<br>Reasons:<br>Not of interest

112.	Cannabidiol inhibits paclitaxel-induced neuropathic pain through 5-HT1A receptors without diminishing nervous system function or chemotherapy efficacy<br>Ward SJ, McAllister SD, Kawamura R, Murase R, Neelakantan H, Walker EA<br>British Journal of Pharmacology 2014<br>171(3) SI: 636-645 DOI: 10.1111/bph.12439	Web of Science	Discard<br>Reasons:<br>Not of interest
113.	Cannabidiol inhibits pathogenic T cells, decreases spinal microglial activation and ameliorates multiple sclerosis-like disease in C57BL/6 mice<br>Kozela E, Lev N, Kaushansky N, Eilam R, Rimmerman N, Levy R, Ben-Nun A, Juknat A, Vogel Z.<br>Br J Pharmacol. 2011<br>163(7):1507-19. doi: 10.1111/j.1476-5381.2011.01379.x	Pubmed<br>Scopus<br>Web of Science	Keep
114.	Cannabidiol Limits T Cell-Mediated Chronic Autoimmune Myocarditis: Implications to Autoimmune Disorders and Organ Transplantation<br>Lee WS, Erdelyi K, Matyas C, Mukhopadhyay P, Varga ZV, Liaudet L, Haskú G, Čiháková D, Mechoulam R, Pacher P<br>Mol Med. 2016<br>22:136-146. doi: 10.2119/molmed.2016.00007	Pubmed<br>Scopus<br>Web of Science	Discard Reasons: Not of interest
115.	Cannabidiol Normalizes Caspase 3, Synaptophysin, and Mitochondrial Fission Protein DNM1L Expression Levels in Rats with Brain Iron Overload: Implications for Neuroprotection.<br>da Silva VK, de Freitas BS, Dornelles AD, Nery LR, Falavigna L, Ferreira RD, Bogo MR, Hallak JEC, Zuardi AW, Crippa JAS, Schroder N Molecular Neurobiology 2014<br>49(1): 222-233 doi: 10.1007/s12035-013-8514-7	Web of Science	Discard<br>Reasons:<br>Not of interest
116.	Cannabidiol prescription in clinical practice: An audit on the first 400 patients in New Zealand<br>Gulbransen G, Xu W, Arroll B<br>BJGP Open 2020<br>4(1): 8p	Scopus	Discard<br>Reasons:<br>Not of interest
117.	Cannabidiol prevents haloperidol-induced vacuos chewing movements and inflammatory changes in mice via PPAR gamma receptors.<br>Sonego AB, Prado DS, Vale GT, Sepulveda-Diaz JE, Cunha TM, Tirapelli CR, Del Bel EA, Raisman-Vozar, R, Guimaraes FS<br>Brain Behavior and Immunity 2018<br>74: 241-251 doi: 10.1016/j.bbi.2018.09.014	Web of Science	Discard<br>Reasons:<br>Not of interest
118.	Cannabidiol prevents LPS-induced microglial inflammation by inhibiting ROS/NF-kappa B-dependent signaling and glucose consumption.<br>dos-Santos-Pereira M; Guimaraes FS, Del-Bel E, Raisman-Vozari R, Michel PP	Web of Science	Discard<br>Reasons:<br>Not of interest

	Glia 2019<br>DOI: 10.1002/glia.23738		
119.	Cannabidiol protects oligodendrocyte progenitor cells from inflammation-induced apoptosis by attenuating endoplasmic reticulum stress<br>Mecha M, Torrao AS, Mestre L, Carrillo-Salinas FJ, Mechoulam R, Guaza C.<br>Cell Death Dis. 2012<br>3(6):e331. doi: 10.1038/cddis.2012.71	Pubmed<br>Scopus<br>Web of Science	Discard<br>Reasons:<br>Not of interest
120.	Cannabidiol provides long-lasting protection against the deleterious effects of inflammation in a viral model of multiple sclerosis: a role for A2A receptors<br>Mecha M, Feliú, Iñigo PM, Mestre L, Carrillo-Salinas FJ, Guaza C.<br>Neurobiol Dis. 2013<br>59:141-50. doi: 10.1016/j.nbd.2013.06.016.	Pubmed<br>Scopus<br>Web of Science	Keep
121.	Cannabidiol Regulates Gene Expression in Encephalitogenic T cells Using Histone Methylation and noncoding RNA during Experimental Autoimmune Encephalomyelitis<br>Yang X, Bam M, Nagarkatti PS, Nagarkatti M.<br>Sci Rep. 2019<br>9(1):15780. doi: 10.1038/s41598-019-52362-8	Pubmed<br>Scopus<br>Web of Science	Keep
122.	Cannabidiol to Improve Mobility in People with Multiple Sclerosis<br>Rudroff T, Sosnoff J.<br>Front Neurol. 2018<br>9:183. doi: 10.3389/fneur.2018.00183.	Pubmed<br>Scopus<br>Web of Science	Discard<br>Reasons:<br>Not of interest
123.	Cannabidiol, a Major Non-Psychotropic Cannabis Constituent Enhances Fracture Healing and Stimulates Lysyl Hydroxylase Activity in Osteoblasts.<br>Kogan NM, Melamed E, Wasserman E, Raphael B, Breuer A, Stok KS, Sondergaard R, Escudero AW, Baraghithy S, Attar-Namdar M, Friedlander-Barenboim S, Mathavan N, Isaksson H, Mechoulam R, Muller R, Bajayo A, Gabet Y, Bab I<br>Journal of Bone and Mineral Research 2015<br>30(10):1905-1913 DOI: 10.1002/jbmr.2513	Web of Science	Discard<br>Reasons:<br>Not of interest
124.	Cannabidiol, a non-psychoactive cannabinoid, leads to EGR2-dependent anergy in activated encephalitogenic T cells<br>Kozela E, Juknat A, Kaushansky N, Ben-Nun A, Coppola G, Vogel Z.<br>J Neuroinflammation. 2015<br>12:52. doi: 10.1186/s12974-015-0273-0	Pubmed<br>Scopus<br>Web of Science	Keep
125.	Cannabidiol, a nonpsychoactive Cannabis constituent, protects against myocardial ischemic reperfusion injury.<br>Durst R, Danenberg H, Gallily R, Mechoulam R, Meir K, Grad E, Beeri, R, Pugatsch T, Tarsish E, Lotan C	Web of Science	Discard<br>Reasons:<br>Not of interest

	Am J Physiol Heart Circ Physiol. 2007<br>293(6): H3602-H3607 doi: 10.1152/ajpheart.00098.2007		
126.	Cannabidiol, neuroprotection and neuropsychiatric disorders.<br>Campos AC, Fogaca MV, Sonego AB, Guimaraes FS<br>Pharmacological Research 2016<br>112, 119-127 doi: 10.1016/j.phrs.2016.01.033	Web of Science	Discard<br>Reasons:<br>Not of interest
127.	Cannabidiol: A Promising Drug for Neurodegenerative Disorders?<br>Iuvone T, Esposito G, De Filippis D, Scuderi C, Steardo L<br>CNS Neurosci Ther. 2009<br>15(1): 65-75 doi: 10.1111/j.1755-5949.2008.00065.x	Pubmed<br>Scopus<br>Web of Science	Discard<br>Reasons:<br>Not of interest
128.	Cannabidiol: an alternative therapeutic agent for oral mucositis?<br>Cuba LF, Salum FG, Cherubini K, Figueiredo MAZ<br>Journal of Clinical Pharmacy and Therapeutics 2017<br>42(3):245-250 doi:10.1111/jcpt.12504	Scopus	Discard<br>Reasons:<br>Not of interest
129.	Cannabidiol: Promise and Pitfalls<br>Welty TE, Luebke A, Gidal BE<br>Epilepsy Currents 2014<br>14(5):250-252 doi:10.5698/1535-7597-14.5.250	Pubmed<br>Scopus<br>Web of Science	Discard<br>Reasons:<br>Not of interest
130.	Cannabidiol: State of the art and new challenges for therapeutic applications<br>Pisanti S, Malfitano AM[2], Ciaglia E, Lamberti A, Ranieri R, Cuomo G, Abate M, Faggiana G , Proto MC , Fiore D, Laezza C, Bifulco M<br>Pharmacology and Therapeutics 2017<br>175:133-150 doi:10.1016/j.pharmthera.2017.02.041	Scopus<br>Web of Science	Discard<br>Reasons:<br>Not of interest
131.	Cannabidiol-Delta (9)-tetrahydrocannabinol interactions on acute pain and locomotor activity.<br>Britch SC, Wiley JL, Yu ZH, Clowers BH, Craft RM<br>Drug and Alcohol Dependence 2017<br>175: 187-197 doi: 10.1016/j.drugalcdep.2017.01.046	Web of Science	Discard<br>Reasons:<br>Not of interest
132.	Cannabidiol-induced myeloid-derived suppressor cells ameliorate experimental autoimmune encephalomyelitis through microRNA regulation.<br>Elliott DM, Nagarkatti P, Nagarkatti M<br>Journal of Neuroimmune Pharmacology 2015<br>10: S68-S69 Supplement: 2 doi: 10.1007/s11481-015-9596-y	Web of Science	Discard<br>Not of interest
133.	Cannabinoid 1 (CB1) receptor - pharmacology, role in pain and recent developments in emerging CB1 agonists<br>Talwar R, Potluri VK<br>CNS and Neurological Disorders - Drug Targets 2011	Scopus	Discard<br>Reasons:<br>Not of interest

	10(5):536-544 doi:10.2174/187152711796235005		
134.	Cannabinoid Buccal Spray for Chronic Non-Cancer or Neuropathic Pain: A Review of Clinical Effectiveness, Safety, and Guidelines [Internet].<br>No authors listed<br>Ottawa (ON): Canadian Agency for Drugs and Technologies in Health 2016	Pubmed	Discard<br>Reasons:<br>Not of interest
135.	Cannabinoid CB 2 receptors in human brain inflammation<br>Benito C,Tolón RM, Pazos MR,Núñez E, Castillo AI, Romero J<br>British Journal of Pharmacology 2008<br>153(2):277-285 doi:10.1038/sj.bjp.0707505	Scopus	Discard<br>Reasons:<br>Not of interest
136.	Cannabinoid CB1 and CB2 receptor ligand specificity and the development of CB2-selective agonists<br>Ashton JC, Wright JL, McPartland JM,Tyndall JDA<br>Current Medicinal Chemistry 2008<br>15(14):1428-1443 doi:10.2174/092986708784567716	Scopus	Discard<br>Reasons:<br>Not of interest
137.	Cannabinoid control of neuroinflammation related to multiple sclerosis.<br>Baker D, Jackson SJ, Pryce G<br>British Journal of Pharmacology 2007<br>152(5): 649-654 doi: 10.1038/sj.bjp.0707458	Scopus<br>Web of Science	Discard<br>Reasons:<br>Review
138.	Cannabinoid delivery systems for pain and inflammation treatment<br>Bruni N, Della Peppa C, Oliaro-Bosso S, Pessione E, Gastaldi D, Dosio F<br>Molecules 2018<br>23(10):2493 doi:10.3390/molecules23102478	Pubmed<br>Scopus<br>Web of Science	Discard<br>Reasons:<br>Not of interest
139.	Cannabinoid drugs and enhancement of endocannabinoid responses: Strategies for a wide array of disease states<br>Karanian DA, Bahr BA<br>Current Molecular Medicine 2006<br>6(6):677-684 doi:10.2174/156652406778194991	Scopus	Discard<br>Reasons:<br>Not of interest
140.	Cannabinoid drugs for neurological diseases: What is behind? [Fármacos cannabinoides para las enfermedades neurológicas: ¿qué hay detrás?<br>Fernández-Ruiz J<br>Revista de Neurologia 2012<br>54(10):613-628	Scopus	Discard<br>Reasons:<br>Not of interest
141.	Cannabinoid modulation of neuroinflammatory disorders<br>Saito VM, Rezende RM, Teixeira AL<br>Current Neuropharmacology 2012<br>10(2):159-166 doi:10.2174/157015912800604515	Scopus	Discard<br>Reasons:<br>Not of interest
142.	Cannabinoid pharmacology in cancer research: A new hope for cancer patients?	Scopus	Discard

	Javid FA, Phillips RM, Afshinjavid S, Verde R, Ligresti A<br>European Journal of Pharmacology 2016<br>775(15):1-14 doi:10.1016/j.ejphar.2016.02.010		Reasons:<br>Not of interest
143.	Cannabinoid pharmacology: The first 66 years<br>Pertwee RG<br>British Journal of Pharmacology 2006<br>147 SUPPL. 1 S163-S171 doi:10.1038/sj.bjp.0706406	Scopus	Discard<br>Reasons:<br>Not of interest
144.	Cannabinoid receptor 2: Potential role in immunomodulation and neuroinflammation<br>Rom S, Persidsky Y<br>Journal of Neuroimmune Pharmacology 2013<br>8(3):608-620 doi:10.1007/s11481-013-9445-9	Scopus	Discard<br>Reasons:<br>Not of interest
145.	Cannabinoid receptors as new targets of antifibrosing strategies during chronic liver diseases<br>Mallat A,Teixeira-Clerc F, Deveaux V, Lotersztajn S<br>Expert Opinion on Therapeutic Targets 2007<br>11(3):403-409 doi:10.1517/14728222.11.3.403	Scopus	Discard<br>Reasons:<br>Not of interest
146.	Cannabinoid signaling in glial cells in health and disease<br>Witting A, Stella N<br>Current Neuropharmacology 2002<br>2(1):115-124 doi:10.2174/1570159043476855	Scopus	Discard<br>Reasons:<br>Not of interest
147.	Cannabinoid therapeutics: High hopes for the future<br>Hensen B<br>Drug Discovery Today 2005<br>10(7):459-462 doi:10.1016/S1359-6446(05)03417-3	Scopus	Discard<br>Reasons:<br>Not of interest
148.	Cannabinoid therapy in practice [Cannabinoidtherapie in der Praxis]<br>Rasche T, Emmert D, Stieber C, Mücke M, Conrad R.<br>Urologe 2018<br>57(5):558-562 doi:10.1007/s00120-018-0636-0	Pubmed<br>Scopus<br>Web of Science	Discard<br>Reasons:<br>Not of interest
149.	Cannabinoid-based drugs as anti-inflammatory therapeutics<br>Klein TW<br>Nature Reviews Immunology ,2005<br>5(5):400-411 doi:10.1038/nri1602	Scopus	Discard<br>Reasons:<br>Not of interest
150.	Cannabinoid-based medicines for neurological disorders - Clinical evidence<br>Wright S<br>Molecular Neurobiology 2007<br>36(1):129-136 doi:10.1007/s12035-007-0003-4	Scopus<br>Web of Science	Discard<br>Reasons:<br>Not of interest
151.	Cannabinoid-induced apoptosis in immune cells as a pathway to immunosuppression	Scopus	Discard

	Rieder SA, Chauhan A, Singh U, Nagarkatti M, Ngarkatti P<br>Immunobiology 2010<br>215(8):598-605 doi:10.1016/j.imbio.2009.04.001	Web of Science	Reasons:<br>Not of interest
152.	Cannabinoid-induced effects on the nociceptive system: a neurophysiological study in patients with secondary progressive multiple sclerosis<br>Conte A, Bettolo CM, Onesti E, Frasca V, Iacovelli E, Gilio F, Giacomelli E, Gabriele M, Aragona M, Tomassini V, Pantano P, Pozzilli C, Inghilleri M.<br>Eur J Pain. 2009<br>13(5):472-7. doi: 10.1016/j.ejpain.2008.05.014.	Pubmed<br>Scopus<br>Web of Science	Discard<br>Reasons:<br>Not of interest
153.	Cannabinoids against pain. Efficacy and strategies to reduce psychoactivity: A clinical perspective<br>Expert Opinion on Investigational Drugs 2009<br>18(2):125-133 doi:10.1517/13543780802691951	Scopus<br>Web of Science	Discard<br>Reasons:<br>Not of interest
154.	Cannabinoids and cancer: Pros and cons of an antitumour strategy<br>Bifulco M, Laezza C, Pisanti S, Gazzerro P<br>British Journal of Pharmacology 2006<br>148(2):123-135 doi:10.1038/sj.bjp.0706632	Scopus	Discard<br>Reasons:<br>Not of interest
155.	Cannabinoids and hallucinogens for headache<br>McGenney BE<br>Headache 2013<br>53(3):447-458 doi:10.1111/head.12025	Scopus	Discard<br>Reasons:<br>Not of interest
156.	Cannabinoids and neurodegenerative diseases<br>Romero J, Orgado JM<br>CNS and Neurological Disorders - Drug Targets 2009<br>8(6):440-450 doi:10.2174/187152709789824589	Scopus	Discard<br>Reasons:<br>Not of interest
157.	Cannabinoids and neuroinflammation<br>Walter L, Stella N<br>British Journal of Pharmacology 2004<br>141(5):775-785 doi:10.1038/sj.bjp.0705667	Scopus	Discard<br>Reasons:<br>Not of interest
158.	Cannabinoids and neuroprotection in CNS inflammatory disease<br>Jackson SJ, Diemel LT, Pryce G, Baker D<br>Journal of the Neurological Sciences 2005<br>233(1-2):21-25 doi:10.1016/j.jns.2005.03.002	Scopus	Discard<br>Reasons:<br>Not of interest
159.	Cannabinoids and neuroprotection in motor-related disorders<br>De Lago E, Fernández-Ruiz J<br>CNS and Neurological Disorders - Drug Targets 2007<br>6(6):377-387 doi:10.2174/187152707783399210	Scopus	Discard<br>Reasons:<br>Not of interest

160.	Cannabinoids and the brain<br>Parker LA<br>Cannabinoids and the Brain 2017<br>1(237):5	Scopus	Discard<br>Reasons:<br>Not of interest
161.	Cannabinoids and the expanded endocannabinoid system in neurological disorders<br>Cristino L, Bisogno T, Di Marzo V.<br>Nat Rev Neurol. 2020<br>6(1):9-29. doi: 10.1038/s41582-019-0284-z.	Pubmed<br>Scopus<br>Web of Science	Discard<br>Reasons:<br>Not of interest
162.	Cannabinoids and the immune system: An overview<br>Tanasescu R, Constantinescu CS<br>Immunobiology 2010<br>215(8):588-597 doi:10.1016/j.imbio.2009.12.005	Scopus	Discard<br>Reasons:<br>Not of interest
163.	Cannabinoids and the immune system: Potential for the treatment of inflammatory diseases?<br>Croxford JL, Yamamura T<br>Journal of Neuroimmunology 2005<br>166(1-2):3-18 doi:10.1016/j.jneuroim.2005.04.023	Scopus	Discard<br>Reasons:<br>Not of interest
164.	Cannabinoids as modulators of cell death: Clinical applications and future directions<br>Fonseca BM, Teixeira NA, Correia-da-Silva G<br>Reviews of Physiology, Biochemistry and Pharmacology 2017<br>173:63-88 doi:10.1007/112_2017_3	Scopus	Discard<br>Reasons:<br>Not of interest
165.	Cannabinoids as novel anti-inflammatory drugs<br>Nagarkatti P, Pandey R, Rieder SA, Hedge VL, Nagarkatti M<br>Future Medicinal Chemistry 2009<br>1(7):1333-1349 doi:10.4155/fmc.09.93	Scopus	Discard<br>Reasons:<br>Not of interest
166.	Cannabinoids as pharmacotherapies for neuropathic pain: from the bench to the bedside.<br>Rahn EJ, Hohmann AG<br>Neurotherapeutics. 2009<br>6(4):713-37. doi: 10.1016/j.nurt.2009.08.002	Pubmed<br>Scopus<br>Web of Science	Discard<br>Reasons:<br>Not of interest
167.	Cannabinoids as therapeutic agents for ablating neuroinflammatory disease<br>Cabral GA, Griffin-Thomas L<br>Endocrine, Metabolic and Immune Disorders - Drug Targets 2008<br>8(3):159-172 doi:10.2174/187153008785700118	Scopus	Discard<br>Reasons:<br>Not of interest
168.	Cannabinoids decrease the Th17 inflammatory autoimmune phenotype<br>Kozela E, Juknat A, Kaushaansky N, Rimmerman N, Ben-Nun A, Vogel Z<br>Journal of Neuroimmune Pharmacology 2013<br>8(5):1265-1276 doi:10.1007/s11481-013-9493-1	Pubmed<br>Scopus<br>Web of Science	Keep

169.	Cannabinoids for Epilepsy - Real Data, at Last<br>Berkovic SF<br>New England Journal of Medicine 2017<br>376(21):2075-2076 doi:10.1056/NEJMe1702205	Scopus	Discard<br>Reasons:<br>Not of interest
170.	Cannabinoids for medical use: A systematic review and meta-analysis<br>Whiting PF, Wolff RF, Deshpande S, Di Nisio M, Duffy S, Hernandez AV, Keurentjes JC, Lang S, Misso K, Ryder S, Schmidlkofe S, Westwood M, Kleijnen J<br>JAMA - Journal of the American Medical Association 2015<br>313(24):2456-2473 doi:10.1001/jama.2015.6358	Scopus	Discard<br>Reasons:<br>Not of interest
171.	Cannabinoids for pediatric epilepsy? Up in smoke or real science?<br>Filloux FM<br>Translational Pediatrics 2015<br>4(4):271-282 doi: 10.3978/j.issn.2224-4336.2015.10.03	Web of Science	Discard<br>Reasons:<br>Not of interest
172.	Cannabinoids for spasticity due to multiple sclerosis or paraplegia: A systematic review and meta-analysis of randomized clinical trials<br>da Rovare PV, Magalhães GPA, Jardini GDA, Beraldo ML, Gameiro MO, Agarwal A, Luvizutto GJ, Paula-Ramos L, Camargo SEA, Dias de Oliveira L, Bazan R, El Dib R<br>Complementary Therapies in Medicine 2017<br>34:170-185 doi:10.1016/j.ctim.2017.08.010	Scopus	Discard<br>Reasons:<br>Not of interest
173.	Cannabinoids for symptomatic therapy of multiple sclerosis.<br>Husseini L, Leussink VI, Warnke C, Hartung HP, Kieseier BC.<br>Nervenarzt. 2012<br>83(6):695-704. doi: 10.1007/s00115-011-3401-9.	Pubmed<br>Scopus<br>Web of Science	Discard<br>Reasons:<br>Not of interest
174.	Cannabinoids for the pharmaceutical industry.<br>Stott CG, Guy GW<br>Euphytica 2004<br>140(1-2):83-93 doi: 10.1007/s10681-004-4757-8	Web of Science	Discard<br>Reasons:<br>Not of interest
175.	Cannabinoids for the treatment of mental disorders and symptoms of mental disorders: a systematic review and meta-analysis<br>Black N, Stockings E, Campbell G, Tran LT, Zagic D, Hall WD, Farrell M, Degenhardt L.<br>Lancet Psychiatry. 2019<br>6(12):995-1010. doi: 10.1016/S2215-0366(19)30401-8.	Scopus<br>Web of Science	Discard<br>Reasons:<br>Not of interest
176.	Cannabinoids for the treatment of neuropathic pain: Clinical evidence.<br>Ashton JC, Milligan ED<br>Curr Opin Investig Drugs 2008<br>9(1): 65-75	Web of Science	Discard<br>Reasons:<br>Not of interest

177.	Cannabinoids for the treatment of spasticity.<br>Nielsen S, Murnion B, Campbell G,Young H, Hall W<br>DEVELOPMENTAL MEDICINE AND CHILD NEUROLOGY 2019<br>61(6):631 doi: 10.1111/dmcn.14165	Web of Science	Discard<br>Reasons:<br>Not of interest
178.	Cannabinoids for Treatment of MS Symptoms: State of the Evidence<br>Rice J, Cameron MH<br>Current Neurology and Neuroscience Reports 2017<br>18(8) doi:10.1007/s11910-018-0859-x	Pubmed<br>Scopus<br>Web of Science	Discard<br>Reasons:<br>Not of interest
179.	Cannabinoids for treatment of spasticity and other symptoms related to multiple sclerosis (CAMS study): multicentre randomised placebo-controlled trial<br>Zajicek J, Fox P, Sanders H, Wright D, Vickery J, Nunn A, Thompson A, UK MS Research Group<br>Lancet. 2003<br>362(9395):1517-26	Pubmed	Discard<br>Reasons:<br>Not of interest
180.	Cannabinoids in (cancer) pain [Cannabinoide bei (Tumor-)Schmerzen]<br>Karst M<br>Deutsche Zeitschrift fur Onkologie 2011<br>41:150-153 doi:10.1055/s-0030-1257697	Scopus	Discard<br>Reasons:<br>Not of interest
181.	Cannabinoids in anaesthesia and pain therapy<br>Azad S, Rammes G<br>Current Opinion in Anaesthesiology 2005<br>18(4):424-427 doi:10.1097/01.aco.0000174959.05383.9c	Scopus	Discard<br>Reasons:<br>Not of interest
182.	Cannabinoids in bipolar affective disorder: a review and discussion of their therapeutic potential.<br>Ashton CH, Moore PB, Gallagher P, Young AH<br>JOURNAL OF PSYCHOPHARMACOLOGY 2005<br>19(3): 293-300 DOI: 10.1177/0269881105051541	Web of Science	Discard<br>Reasons:<br>Not of interest
183.	Cannabinoids in chronic pain and palliative care [Uso de canabinóides na dor crônica e em cuidados paliativos]<br>Bonfá L, de Oliveira Vinagre C, de Figuiredo NV<br>Revista Brasileira de Anestesiologia 2008<br>58(3):267-279 doi:10.1590/S0034-70942008000300010	Scopus	Discard<br>Reasons:<br>Not of interest
184.	Cannabinoids in clinical practice<br>Williamson EM, Evans FJ<br>Drugs 2000<br>60(6):1303-1314 doi:10.2165/00003495-200060060-00005	Pubmed<br>Scopus<br>Web of Science	Discard<br>Reasons:<br>Not of interest
185.	Cannabinoids in clinical practice	Scopus	Discard

	Kostoudi S, Koutousi C, Mironidou-Tzouveleki M<br>Epitheorese Klinikes Farmakologias kai Farmakokinetikes 2005<br>23(3):265-272		Reasons:<br>Not of interest
186.	Cannabinoids in health and disease<br>Kogan NM, Mechoulam R<br>Dialogues in Clinical Neuroscience 2007<br>9(4):413-430	Scopus	Discard<br>Reasons:<br>Not of interest
187.	Cannabinoids in medicine: A review of their therapeutic potential<br>Amar MB<br>Journal of Ethnopharmacology 2006<br>105(1-2):1-25 doi:10.1016/j.jep.2006.02.001	Scopus	Discard<br>Reasons:<br>Not of interest
188.	Cannabinoids in MS - Are we any closer to knowing how best to use them?<br>Bowling A<br>Multiple Sclerosis 2006<br>12(5):523-525 doi:10.1177/1352458506072514	Scopus	Discard<br>Reasons:<br>Not of interest
189.	Cannabinoids in multiple sclerosis: A neurophysiological analysis<br>Vecchio D, Varrasi C, Virgilio E, Spagarino A, Naldi P, Cantello R.<br>Acta Neurol Scand. 2020<br>00:1–6 doi: 10.1111/ane.13313	Pubmed<br>Scopus	Discard<br>Reasons:<br>Not of interest
190.	Cannabinoids in Neurodegenerative Disorders and Stroke/Brain Trauma: From Preclinical Models to Clinical Applications<br>Fernández-Ruiz J, Moro MA, Martínez-Orgado J<br>Neurotherapeutics 2015<br>12(4):793-806 doi:10.1007/s13311-015-0381-7	Scopus	Discard<br>Reasons:<br>Not of interest
191.	Cannabinoids in neurology - Brazilian academy of neurology [Canabinoides e seu uso em neurologia - Academia brasileira de neurologia]<br>Brucki SM, Frota NA, Schestatsky P, Souza AH, Carvalho VN, Manreza ML, Mendes MF, Comini-Frota E, Vasconcelos C, Tumas V, Ferraz HB, Barbosa E, Jurno ME<br>Arquivos de Neuro-Psiquiatria 2015<br>73(4):371-374 doi:10.1590/0004-282X20150041	Pubmed<br>Scopus<br>Web of Science	Discard<br>Reasons:<br>Not of interest
192.	Cannabinoids in pain medicine [Cannabinoide in der schmerztherapie]<br>Briner A, Schneider T<br>Therapeutische Umschau 2017<br>74(5):261-266 doi:10.1024/0040-5930/a000913	Scopus<br>Web of Science	Discard<br>Reasons:<br>Not of interest
193.	Cannabinoids in Palliative Medicine<br>Strouse TB<br>Journal of Palliative Medicine 2017	Scopus	Discard<br>Reasons:<br>Not of interest

	20(7):692-694 doi:10.1089/jpm.2017.0197		
194.	Cannabinoids in the management of difficult to treat pain<br>Russo EB<br>Therapeutics and Clinical Risk Management 2008<br>4(1):245-259	Pubmed<br>Scopus	Discard<br>Reasons:<br>Not of interest
195.	Cannabinoids in the treatment of epilepsy<br>Friedman D, Devinsky O<br>New England Journal of Medicine 2015<br>373(11):1048-1058 doi:10.1056/NEJMra1407304	Scopus	Discard<br>Reasons:<br>Not of interest
196.	Cannabinoids in the treatment of pain and spasticity in multiple sclerosis<br>Smith PF<br>Current Opinion in Investigational Drugs 2002<br>3(6):859-864	Scopus	Discard<br>Reasons:<br>Not of interest
197.	Cannabinoids Part I - Legal policies and physiological effects<br>Kerwin J<br>Drug Testing and Analysis 2013<br>5(1):20-21 doi:10.1002/dta.1440	Scopus	Discard<br>Reasons:<br>Not of interest
198.	Cannabinoids quantification in medicinal cannabis extracts by high performance liquid chromatography [Quantificaçao de canabinoides em extratos medicinais de cannabis por cromatografia líquida de alta eficiência]<br>Carvalhoa VM, Aguiara AFL, Barattob LC, Souzaa FLC, Rocha ED<br>Quím. Nova 2020<br>43(1) doi: 10.21577/0100-4042.20170457	Scopus<br>Web of Science	Discard<br>Reasons:<br>Not of interest
199.	Cannabinoids therapeutic use: what is our current understanding following the introduction of THC, THC:CBD oromucosal spray and others?<br>Maccarrone M, Maldonado R, Casas M, Henze T, Centonze D.<br>Expert Rev Clin Pharmacol. 2017<br>10(4):443-455. doi: 10.1080/17512433.2017.1292849.	Pubmed<br>Scopus<br>Web of Science	Discard<br>Reasons:<br>Not of interest
200.	Cannabinoids, endocannabinoids, and related analogs in inflammation<br>Burstein S, Zurier RB<br>AAPS Journal 2009<br>11(1):109-119 doi:10.1208/s12248-009-9084-5	Scopus	Discard<br>Reasons:<br>Not of interest
201.	Cannabinoids, inflammation, and fibrosis.<br>Zurier RB, Burstein SH<br>Faseb Journal 2016<br>30(11): 3682-3689 doi: 10.1096/fj.201600646R	Web of Science	Discard<br>Reasons:<br>Not of interest
202.	Cannabinoids, multiple sclerosis and neuroprotection	Scopus	Discard

	De Lago E, Gómez-Ruiz M, Moreno-Martet M, Fernández-Ruiz J<br>Expert Review of Clinical Pharmacology 2009<br>2(6):645-660 doi:10.1586/ecp.09.42		Reasons:<br>Not of interest
203.	Cannabinoids<br>Howard P, Twycross R, Shuster J, Mihalyo M, Wilcock A<br>Journal of Pain and Symptom Management 2013<br>46(1):142-149 doi:10.1016/j.jpainsymman.2013.05.002	Scopus<br>Web of Science	Discard<br>Reasons:<br>Not of interest
204.	Cannabinoids: Between neuroprotection and neurotoxicity<br>Sarne Y, Mechoulam R<br>Current Drug Targets: CNS and Neurological Disorders 2005<br>4(6):677-684 doi:10.2174/156800705774933005	Scopus	Discard<br>Reasons:<br>Not of interest
205.	Cannabinoids: From plant to patient<br>Barnes F<br>Pharmaceutical Journal 2001<br>266:7143	Scopus	Discard<br>Reasons:<br>Not of interest
206.	Cannabinoids: Mechanisms and therapeutic applications in the CNS<br>Drysdale AJ, Platt B<br>Current Medicinal Chemistry 2003<br>10(24):2719-2732 doi:10.2174/0929867033456387	Scopus	Discard<br>Reasons:<br>Not of interest
207.	Cannabinoids: Medical implications<br>Schrot RJ, Hubbard JR<br>Annals of Medicine 2016<br>48(3):128-141 doi:10.3109/07853890.2016.1145794	Pubmed<br>Scopus<br>Web of Science	Discard<br>Reasons:<br>Not of interest
208.	Cannabinoids: novel medicines for the treatment of Huntington's disease<br>Sagredo O, Pazos MR, Valdeolivas S, Fernandez-Ruiz J.<br>Recent Pat CNS Drug Discov. 2012<br>7(1):41-8	Pubmed<br>Scopus	Discard<br>Reasons:<br>Not of interest
209.	Cannabinoids: Occurrence and medicinal chemistry<br>Appendino G, Chianese G, Taglialatela-Scafato O<br>Current Medicinal Chemistry 2011<br>18(7):1085-1099 doi:10.2174/092986711794940888	Scopus<br>Web of Science	Discard<br>Reasons:<br>Not of interest
210.	Cannabinoids: Potential targets for bladder dysfunction<br>Ruggieri MR<br>Handbook of Experimental Pharmacology 2011<br>202:425-451 doi:10.1007/978-3-642-16499-6_20	Scopus	Discard<br>Reasons:<br>Not of interest
211.	Cannabinoids: Their role in pain and palliation<br>McCarberg BH	Scopus	Discard<br>Reasons:

	Journal of Pain and Palliative Care Pharmacotherapy 2007<br>21(3)19-28 doi:10.1300/J354v21n03_04		Not of interest
212.	Cannabis - medicinal use. [Cannabis: Nicht nur Suchtmittel, sondern auch Medikament.]<br>Deutsche medizinische Wochenschrift (1946) 2014<br>139(3):74-75 doi:10.1055/s-0033-1353961	Pubmed<br>Scopus	Discard<br>Reasons:<br>Not of interest
213.	Cannabis - The therapeutic potential<br>Gray C<br>Pharmaceutical Journal 1995<br>254(6843):771-773	Scopus	Discard<br>Reasons:<br>Not of interest
214.	Cannabis and analgesia [Cannabis et analgésie]<br>Benyamina A, Blecha L<br>Douleur et Analgesie 2012<br>25(2):78-82 doi: 10.1007/s11724-012-0294-8	Scopus	Discard<br>Reasons:<br>Not of interest
215.	Cannabis and cancer: Reality or pipe dream?<br>Catchart P, de Giorgio A, Stebbing J<br>The Lancet Oncology 2015<br>16(13):1291-1292 doi:10.1016/S1470-2045(15)00302-2	Scopus	Discard<br>Reasons:<br>Not of interest
216.	Cannabis and Cannabinoids for MS Symptoms<br>Goss AL, Cameron M<br>Annals of Neurology 2020<br>87(6) doi: 10.1002/ana.25750	Scopus	Discard<br>Reasons:<br>Not of interest
217.	Cannabis and cannabinoids for the treatment of people with chronic noncancer pain conditions: A systematic review and meta-analysis of controlled and observational studies<br>Stockings E,Campbell G, Hall WD, Nielsen S, Zagic D, Rahman R, Murnion B, Farrell M, Weier M, Degenhardt L<br>Pain 2018<br>159(10):1932-1954 doi:10.1097/j.pain.0000000000001293	Scopus	Discard<br>Reasons:<br>Not of interest
218.	Cannabis and cannabinoids in medicine [Cannabis et cannabinoïdes en médecine]<br>Girault L<br>Actualites Pharmaceutiques 2009<br>48(486):19-25 doi:10.1016/S0515-3700(09)70463-X	Scopus	Discard<br>Reasons:<br>Not of interest
219.	Cannabis and cannabinoids in the treatment of neuropathic pain [Cannabis y cannabinoides en el tratamiento del dolor neuropático]<br>Dolor 2005<br>20(4):213-216	Scopus	Discard<br>Reasons:<br>Not of interest
220.	Cannabis and cannabinoids<br>No authors listed	Scopus	Discard<br>Reasons:

	JAMA - Journal of the American Medical Association 2016<br>316(22):2424-2425 doi:10.1001/jama.2016.11772		Not of interest
221.	Cannabis and cannabinoids.<br>[No authors listed]<br>Med Lett Drugs Ther. 2019<br>61(1585):179-182. No abstract available.	Pubmed<br>Scopus	Discard<br>Reasons:<br>Not of interest
222.	Cannabis and cannabinoids-easier access, hype and disappointment: What has been confirmed in therapy?<br>Rasche T, Emmert D, Stieber C, Conrad R, Mücke M.<br>Internist (Berl). 2019<br>60(3):309-314. doi: 10.1007/s00108-019-0556-0.	Pubmed<br>Scopus<br>Web of Science	Discard<br>Reasons:<br>Not of interest
223.	Cannabis and Cannabis based medicine extracts: Additional results<br>Russo E<br>Journal of Cannabis Therapeutics 2003<br>3(4):153-161 doi:10.1300/J175v03n04_03	Scopus	Discard<br>Reasons:<br>Not of interest
224.	Cannabis and endocannabinoid modulators: Therapeutic promises and challenges<br>Grant I, Cahn BR<br>Clinical Neuroscience Research 2005<br>5(2-4):185-199 doi:10.1016/j.cnr.2005.08.015	Scopus	Discard<br>Reasons:<br>Not of interest
225.	Cannabis and endocannabinoid signaling in Epilepsy<br>Katona I<br>Handbook of Experimental Pharmacology 2015<br>231:285-316 doi:10.1007/978-3-319-20825-1_10	Scopus	Discard<br>Reasons:<br>Not of interest
226.	Cannabis and endocannabinoids: 'The old man and the teenagers'<br>Lambert DM<br>Chemistry and Biodiversity 2007<br>4(8):1609-1613 doi:10.1002/cbdv.200790143	Scopus	Discard<br>Reasons:<br>Not of interest
227.	Cannabis and intractable chronic pain: an explorative retrospective analysis of Italian cohort of 614 patients<br>Fanelli G, De Carolis G, Leonardi C, Longobardi A, Sarli E, Allegri M, Schatman ME.<br>J Pain Res. 2017<br>10:1217-1224. doi: 10.2147/JPR.S132814.	Pubmed<br>Scopus<br>Web of Science	Discard<br>Reasons:<br>Not of interest
228.	Cannabis and multiple sclerosis<br>Fragoso YD, Carra A, Macias MA.<br>Expert Rev Neurother. 2020<br>doi: 10.1080/14737175.2020.1776610	Pubmed<br>Scopus<br>Web of Science	Discard<br>Reasons:<br>Not of interest
229.	Cannabis and multiple sclerosis	Pubmed	Discard

	Ingram G, Pearson OR.<br>Pract Neurol. 2019<br>19(4):310-315. doi: 10.1136/practneurol-2018-002137.	Scopus	Reasons:<br>Not of interest
230.	Cannabis and Neuropsychiatric Disorders: An Updated Review<br>Chayasirisobhon S.<br>Acta Neurol Taiwan. 2019<br>28(2):27-39	Pubmed<br>Scopus	Discard<br>Reasons:<br>Not of interest
231.	Cannabis and pain: A review<br>Journal of Pain Management 2016<br>9(4):395-413	Scopus	Discard<br>Reasons:<br>Not of interest
232.	Cannabis and Pediatric Inflammatory Bowel Disease: Change Blossoms a Mile High<br>Hoffenberg EJ, Newman H, Collins C, Tarbell S, Leinwand K<br>Journal of Pediatric Gastroenterology and Nutrition 2017<br>64(2):265-271 doi:10.1097/MPG.0000000000001393	Scopus	Discard<br>Reasons:<br>Not of interest
233.	Cannabis as a possible treatment for spasticity in multiple sclerosis [Kanabis kao mogući tretman u lečenju spastičnosti kod multiple skleroze]<br>Vesic K, Dejanovic SD, Borovcanin M, Samardzic J, Toncev G<br>Serbian Journal of Experimental and Clinical Research 2016<br>17(1):61-66 doi:10.1515/SJECR-2015-0047	Scopus	Discard<br>Reasons:<br>Not of interest
234.	Cannabis as a therapeutic agent<br>Notcutt W<br>CPD Anaesthesia 2000<br>2(3):134-139	Scopus	Discard<br>Reasons:<br>Not of interest
235.	Cannabis based drug is licensed for spasticity in patients with MS<br>Kmietowicz Z.<br>BMJ. 2010<br>340:c3363. doi: 10.1136/bmj.c3363. No abstract available.	Pubmed<br>Scopus	Discard<br>Reasons:<br>Not of interest
236.	Cannabis derivatives therapy for a seronegative stiff-person syndrome: a case report.<br>Vicente-Valor MI, Garcia-Llopis P, Mejia Andujar L, Antonino de la Camara G, GarcÃ-a del Busto N, Lopez Tinoco MJ, Quintana Vergara B, Peiro Vilaplana C, Dominguez Moran JA, SÃ¡nchez Alcaraz A.<br>J Clin Pharm Ther. 2013<br>38(1):71-3. doi: 10.1111/j.1365-2710.2012.01365.x.	Pubmed<br>Scopus<br>Web of Science	Discard<br>Reasons:<br>Not of interest
237.	Cannabis drugs: "Narcotic" on prescription? [Cannabismedikamente: "Rauschgift" auf rezept?]<br>Gross H<br>MMW-Fortschritte der Medizin 2010	Pubmed<br>Scopus	Discard<br>Reasons:<br>Not of interest

	152(45):14-15+16		
238.	Cannabis for Chronic Pain: Challenges and Considerations<br>Romero-Sandoval EA, Fincham JE, Kolano AL, Sharpe BN, Alvarado-Vázquez PA<br>Pharmacotherapy 2018<br>38(6):651-662 doi: 10.1002/phar.2115	Scopus	Discard<br>Reasons:<br>Not of interest
239.	Cannabis for inflammatory bowel disease<br>Naftali T, Mechulam R, Lev LB, Konikoff FM<br>Digestive Diseases ,2014<br>32(4):468-474 doi:10.1159/000358155	Scopus	Discard<br>Reasons:<br>Not of interest
240.	Cannabis for neuropathic pain in multiple sclerosis-high expectations, poor data<br>Rudroff T<br>Frontiers in Pharmacology 2019<br>10:1239 doi:10.3389/fphar.2019.01239	Scopus	Discard<br>Reasons:<br>Not of interest
241.	Cannabis for Pain and Headaches: Primer.<br>Kim PS, Fishman MA.<br>Current Pain and Headache Reports 2017<br>21(4):19 DOI: 10.1007/s11916-017-0619-7	Web of Science	Discard<br>Reasons:<br>Not of interest
242.	Cannabis in movement disorders.<br>Muller-Vahl KR, Kolbe H, Schneider U, Emrich HM<br>Forschende Komplementarmedizin 1999<br>6 Suppl 3:23-27 doi: 10.1159/000057153 Supplement: 3	Web of Science	Discard<br>Reasons:<br>Not of interest
243.	Cannabis in neurology - A potted review<br>Hosking R, Zajicek J<br>Nature Reviews Neurology 2014<br>10(8):429-430 doi:10.1038/nrneurol.2014.122	Scopus	Discard<br>Reasons:<br>Not of interest
244.	Cannabis in New Zealand: Smoking gun or medicalised smokescreen?<br>Newton-Howes G, McBride S<br>New Zealand Medical Journal 2016<br>129(1433):6862	Scopus	Discard<br>Reasons:<br>Not of interest
245.	Cannabis medicines in Norway--the basis for approval<br>Gramstad L, Sagabråten AS.<br>Tidsskr Nor Laegeforen. 2012<br>132(23-24):2598-9. doi: 10.4045/tidsskr.12.1250. Norwegian. No abstract available.	Pubmed<br>Scopus	Discard<br>Reasons:<br>Not of interest
246.	Cannabis oral spray effective against pain in multiple sclerosis? [Cannabis-mundspray wirksam gegen schmerzen bei multipler sklerose?]<br>Zeitschrift fur Phytotherapie 2006<br>27(3):123-124 doi:10.1055/s-2006-951380	Scopus	Discard<br>Reasons:<br>Not of interest

247.	Cannabis sativa in the light of scientific research<br>Szulakowska A, Milnerowicz H<br>Advances in Clinical and Experimental Medicine 2007<br>16(6):807-815	Scopus	Discard<br>Reasons:<br>Not of interest
248.	Cannabis Sativa: Getting closer to separating the medicinal properties from the drug of abuse<br>Stella N<br>Journal of Neuroimmunology 2005<br>166(1-2):1-2 doi:10.1016/j.jneuroim.2005.05.005	Scopus	Discard<br>Reasons:<br>Not of interest
249.	Cannabis sativa: Much more beyond Δ9-tetrahydrocannabinol<br>Alves, P., Amaral, C., Teixeira, N., Correia-da-Silva, G<br>Pharmacological Research 2020<br>157 doi: 10.1016/j.phrs.2020.104822	Scopus<br>Web of Science	Discard<br>Reasons:<br>Not of interest
250.	Cannabis therapeutics and the future of neurology<br>Russo EB<br>Frontiers in Integrative Neuroscience 2018<br>12:51 doi:10.3389/fnint.2018.00051	Pubmed<br>Scopus<br>Web of Science	Discard<br>Reasons:<br>Not of interest
251.	Cannabis, pain, and sleep: lessons from therapeutic clinical trials of Sativex, a cannabis-based medicine<br>Russo EB, Guy GW, Robson PJ.<br>Chem Biodivers. 2007<br>4(8):1729-43	Pubmed<br>Scopus<br>Web of Science	Discard<br>Reasons:<br>Not of interest
252.	Cannabis, the mind and society: The hash realities<br>Murray RM, Morrison PD, Henquet C, Di Forti M<br>Nature Reviews Neuroscience 2007<br>8(11):885-895 doi:10.1038/nrn2253	Scopus	Discard<br>Reasons:<br>Not of interest
253.	Cannabis. 1. Effects of cannabinoids on the central nervous system. 2. Interactions with drugs [Cannabis. 1. ZNS-effekte durch cannabinoide. 2. Interaktionen mit arzneimitteln]<br>Bielenberg J<br>Arztezeitschrift fur Naturheilverfahren 2003<br>44(12):907-912	Scopus	Discard<br>Reasons:<br>Not of interest
254.	Cannabis: A neurological remedy or a drug of abuse in India<br>Biswas P, Mishra P, Bose D, Durgbanshi A<br>CNS and Neurological Disorders - Drug Targets 2017<br>16(5):576-584 doi:10.2174/1871527316666170424115008	Scopus	Discard<br>Reasons:<br>Not of interest
255.	Cannabis: A treasure trove or pandora’s box?<br>Solymosi K, Köfalv A	Scopus	Discard<br>Reasons:

	Mini-Reviews in Medicinal Chemistry 2017<br>17(13):1223-1291 doi:10.2174/1389557516666161004162133		Not of interest
256.	Cannabis: An overview of its adverse acute and chronic effects and its implications<br>Current Drug Abuse Reviews 2017<br>10(1):6-18 doi:10.2174/1874473710666170712113042	Scopus	Discard<br>Reasons:<br>Not of interest
257.	Cannabis: Old medicine with new promise for neurological disorders<br>Current Opinion in Investigational Drugs 2002<br>3(3):437-440	Scopus	Discard<br>Reasons:<br>Not of interest
258.	Cannabis: The never-ending, nefarious nepenthe of the 21st century: What should the clinician know?<br>Greydanus DE, Kaplan G, Baxter Sr LE, Patel DR, Feucht CL<br>Disease-a-Month 2015<br>61(4):118-175 doi:10.1016/j.disamonth.2015.01.004	Scopus	Discard<br>Reasons:<br>Not of interest
259.	Cannabis; Adverse effects from an oromucosal spray<br>Scully C<br>British Dental Journal 2007<br>203(6):13 doi:10.1038/bdj.2007.749	Pubmed<br>Scopus	Discard<br>Reasons:<br>Not of interest
260.	Cannabis-based medicines - GW Pharmaceuticals. High CBD, high THC, medicinal cannabis - GW Pharmaceuticals, THC:CBD<br>[No authors listed]<br>Drugs in R and D 2003<br>4(5):306-309 doi:10.2165/00126839-200304050-00005	Scopus	Discard<br>Reasons:<br>Not of interest
261.	Cannabis-based medicines for chronic neuropathic pain in adults.<br>Mucke M, Phillips T, Radbruch L, Petzke F, Hauser W.<br>COCHRANE DATABASE OF SYSTEMATIC REVIEWS 2018<br>(3): CD012182 doi: 10.1002/14651858.CD012182.pub2	Web of Science	Discard<br>Reasons:<br>Not of interest
262.	Cannabis-based medicines in multiple sclerosis - A review of clinical studies<br>Rog DJ<br>Immunobiology 2010<br>215(8):658-672 doi:10.1016/j.imbio.2010.03.009	Scopus	Discard<br>Reasons:<br>Not of interest
263.	Cannabis-based medicines on trial<br>Pharmaceutical Journal 2002<br>268:7183	Scopus	Discard<br>Reasons:<br>Not of interest
264.	Cannabis-based medicinal products: summary of NICE guidance<br>Chang-Douglass S, Mulvihill C, Pilling S<br>BMJ 2020<br>369:m1108. doi: 10.1136/bmj.m1108	Pubmed<br>Scopus	Discard<br>Reasons:<br>Not of interest

265.	Cannabis-based treatment induces polarity-reversing plasticity assessed by theta burst stimulation in humans.<br>Koch G, Mori F, CodecÃ C, Kusayanagi H, Monteleone F, Buttari F, Fiore S, Bernardi G, Centonze D.<br>Brain Stimul. 2009<br>2(4):229-33. doi: 10.1016/j.brs.2009.03.001.	Pubmed<br>Scopus	Discard<br>Reasons:<br>Not of interest
266.	Cannabislegemiddel i Norge-grunnlaget for godkjenning<br>Tidsskrift for den Norske Laegeforening 2012<br>132(23-24):2598-2599 doi:10.4045/tidsskr.12.1250	Pubmed<br>Scopus	Discard<br>Reasons:<br>Not of interest
267.	CB receptor ligands from plants<br>Woelkart K, Salo-Ahen OMH, Bauer R<br>Current Topics in Medicinal Chemistry 2008<br>8(3):173-186 doi:10.2174/156802608783498023	Scopus<br>Web of Science	Discard<br>Reasons:<br>Not of interest
268.	CBD Suppression of EAE Is Correlated with Early Inhibition of Splenic IFN-γ + CD8+ T Cells and Modest Inhibition of Neuroinflammation<br>Nichols JM, Kummari E, Sherman J, Yang EJ, Dhital S, Gilfeather C, Yray G, Morgan T, Kaplan BLF<br>J Neuroimmune Pharmacol 2020<br>doi: 10.1007/s11481-020-09917-8	Pubmed<br>Scopus<br>Web of Science	Keep
269.	Chemistry and pharmacotoxicity of cannabis: Recent data<br>Tesseromati C, Karikas GA<br>Pharmakeftiki 2019<br>31(3):124-156	Scopus	Discard<br>Reasons:<br>Not of interest
270.	Clinical and electrophysiological measures in a cohort of multiple sclerosis patients during treatment with nabiximols for spasticity.<br>Paolino I, Medici D, Zagaglia S, Cerqua R, Danni MC, Provinciali L.<br>Multiple Sclerosis Journal 2013<br>19(11):281-281 Supplement: S	Web of Science	Discard<br>Reasons:<br>Not of interest
271.	Clinical case reviews and poster sessions in multiple sclerosis spasticity: main outcomes and highlights.<br>Trojano M, Celius EG, Donzé C, Izquierdo G, Patti F, Pöhlau D.<br>Eur Neurol. 2014 72 Suppl 1:15-9. doi: 10.1159/000367619.	Pubmed<br>Scopus	Discard<br>Reasons:<br>Not of interest
272.	Clinical case reviews in multiple sclerosis spasticity: experiences from around Europe<br>Koehler J, Amato MP, Oreja-Guevara C, Lycke J.<br>Expert Rev Neurother. 2013<br>13(12 Suppl):61-6. doi: 10.1586/14737175.2013.865881.	Pubmed<br>Scopus<br>Web of Science	Discard<br>Reasons:<br>Not of interest

273.	Clinical Data for the Use of Cannabis-Based Treatments: A Comprehensive Review of the Literature<br>Inglet S, Winter B, Yost SE, Entringer S, Lian A, Biksacky M, Pitt RD, Mortensen W.<br>Ann Pharmacother. 2020<br>doi: 10.1177/1060028020930189	Pubmed<br>Scopus<br>Web of Science	Discard<br>Reasons:<br>Not of interest
274.	Clinical efficacy and effectiveness of Sativex ®, a combined cannabinoid medicine, in multiple sclerosis-related spasticity<br>Oreja-Guevara C<br>Expert Review of Neurotherapeutics 2012<br>12(4) SUPPL. 3-8 doi:10.1586/ern.12.11	Pubmed<br>Scopus	Discard<br>Reasons:<br>Not of interest
275.	Clinical Endocannabinoid Deficiency Reconsidered: Current Research Supports the Theory in Migraine, Fibromyalgia, Irritable Bowel, and Other Treatment-Resistant Syndromes.<br>Russo EB.<br>Cannabis Cannabinoid Res. 2016<br>1(1):154-165. doi: 10.1089/can.2016.0009	Pubmed<br>Scopus	Discard<br>Reasons:<br>Not of interest
276.	Clinical experience with THC: CBD oromucosal spray in patients with multiple sclerosis-related spasticity.<br>Koehler J, Feneberg W, Meier M, Pollmann,W<br>International Journal of Neuroscience 2014<br>124(9): 652-656 doi: 10.3109/00207454.2013.877460	Pubmed<br>Scopus<br>Web of Science	Discard<br>Reasons:<br>Not of interest
277.	Clinical experiences with cannabinoids in spasticity management in multiple sclerosis.<br>Lorente Fernández L, Monte Boquet E, Pérez-Miralles F, Gil Gómez I, Escutia Roig M, Boscá Blasco I, Poveda Andrés JL, Casanova-Estruch B.<br>Neurologia. 2014<br>29(5):257-60. doi: 10.1016/j.nrl.2013.06.014.	Pubmed<br>Scopus<br>Web of Science	Discard<br>Reasons:<br>Not of interest
278.	Clinical experiences with on-label nabiximols therapy in multiple sclerosis-induced spasticity.<br>Koehler J, Feneberg W, Gorodetzky H, Meier M, Pollmann W.<br>MULTIPLE SCLEROSIS JOURNAL 2013<br>19(11): 281-282 Supplement: S	Web of Science	Discard<br>Reasons:<br>Not of interest
279.	Clinical perspectives on medical marijuana (cannabis) for neurologic disorders<br>Fife TD, Moawad H, Moschonas C, Shepard K, Hammond N<br>Neurology: Clinical Practice 2015<br>5(4): 344-351 doi:10.1212/CPJ.0000000000000162	Scopus	Discard<br>Reasons:<br>Not of interest
280.	Clinical Response to Nabiximols (Sativex ®) on Spasticity and Pain Is Paralleled by a Down-Regulation of Immune-Related Pathways in Multiple Sclerosis Patients.	Web of Science	Discard<br>Reasons:

	Boneschi FM, Sorosina M, Ferrè I, Clarelli F, Martinelli V, Esposito F, Comi G.<br>NEUROLOGY 2016<br>86 Supplement: 16 Meeting Abstract: P2.169		Not of interest
281.	Clinical response to Nabiximols correlates with the downregulation of immune pathways in multiple sclerosis<br>Sorosina M, Clarelli F, Ferrè L, Osiceanu AM, Unal NT, Mascia E, Martinelli V, Comi G, Benigni F, Esposito F, Martinelli Boneschi F.<br>Eur J Neurol. 2018<br>25(7):934-e70. doi: 10.1111/ene.13623	Pubmed<br>Scopus<br>Web of Science	Keep
282.	Clinical Use of Cannabinoids for Symptom Control in Multiple Sclerosis<br>Notcutt WG.<br>Neurotherapeutics 2015<br>12(4):769-77. doi: 10.1007/s13311-015-0383-5.	Pubmed<br>Scopus<br>Web of Science	Discard<br>Reasons:<br>Not of interest
283.	Clinical/therapeutic approaches for cannabinoid ligands in central and peripheral nervous system diseases: Mini review<br>Fija, K, Filip M.<br>Clinical Neuropharmacology 2016<br>39(2):94-101 doi:10.1097/WNF.0000000000000132	Pubmed<br>Scopus<br>Web of Science	Discard<br>Reasons:<br>Not of interest
284.	CNS immune surveillance and neuroinflammation: Endocannabinoids keep control<br>Wolf SA, Tauber S, Ullrich O<br>Current Pharmaceutical Design 2008<br>14(23):2266-2278 doi:10.2174/138161208785740090	Scopus	Discard<br>Reasons:<br>Not of interest
285.	Combination of Cannabinoids, Delta 9-Tetrahydrocannabinol and Cannabidiol, Ameliorates Experimental Multiple Sclerosis by Suppressing Neuroinflammation Through Regulation of miRNA-Mediated Signaling Pathways.<br>Al-Ghezi ZZ, Miranda K, Nagarkatti M, Nagarkatti PS<br>Frontiers in Immunology 2019<br>10: 1921 doi: 10.3389/fimmu.2019.01921	Pubmed<br>Scopus<br>Web of Science	Keep
286.	Combination of cannabinoids, delta-9-tetrahydrocannabinol (THC) and cannabidiol (CBD), mitigates experimental autoimmune encephalomyelitis (EAE) by altering the gut microbiome<br>Al-Ghezi ZZ, Busbee PB, Alghetaa H, Nagarkatti PS, Nagarkatti M.<br>Brain Behav Immun. 2019<br>82:25-35. doi: 10.1016/j.bbi.2019.07.028	Pubmed<br>Scopus<br>Web of Science	Keep
287.	Combined cannabinoid therapy via an oromucosal spray<br>Perez J<br>Drugs of Today 2006	Scopus<br>Web of Science	Discard<br>Reasons:<br>Not of interest

	42(8):495-503 doi:10.1358/dot.2006.42.8.1021517		
288.	Comparison of orally administered cannabis extract and delta-9-tetrahydrocannabinol in treating patients with cancer-related anorexia-cachexia syndrome: A multicenter, phase III, randomized, double-blind, placebo-controlled clinical trial from the cannabis-in-cachexia-study-group.<br>Strasser F, Luftner D, Possinger K, Ernst G, Ruhstaller T, Meissner W, Ko YD, Schnelle M, Reif M, Cerny T<br>Journal of Clinical Oncology 2006<br>24(21): 3394-3400 doi: 10.1200/JCO.2005.05.1847	Web of Science	Discard<br>Reasons:<br>Not of interest
289.	Complementary and alternative medical therapies in multiple sclerosis--the American Academy of Neurology guidelines: a commentary<br>Yadav V, Narayanaswami P.<br>Clin Ther. 2014<br>36(12):1972-1978. doi: 10.1016/j.clinthera.2014.10.011.	Pubmed<br>Scopus<br>Web of Science	Discard<br>Reasons:<br>Not of interest
290.	Complementary and alternative medicine: Is there a role in multiple sclerosis?<br>Yadav V, Bourdette D<br>Current Neurology and Neuroscience Reports 2006<br>6(3):259-267 doi:10.1007/s11910-006-0014-y	Scopus	Discard<br>Reasons:<br>Not of interest
291.	Comprehensive Review of Medicinal Marijuana, Cannabinoids, and Therapeutic Implications in Medicine and Headache: What a Long Strange Trip It's Been.<br>Baron EP<br>Headache 2015<br>55(6): 885-916 doi: 10.1111/head.12570	Web of Science	Discard<br>Reasons:<br>Not of interest
292.	Conditional okay for cannabis prescription drug<br>Sibbald B<br>CMAJ 2005<br>172(13):1672 doi:10.1503/cmaj.050628	Pubmed<br>Scopus	Discard<br>Reasons:<br>Not of interest
293.	Consensus document on spasticity in patients with multiple sclerosis [Documento de consenso sobre la espasticidad en pacientes con esclerosis múltiple]<br>Oreja-Guevara C, Montalban X, de Andrés C, Casanova-Estruch B, Muñoz-García D, García I, Fernández O<br>Revista de Neurologia 2013<br>57(8):359-373 doi: 10.33588/rn.5708.2013374	Scopus<br>Web of Science	Discard<br>Reasons:<br>Not of interest
294.	Control of spasticity in a multiple sclerosis model using central nervous system-excluded CB1 cannabinoid receptor agonists	Scopus	Discard<br>Reasons:<br>Not of interest

	Pryce G, Visintin C, Ramagopalan SV, Al-Izki S, De Faveri LE., Nuamah RA, Mein CA, Montpetit A, Hardcastle AJ, Kooij G, de Vries HE, Amor S, Thomas SA, Ledent C, Marsicano G, Lutz B, Thompson AJ, Selwood DL, Giovannoni G, Baker D<br>FASEB Journal 2014<br>28(1):117-130 doi:10.1096/fj.13-239442		
295.	Cortical and spinal excitability in patients with multiple sclerosis and spasticity after oromucosal cannabinoid spray<br>Squintani G, Donato F, Turri M, Deotto L, Teatini F, Moretto G, Erro R.<br>J Neurol Sci. 2016<br>370:263-268. doi: 10.1016/j.jns.2016.09.054.	Pubmed<br>Scopus<br>Web of Science	Discard<br>Reasons:<br>Not of interest
296.	Cortical excitability changes: A mirror to the natural history of multiple sclerosis? [Les modifications de l'excitabilité corticale reflètent-elles l'histoire naturelle de la sclérose en plaques ?<br>Ayache SS, Chalah MA<br>Neurophysiologie Clinique 2017<br>47(3):221-223 doi:10.1016/j.neucli.2017.02.001	Scopus	Discard<br>Reasons:<br>Not of interest
297.	Cost effectiveness of oromucosal cannabis-based medicine (SativexÂ®) for spasticity in multiple sclerosis<br>Lu L, Pearce H, Roome C, Shearer J, Lang IA, Stein K.<br>Pharmacoeconomics. 2012<br>30(12):1157-71. doi: 10.2165/11598470-000000000-00000.	Pubmed<br>Scopus	Discard<br>Reasons:<br>Not of interest
298.	Cost-Effectiveness Analysis of Cannabinoid Oromucosal Spray Use for the Management of Spasticity in Subjects with Multiple Sclerosis<br>Clin Drug Investig 2020<br>40(4):319-326. doi: 10.1007/s40261-020-00895-6.2020	Scopus	Discard<br>Reasons:<br>Not of interest
299.	Cost-effectiveness of Sativex in multiple sclerosis spasticity: new data and application to Italy<br>Slof J, Ruiz L, Vila C.<br>Expert Rev Pharmacoecon Outcomes Res. 2015<br>15(3):379-91. doi: 10.1586/14737167.2015.1025759.	Pubmed<br>Scopus<br>Web of Science	Discard<br>Reasons:<br>Not of interest
300.	Country breakout session highlights<br>Ghezzi A, Filli L, Solaro C, Mekies C, Landete L, Lycke J<br>Neurodegenerative disease management 2016<br>6(6):41-44	Pubmed<br>Scopus	Discard<br>Reasons:<br>Not of interest
301.	Critical Role of Mast Cells and Peroxisome Proliferator-Activated Receptor gamma in the Induction of Myeloid-Derived Suppressor Cells by Marijuana Cannabidiol In Vivo.<br>Hegde VL, Singh UP, Nagarkatti PS, Nagarkattit M	Web of Science	Discard<br>Reasons:<br>Not of interest

	JOURNAL OF IMMUNOLOGY 2015<br>194(11): 5211-5222 DOI: 10.4049/jimmunol.1401844		
302.	Current and future drugs for treatment of MS-associated bladder dysfunction<br>Andersson KE<br>Annals of Physical and Rehabilitation Medicine 2014<br>57(5):321-328 doi:10.1016/j.rehab.2014.05.009	Scopus	Discard<br>Reasons:<br>Not of interest
303.	Current application of cannabidiol (CBD) in the management and treatment of neurological disorders<br>Fiani B, Sarhadi KJ, Soula M, Zafar A, Quadri SA.<br>Neurol Sci. 2020<br>doi: 10.1007/s10072-020-04514-2	Pubmed<br>Scopus<br>Web of Science	Discard<br>Reasons:<br>Not of interest
304.	Current evidence of cannabinoid-based analgesia obtained in preclinical and human experimental settings.<br>Lotsch J, Weyer-Menkhoff I, Tegeder I<br>EUROPEAN JOURNAL OF PAIN 2018<br>22(3): 471-484 DOI: 10.1002/ejp.1148	Web of Science	Discard<br>Reasons:<br>Not of interest
305.	Current spasticity therapy in multiple sclerosis: Practical experiences using nabiximols [Aktuelle Spastikbehandlung am Beispiel der multiplen Sklerose: Erfahrungen mit Nabiximols]<br>Psychopharmakotherapie 2015<br>22(2):84-90	Scopus	Discard<br>Reasons:<br>Not of interest
306.	Current status of cannabis treatment of multiple sclerosis with an illustrative case presentation of a patient with MS, complex vocal tics, paroxysmal dystonia, and marijuana dependence treated with dronabinol.<br>Deutsch SI, Rosse RB, Connor JM, Burket JA, Murphy ME, Fox FJ.<br>CNS Spectr. 2008<br>13(5):393-403 doi: 10.1017/S1092852900016564	Pubmed<br>Scopus<br>Web of Science	Discard<br>Reasons:<br>Not of interest
307.	Current Therapeutic Cannabis Controversies and Clinical Trial Design Issues.<br>Russo EB<br>FRONTIERS IN PHARMACOLOGY 2016<br>7: 309 DOI: 10.3389/fphar.2016.00309	Web of Science	Discard<br>Reasons:<br>Not of interest
308.	Daily Practice Managing Resistant Multiple Sclerosis Spasticity With Delta-9-Tetrahydrocannabinol: Cannabidiol Oromucosal Spray: A Systematic Review of Observational Studies<br>Akgün K, Essner U, Seydel C, Ziemssen T.<br>J Cent Nerv Syst Dis. 2019<br>11:1179573519831997. doi: 10.1177/1179573519831997.	Scopus<br>Web of Science	Discard<br>Reasons:<br>Not of interest

309.	Dark Classics in Chemical Neuroscience: Delta(9)-Tetrahydrocannabinol.<br>Banister SD, Arnold JC, Connor M, Glass M, McGregor IS<br>ACS CHEMICAL NEUROSCIENCE 2019<br>10(5): 2160-2175 doi: 10.1021/acschemneuro.8b00651	Pubmed<br>Scopus<br>Web of Science	Discard<br>Reasons:<br>Not of interest
310.	Delta-9-tetrahydrocannabinol + cannabidiol (New Drug)<br>[No author name available]<br>Prescrire International 2014<br>23(150):145-148	Pubmed<br>Scopus	Discard<br>Reasons:<br>Not of interest
311.	Delta-9-tetrahydrocannabinol and cannabidiol: Separating the chemicals from the "weed," a pharmacodynamic discussion<br>Boggs DL, Peckham A, Boggs AA, Ranganathan M.<br>Ment Health Clin. 2016<br>6(6):277-284. doi: 10.9740/mhc.2016.11.277.	Pubmed	Discard<br>Reasons:<br>Not of interest
312.	Delta-9-Tetrahydrocannabinol/Cannabidiol (Sativex((R))): A Review of Its Use in Patients with Moderate to Severe Spasticity Due to Multiple Sclerosis.<br>Syed YY, McKeage K, Scott LJ<br>DRUGS 2014<br>74(5): 563-578 DOI: 10.1007/s40265-014-0197-5	Pubmed<br>Scopus<br>Web of Science	Discard<br>Reasons:<br>Not of interest
313.	Delta-9-Tetrahydrocannabinol/Cannabidiol Oromucosal Spray (Sativex(Â®)): A Review in Multiple Sclerosis-Related Spasticity<br>Keating GM.<br>Drugs. 2017<br>77(5):563-574. doi: 10.1007/s40265-017-0720-6.	Pubmed<br>Scopus<br>Web of Science	Discard<br>Reasons:<br>Not of interest
314.	Delta-9-tetrahydrocannabinol-cannabidiol in the treatment of spasticity in chronic spinal cord injury: A clinical experience [Delta-9-tetrahidrocannabinol-cannabidiol en el tratamiento de la espasticidad en la lesión medular crónica: Una experiencia clínica]<br>Grao-Castellote C, Torralba-Collados F, Gonzalez LM, Giner-Pascual M<br>Revista de Neurologia 2017<br>65(7):295-302 doi: 10.33588/rn.6507.2017118	Scopus<br>Web of Science	Discard<br>Reasons:<br>Not of interest
315.	Dendropanax morbiferus leaf extract facilitates oligodendrocyte development<br>Kim JY, Yoon JY, Sugiura Y, Lee SK, Park JD, Song GJ, Yang HJ.<br>R Soc Open Sci. 2019<br>6(6):190266. doi: 10.1098/rsos.190266.	Pubmed<br>Scopus<br>Web of Science	Discard<br>Reasons:<br>Not of interest
316.	Der er evidens for brug af cannabinoider til symptomatisk behandling af multipel sklerose<br>Basinski H, Jensen H.B, Stenager E<br>Ugeskrift for Laeger 2015<br>177(10):956-960	Scopus	Discard<br>Reasons:<br>Not of interest

317.	Determinants of botulinum toxin discontinuation in multiple sclerosis: a retrospective study<br>Latino P, Castelli L, Prosperini L, Marchetti MR, Pozzilli C, Giovannelli M<br>Neurological Sciences 2017<br>38(10):1841-1848 doi:10.1007/s10072-017-3078-3	Scopus	Discard<br>Reasons:<br>Not of interest
318.	Development of a primary cutaneous CD30(+) anaplastic large-cell T-cell lymphoma during treatment of multiple sclerosis with fingolimod<br>Papathemeli D, Gräfe R, Hildebrandt U, Zettl UK, Ulrich J<br>Multiple Sclerosis 2016<br>22(14):1888-1890 doi:10.1177/1352458516645868	Scopus	Discard<br>Reasons:<br>Not of interest
319.	Dexanabinol Pharmos<br>Pop E<br>Current Opinion in Investigational Drugs 2000<br>1(4):494-503	Scopus	Discard<br>Reasons:<br>Not of interest
320.	Dexanabinol: A novel cannabinoid with neuroprotective properties<br>Darlington CL<br>IDrugs 2003<br>6(10):976-979	Scopus	Discard<br>Reasons:<br>Not of interest
321.	Dietary fats and pharmaceutical lipid excipients increase systemic exposure to orally administered cannabis and cannabis-based medicines.<br>Zgair A, Wong JCM, Lee JB, Mistry J, Sivak O, Wasan KM, Hennig IM, Barrett DA, Constantinescu CS, Fischer PM, Gershkovich P<br>AMERICAN JOURNAL OF TRANSLATIONAL RESEARCH 2016<br>8(8): 3448-3459	Web of Science	Discard<br>Reasons:<br>Not of interest
322.	Differential transcriptional profiles mediated by exposure to the cannabinoids cannabidiol and Delta (9)-tetrahydrocannabinol in BV-2 microglial cells.<br>Juknat A, Pietr M, Kozela E, Rimmerman N, Levy R, Coppola G, Geschwind D, Vogel Z<br>BRITISH JOURNAL OF PHARMACOLOGY 2012<br>165(8):2512-2528 doi: 10.1111/j.1476-5381.2011.01461.x	Web of Science	Discard<br>Reasons:<br>Not of interest
323.	"Disease modifying nutricals" for multiple sclerosis<br>Schmitz K, Barthelmes J, Stolz L, Beyer S, Diehl O, Tegeder I<br>Pharmacology and Therapeutics 2015<br>148:85-113 doi:10.1016/j.pharmthera.2014.11.015	Scopus	Discard<br>Reasons:<br>Not of interest
324.	Distinct interactions of cannabidiol and morphine in three nociceptive behavioral models in mice.<br>Neelakantan H, Tallarida RJ, Reichenbach ZW, Tuma RF, Ward SJ, Walker EA<br>BEHAVIOURAL PHARMACOLOGY 2015	Web of Science	Discard<br>Reasons:<br>Not of interest

	26(3): 304-314 doi: 10.1097/FBP.0000000000000119		
325.	Divergent effects of cannabidiol on the discriminative stimulus and place conditioning effects of Delta (9)-tetrahydrocannabinol<br>Vann RE, Gamage TF, Warner JA, Marshall EM, Taylor NL, Martin BR, Wiley JL.<br>Drug Alcohol Depend. 2008<br>94(1-3):191-8. doi: 10.1016/j.drugalcdep.2007.11.017	Pubmed<br>Scopus<br>Web of Science	Discard<br>Reasons:<br>Not of interest
326.	DMH-CBD, a cannabidiol analog with reduced cytotoxicity, inhibits TNF production by targeting NF-kB activity dependent on A(2A) receptor.<br>Silva RL, Silveira GT, Wanderlei CW, Cecilio NT, Maganin AGM, Franchin M, Marques LMM, Lopes NP, Crippa, JA, Guimaraes FS; Alves JCF, Cunha FQ, Cunha TM<br>TOXICOLOGY AND APPLIED PHARMACOLOGY 2019<br>368: 63-71 doi: 10.1016/j.taap.2019.02.011	Web of Science	Discard<br>Reasons:<br>Not of interest
327.	Do cannabinoids confer neuroprotection against epilepsy? An overview<br>Capasso A<br>Open Neurology Journal 2017,<br>11(61):73 doi:10.2174/1874205X01711010061	Scopus	Discard<br>Reasons:<br>Not of interest
328.	Do cannabis-based medicinal extracts have general or specific effects on symptoms in multiple sclerosis? A double-blind, randomized, placebo-controlled study on 160 patients<br>Wade DT, Makela P, Robson P, House H, Bateman C.<br>Mult Scler. 2004<br>10(4):434-41	Pubmed<br>Scopus<br>Web of Science	Discard<br>Reasons:<br>Not of interest
329.	Dronabinol and chronic pain: Importance of mechanistic considerations<br>De Vries M, Van Rijckevorsel DC, Wilder-Smith OH, Van Goor H<br>Expert Opinion on Pharmacotherapy 2014<br>15(11):1525-1534 doi:10.1517/14656566.2014.918102	Scopus	Discard<br>Reasons:<br>Not of interest
330.	Dronabinol/Cannabidiol<br>Mealy NE, Bayés M<br>Drugs of the Future 2004 Short survey<br>29(3):282	Scopus	Discard<br>Reasons:<br>Not of interest
331.	Dronabinol/Cannabidiol<br>Mealy NE, Bayés M<br>Drugs of the Future 2005 Note<br>30(2):198	Scopus	Discard<br>Reasons:<br>Not of interest
332.	Drug discovery strategies that focus on the endocannabinoid signaling system in psychiatric disease<br>Expert Opinion on Drug Discovery 2015<br>10(1):17-36 doi:10.1517/17460441.2014.966680	Scopus	Discard<br>Reasons:<br>Not of interest

333.	Drug-resistant MS spasticity treatment with Sativex (R) add-on and driving ability.<br>Freidel M, Tiel-Wilck K, Schreiber H, Prechtl A, Essner U, Lang M<br>ACTA NEUROLOGICA SCANDINAVICA<br>131(1): 9-16 DOI: 10.1111/ane.12287	Pubmed<br>Scopus<br>Web of Science	Discard<br>Reasons:<br>Not of interest
334.	Drugs for chronic pain in children: A commentary on clinical practice and the absence of evidence<br>Pain Research and Management 2013<br>18(1):47-50 doi:10.1155/2013/402863	Scopus	Discard<br>Reasons:<br>Not of interest
335.	Early Treatment of Experimental Autoimmune Encephalomyelitis with Cannabidiol Suppresses Tc1 Cells and Reduces Neuroinflammation.<br>Nichols JM, Kummari E, Sherman J, Szafran B, Kaplan B<br>JOURNAL OF IMMUNOLOGY 2018<br>200(1 Supplement): 121.10	Web of Science	Discard<br>Reasons: Not of interest
336.	Editorial<br>Kullmann DM<br>Brain 2019<br>142(9): 2545 doi:10.1093/brain/awz253	Scopus	Discard<br>Reasons:<br>Not of interest
337.	Editors' welcome<br>Banwell B, Giovannoni G, Hawkes C, Lublin F<br>Multiple Sclerosis and Related Disorders 2012<br>1(2):55 doi:10.1016/j.msard.2012.01.005	Scopus	Discard<br>Reasons:<br>Not of interest
338.	Effect of cannabidiolic acid and Delta(9)-tetrahydrocannabinol on carrageenan-induced hyperalgesia and edema in a rodent model of inflammatory pain.<br>Rock EM, Limebeer CL, Parker LA<br>PSYCHOPHARMACOLOGY 2018<br>235(11): 3259-3271 DOI: 10.1007/s00213-018-5034-1	Web of Science	Discard<br>Reasons:<br>Not of interest
339.	Effects of EHP-101 on inflammation and remyelination in murine models of Multiple sclerosis<br>Navarrete C, García-Martin A, Garrido-Rodriguez M, Mestre L, Feliu A, Guaza C, Calzado MA, Muñoz E<br>Neurobiol Dis. 2020<br>143:104994 doi: 10.1016/j.nbd.2020.104994	Pubmed<br>Scopus	Discard<br>Reasons:<br>Not of interest
340.	Effect of Sativex on spasticity-associated symptoms in patients with multiple sclerosis<br>Meuth SG, Vila C, Dechant KL.<br>Expert Rev Neurother. 2015<br>15(8):909-18. doi: 10.1586/14737175.2015.1067607.	Pubmed<br>Web of Science	Discard<br>Reasons:<br>Not of interest

341.	Effects of THC/CBD oromucosal spray on spasticity-related symptoms in people with multiple sclerosis: results from a retrospective multicenter study<br>Patti F, Chisari CG, Solaro C, Benedetti MD, Berra E, Bianco A, Bruno Bossio R, Buttari F, Castelli L, Cavalla P, Cerqua R, Costantino G, Gasperini C, Guareschi A, Ippolito D, Lanzillo R, Maniscalco GT, Matta M, Paolicelli D, Petrucci L, Pontecorvo S, Righini I, Russo M, SaccÃ F, Salamone G, Signoriello E, Spinicci G, Spitaleri D, Tavazzi E, Trotta M, Zaffaroni M, Zappia M<br>Neurological Sciences 2020<br>doi: 10.1007/s10072-020-04413-6	Pubmed<br>Scopus<br>Web of Science	Discard<br>Reasons:<br>Not of interest
342.	Effect of tetrahydrocannabinol: cannabidiol oromucosal spray on activities of daily living in multiple sclerosis patients with resistant spasticity: a retrospective, observational study.<br>Frechin JM<br>NEURODEGENERATIVE DISEASE MANAGEMENT 2018<br>8(3): 151-159 doi: 10.2217/nmt-2017-0055	Pubmed<br>Scopus<br>Web of Science	Discard<br>Reasons:<br>Not of interest
343.	Effectiveness and safety of Tetrahydrocannabinol - Cannabidiol Oromucosal spray in Belgian patients with multiple sclerosis spasticity: a retrospective data collection.<br>Popescu V, Vos C, van Dael V, Raeymaekers G, van Wijmeersch B<br>MULTIPLE SCLEROSIS JOURNAL 2019<br>Meeting Abstract: P693 25 Special Issue: SI 346-346 Supplement: 2	Web of Science	Discard<br>Reasons:<br>Not of interest
344.	Effectiveness and Tolerability of THC/CBD Oromucosal Spray for Multiple Sclerosis Spasticity in Italy: First Data from a Large Observational Study<br>Trojano M, Vila C.<br>Eur Neurol. 2015<br>74(3-4):178-85. doi: 10.1159/000441619.	Pubmed<br>Scopus<br>Web of Science	Discard<br>Reasons:<br>Not of interest
345.	Effectiveness and tolerability of THC:CBD oromucosal spray as add-on measure in patients with severe chronic pain: analysis of 12-week open-label real-world data provided by the German Pain e-Registry.<br>Ueberall MA, Essner U, Mueller-Schwefe GHH<br>JOURNAL OF PAIN RESEARCH 2019<br>12: 1577-1604 doi: 10.2147/JPR.S192174	Web of Science	Discard<br>Reasons:<br>Not of interest
346.	Effectiveness, adverse effects, and safety of medical marijuana<br>Keehbauch J, Rensberry M<br>American Family Physician 2015<br>92(10)	Scopus	Discard<br>Reasons:<br>Not of interest
347.	Effects of cannabidiol on brain excitation and inhibition systems; a randomised placebo-controlled single dose trial during magnetic resonance spectroscopy in adults with and without autism spectrum disorder.	Web of Science	Discard<br>Reasons:<br>Not of interest

	Pretzsch CM, Freyberg J, Voinescu B, Lythgoe D, Horder J, Mendez MA, Wichers R, Ajram L, Ivin G, Heasman M, Edden RAE, Williams S Murphy DGM, Daly E, McAlonan GM<br>Neuropsycopharmacology 2019<br>44(8): 1398-1405 doi: 10.1038/s41386-019-0333-8		
348.	Effects of cannabinoid administration for pain: A meta-analysis and meta-regression<br>Yanes JA, McKinnell ZE, Reid MA, Busler JN, Michel JS, Pangelinan MM, Sutherland MT, Younger JW, Gonzalez R, Robinson JL<br>Experimental and Clinical Psychopharmacology 2019<br>27(4):370-382 doi:10.1037/pha0000281	Scopus	Discard<br>Reasons:<br>Not of interest
349.	Effects of Sativex and neuromotor rehabilitation treatment: case report with cerebral palsy and supervening multiple sclerosis.<br>Camerano C, Camuzzini N, Lentino C, Checchia GA<br>42ND CONGRESSO NAZIONALE SIMFER 2015<br>38-40	Web of Science	Discard<br>Reasons:<br>Not of interest
350.	Efficacy and discontinuation of nabiximols in patients with multiple sclerosis: a real-life study.<br>Bergamaschi V, Konrad G, Battaglia MA, Brichetto G<br>Multiple Sclerosis Journal 2018<br>Meeting Abstract: EP1698 24: 959-959 Supplement: 2	Web of Science	Discard<br>Reasons:<br>Not of interest
351.	Efficacy and safety of cannabinoid oromucosal spray for multiple sclerosis spasticity<br>Patti F, Messina S, Solaro C, Amato MP, Bergamaschi R, Bonavita S, Bruno Bossio R, Brescia Morra V, Costantino GF, Cavalla P, Centonze D, Comi G, Cottone S, Danni M, Francia A, Gajofatto A, Gasperini C, Ghezzi A, Iudice A, Lus G, Maniscalco GT, Marrosu MG, et al.<br>J Neurol Neurosurg Psychiatry. 2016<br>87(9):944-51. doi: 10.1136/jnnp-2015-312591.	Pubmed<br>Scopus<br>Web of Science	Discard<br>Reasons:<br>Not of interest
352.	Efficacy and safety of nabiximols (Sativex (R)) on multiple sclerosis spasticity in a real-life Italian monocentric study.<br>Ferre L, Nuara A, Pavan G, Radaelli M, Moiola L, Rodegher M, Colombo B, Sarmiento IJK, Martinelli V, Leocani L, Boneschi FM, Comi G, Esposito F<br>Neurological Sciences 2016<br>37(2):235-242 doi: 10.1007/s10072-015-2392-x	Pubmed<br>Scopus<br>Web of Science	Discard<br>Reasons:<br>Not of interest
353.	Efficacy and Tolerability of Phytomedicines in Multiple Sclerosis Patients: A Review.<br>Farzaei MH, Shahpiri Z, Bahramsoltani R, Nia MM, Najafi F, Rahimi R<br>CNS DRUGS 2017	Pubmed<br>Scopus<br>Web of Science	Discard<br>Reasons:<br>Not of interest

	31(10): 867-889 DOI: 10.1007/s40263-017-0466-4		
354.	Efficacy of Cannabidiol: Clinical Studies with Cannabidiol and Cannabidiol-Containing Extracts [Wirksamkeit von Cannabidiol: Klinische Studien mit Cannabidiol und Cannabidiol-haltigen Extrakten]<br>Büechi S<br>Schweizerische Zeitschrift fur GanzheitsMedizin 2017,<br>29(6):367-371 doi:10.1159/000484886	Scopus<br>Web of Science	Discard<br>Reasons:<br>Not of interest
355.	Efficacy, safety and tolerability of an orally administered cannabis extract in the treatment of spasticity in patients with multiple sclerosis: a randomized, double-blind, placebo-controlled, crossover study<br>Vaney C, Heinzel-Gutenbrunner M, Jobin P, Tschopp F, Gattlen B, Hagen U, Schnelle M, Reif M.<br>Mult Scler. 2004<br>10(4):417-24 doi: 10.1191/1352458504ms1048oa	Pubmed<br>Scopus<br>Web of Science	Discard<br>Reasons:<br>Not of interest
356.	Efficacy, tolerability and safety of cannabinoids for chronic neuropathic pain. A systematic review of randomized controlled studies.<br>Petzke F, Enax-Krumova EK, Hauser W<br>SCHMERZ 2016<br>30(1): 62-88 doi: 10.1007/s00482-015-0089-y	Web of Science	Discard<br>Reasons:<br>Not of interest
357.	Emerging properties of cannabinoid medicines in management of multiple sclerosis.<br>Pryce G, Baker D<br>Trends in Neurosciences 2005<br>28(5): 272-276 doi: 10.1016/j.tins.2005.03.006	Web of Science	Discard<br>Reasons:<br>Not of interest
358.	Emerging strategies for exploiting cannabinoid receptor agonists as medicines<br>Pertwee RG<br>Br J Pharmacol. 2009<br>156(3):397-411. doi: 10.1111/j.1476-5381.2008.00048.x.	Pubmed<br>Scopus<br>Web of Science	Discard<br>Reasons:<br>Not of interest
359.	Emerging strategies targeting CB2 cannabinoid receptor: Biased agonism and allosterism<br>Biochemical Pharmacology 2018,<br>157:8-17 doi:10.1016/j.bcp.2018.07.031	Scopus	Discard<br>Reasons:<br>Not of interest
360.	Endocannabinoid pathways and their role in multiple sclerosis-related muscular dysfunction<br>Di Marzo V.<br>Expert Rev Neurother. 2011<br>11(4 Suppl):9- 14 doi: 10.1586/ern.11.26	Pubmed<br>Scopus	Discard<br>Reasons:<br>Not of interest
361.	Endocannabinoid system and psychiatry: In search of a neurobiological basis for detrimental and potential therapeutic effects	Scopus	Discard<br>Reasons:

	Marco EM., García-Gutiérrez MS, Bermúdez-Silva FJ, Moreira FA, Guimarães F, Manzanares J, Viveros M.-P<br>Frontiers in Behavioral Neuroscience 2011<br>67 doi:10.3389/fnbeh.2011.00063		Not of interest
362.	Endocannabinoid system and stress and anxiety responses<br>Viveros MP, Marco EM., File SE<br>Pharmacology Biochemistry and Behavior 2005<br>81(2) SPEC. ISS.:331-342 doi:10.1016/j.pbb.2005.01.029	Scopus	Discard<br>Reasons:<br>Not of interest
363.	Endocannabinoid system dysfunction in mood and related disorders.<br>Ashton CH, Moore PB<br>ACTA PSYCHIATRICA SCANDINAVICA 2011<br>124(4): 250-261 DOI: 10.1111/j.1600-0447.2011.01687.x	Web of Science	Discard<br>Reasons:<br>Not of interest
364.	Endocannabinoid system modulator use in everyday clinical practice in the UK and Spain<br>García-Merino A.<br>Expert Rev Neurother. 2013<br>13(3 Suppl 1):9-13. doi: 10.1586/ern.13.4.	Pubmed<br>Scopus<br>Web of Science	Discard<br>Reasons:<br>Not of interest
365.	Endocannabinoid system: A multi-facet therapeutic target<br>Kaur R, Ambwani SR, Singh S<br>Current Clinical Pharmacology 2016<br>11(2):110-117 doi:10.2174/1574884711666160418105339	Scopus<br>Web of Science	Discard<br>Reasons:<br>Not of interest
366.	Endocannabinoids and multiple sclerosis: A blessing from the 'inner bliss'?<br>Di Marzo V, Bifulco M, De Petrocellis L<br>Trends in Pharmacological Sciences 2000<br>21(6):195-197 doi:10.1016/S0165-6147(00)01487-5	Scopus	Discard<br>Reasons:<br>Not of interest
367.	Endocannabinoids and neurodegenerative diseases<br>Micale V, Mazzola C, Drago F<br>Pharmacological Research 2007<br>56(5):382-392 doi:10.1016/j.phrs.2007.09.008	Scopus	Discard<br>Reasons:<br>Not of interest
368.	Endocannabinoids in multiple sclerosis and amyotrophic lateral sclerosis<br>Pryce G, Baker D<br>Handbook of Experimental Pharmacology 2015<br>231:213-231 doi:10.1007/978-3-319-20825-1_7	Scopus	Discard<br>Reasons:<br>Not of interest
369.	Endocannabinoids: A unique opportunity to develop multitarget analgesics<br>Maionea S, Costa B, Di Marzo V<br>Pain 2013<br>154 SUPPL.1:S87-S93 doi:10.1016/j.pain.2013.03.023	Scopus	Discard<br>Reasons:<br>Not of interest
370.	Epidemiology and treatment of pain in multiple sclerosis subjects	Scopus	Discard

	Solaro C<br>Neurological Sciences 2006<br>27SUPPL.4:s291-s293 doi:10.1007/s10072-006-0642-7	Web of Science	Reasons:<br>Not of interest
371.	Epidiolex and potential derived drugs from Cannabis and their future prospectives<br>Rajpal Charu, Kanaujia Akansha, Tomar Pushpa C<br>Medicinal Plants - International Journal of Phytomedicines and Related Industries 2019<br>11(4): 363-367 doi: 10.5958/0975-6892.2019.00047.9	Scopus	Discard<br>Reasons:<br>Not of interest
372.	Evaluate symptomatic therapy in MS: can clinical trials be fine-tuned?<br>Solaro C.<br>Eur J Neurol. 2011<br>18(9):1113-4. doi: 10.1111/j.1468-1331.2011.03425.x.	Pubmed<br>Scopus	Discard<br>Reasons:<br>Not of interest
373.	Evaluating sativex® in neuropathic pain management: A clinical and neurophysiological assessment in multiple sclerosis<br>Russo M, Naro A, Leo A, Sessa E, D'Aleo G, Bramanti P, Calabrò RS<br>Pain Medicine (United States) 2016<br>17(6):1145-1154 doi:10.1093/pm/pnv080	Pubmed<br>Scopus	Discard<br>Reasons:<br>Not of interest
374.	Evaluation of cannabidiol and mesenchymal stem cells in adoptively transferred experimental autoimmune encephalomyelitis. A comparative study.<br>Gonzalez-Garcia C, Sanchez-Lopez AJ, Campos-Ruiz L, Moreno-Torres IP, Coronado MJ, Garcia-Merino JA.<br>Multiple Sclerosis Journal 2015<br>Meeting Abstract: P860 21 432-432 Supplement: 11	Web of Science	Discard<br>Reasons:<br>Not of interest
375.	Evaluation of the safety and tolerability profile of Sativex ®: Is it reassuring enough?<br>Wade D<br>Expert Review of Neurotherapeutics 2012<br>12(4) SUPPL.:9-14 doi:10.1586/ern.12.12	Pubmed<br>Scopus	Discard<br>Reasons:<br>Not of interest
376.	Evaluation of the tolerability and efficacy of Sativex in multiple sclerosis<br>Moreno Torres I, Sanchez AJ, Garcia-Merino A.<br>Expert Rev Neurother. 2014<br>14(11):1243-50. doi: 10.1586/14737175.2014.971758.	Pubmed<br>Scopus<br>Web of Science	Discard<br>Reasons:<br>Not of interest
377.	Evidence for the efficacy and effectiveness of THC-CBD oromucosal spray in symptom management of patients with spasticity due to multiple sclerosis.<br>Zettl UK, Rommer P, Hipp P, Patejdl R.<br>Ther Adv Neurol Disord. 2016<br>9(1):9-30. doi: 10.1177/1756285615612659.	Pubmed<br>Scopus<br>Web of Science	Discard<br>Reasons:<br>Not of interest
378.	Evidence for the use of "medical marijuana" in psychiatric and neurologic disorders<br>Noel C.	Pubmed	Discard<br>Reasons:

	Ment Health Clin. 2018<br>7(1):29-38. doi: 10.9740/mhc.2017.01.029.		Not of interest
379.	Evolution of multiple sclerosis spasticity-associated symptoms: latest data<br>Flachenecker P.<br>Neurodegener Dis Manag. 2016<br>6(6s):9-12. doi: 10.2217/nmt-2016-0047	Pubmed<br>Scopus<br>Web of Science	Discard<br>Reasons:<br>Not of interest
380.	Examining the roles of cannabinoids in pain and other therapeutic indications: a review.<br>Turcotte D, Le Dorze JA, Esfahani F, Frost E, Gomori A, Namaka M<br>EXPERT OPINION ON PHARMACOTHERAPY 2010<br>11(1): 17-31 doi: 10.1517/14656560903413534	Web of Science	Discard<br>Reasons:<br>Not of interest
381.	Exploring cannabis use by patients with multiple sclerosis in a state where cannabis is legal<br>Weinkle L, Domen CH, Shelton I, Sillau S, Nair K, Alvarez E.<br>Mult Scler Relat Disord. 2019<br>27:383-390. doi: 10.1016/j.msard.2018.11.022.	Pubmed<br>Scopus<br>Web of Science	Discard<br>Reasons:<br>Not of interest
382.	Expression and functions of μ-opioid receptors and cannabinoid receptors type 1 in T lymphocytes<br>Kraus J<br>Annals of the New York Academy of Sciences 2012<br>1261(1):1-6 doi:10.1111/j.1749-6632.2012.06524.x	Scopus	Discard<br>Reasons:<br>Not of interest
383.	First-in-human Study With EHP-101 Oral Solution Of A Synthetic Cannabidiol Derivative Enables The Initiation Of A Phase II Study In Multiple Sclerosis<br>Rolland A<br>Multiple Sclerosis Journal 2020<br>26: (1S) 16–89	Web of Science	Discard<br>Reasons:<br>Not of interest
384.	From anecdotal evidence of cannabinoids in multiple sclerosis to emerging new therapeutical approaches<br>Bifulco M, Laezza C, Malfitano AM<br>Multiple Sclerosis 2007<br>13(1):133-134 doi:10.1177/1352458506071303	Scopus	Discard<br>Reasons:<br>Not of interest
385.	From cannabis to endocannabinoids in multiple sclerosis: A paradigm of central nervous system autoimmune diseases<br>Malfitano AM., Matarese G, Bifulco M<br>Current Drug Targets: CNS and Neurological Disorders 2005<br>4(6):667-675 doi:10.2174/156800705774933087	Scopus	Discard<br>Reasons:<br>Not of interest
386.	From cannabis to the endocannabinoid system: Refocussing attention on potential clinical benefits	Scopus	Discard<br>Reasons:

	Youssef FF, Irving AJ<br>West Indian Medical Journal 2012<br>61(3):2841 doi:10.7727/wimj.2010.058		Not of interest
387.	From endocannabinoid profiling to 'endocannabinoid therapeutics'<br>Ligresti A., Petrosino S, Di Marzo V<br>Current Opinion in Chemical Biology 2009<br>13(3):321-331 doi:10.1016/j.cbpa.2009.04.615	Scopus	Discard<br>Reasons:<br>Not of interest
388.	From phytocannabinoids to cannabinoid receptors and endocannabinoids: Pleiotropic physiological and pathological roles through complex pharmacology<br>Ligresti A, De Petrocellis L, Di Marzo V<br>Physiological Reviews 2016<br>96(4):1593-1659 doi:10.1152/physrev.00002.2016	Scopus	Discard<br>Reasons:<br>Not of interest
389.	Functional role of cannabinoid receptors in urinary bladder<br>Tyagi P, Tyagi V, Yoshimura N, Chancellor M<br>Indian Journal of Urology 2010<br>26(1):26-35 doi:10.4103/0970-1591.60440	Scopus	Discard<br>Reasons:<br>Not of interest
390.	Fungal biotransformation of cannabinoids: Potential for new effective drugs<br>Saxena S<br>Current Opinion in Drug Discovery and Development 2009<br>12(2):305-312	Scopus	Discard<br>Reasons:<br>Not of interest
391.	Gait disorders in multiple sclerosis<br>Bethoux F<br>CONTINUUM Lifelong Learning in Neurology 2013<br>19(4):1007-1022 doi:10.1212/01.CON.0000433286.92596.d5	Scopus	Discard<br>Reasons:<br>Not of interest
392.	Gateways to Clinical Trials<br>Bayés M, Rabasseda X, Prous JR<br>Methods and Findings in Experimental and Clinical Pharmacology 2006<br>28(1):31-63	Scopus	Discard<br>Reasons:<br>Not of interest
393.	Gateways to clinical trials<br>Bayés M, Rabasseda X, Prous JR<br>Methods and Findings in Experimental and Clinical Pharmacology 2007<br>29(3):231-245	Scopus	Discard<br>Reasons:<br>Not of interest
394.	Glial cell AMPA Receptors in nervous system health, injury and disease<br>Ceprian M, Fulton D<br>International Journal of Molecular Sciences 2019<br>20(10):2450 doi:10.3390/ijms20102450	Scopus	Discard<br>Reasons:<br>Not of interest

395.	Growing medicine: Small-scale cannabis cultivation for medical purposes in six different countries<br>Hakkarainen P, Asmussen Frank V, Barratt MJ, Vibeke Dahl H, Decorte T, Karjalainen K, Lenton S, Potter G, Werse B<br>International Journal of Drug Policy 2015<br>26(3):250-256 doi:10.1016/j.drugpo.2014.07.005	Scopus	Discard<br>Reasons:<br>Not of interest
396.	GW-1000. GW Pharmaceuticals<br>Smith PF.<br>Curr Opin Investig Drugs. 2004<br>5(7):748-54	Pubmed	Discard<br>Reasons:<br>Not of interest
397.	Health Authorities Data Collection of THC:CBD Oromucosal Spray (L'Agenzia Italiana del Farmaco Web Registry): Figures after 1.5 Years<br>Patti F.<br>Eur Neurol. 2016<br>75 Suppl 1:9-12. doi: 10.1159/000444236.	Pubmed<br>Scopus	Discard<br>Reasons:<br>Not of interest
398.	Heat Exposure of Cannabis sativa Extracts Affects the Pharmacokinetic and Metabolic Profile in Healthy Male Subjects.<br>Eichler M, Spinedi L, Unfer-Grauwiler S, Bodmer M, Surber C, Luedi M, Drewe J<br>PLANTA MEDICA 2012<br>78(7): 686-691 doi: 10.1055/s-0031-1298334	Web of Science	Discard<br>Reasons:<br>Not of interest
399.	High potency cannabis: A risk factor for dependence, poor psychosocial outcomes, and psychosis<br>Hall W, Degenhardt L<br>BMJ (Online) 2015<br>350:h1205	Scopus	Discard<br>Reasons:<br>Not of interest
400.	Historical overview of the rationale for the pharmacological use of prolonged-release fampridine in multiple sclerosis<br>Fernandez O, Berger T, Hartung HP, Putzki N<br>Expert Review of Clinical Pharmacology 2012<br>5(6):5 doi:10.1586/ecp.12.59	Scopus	Discard<br>Reasons:<br>Not of interest
401.	Holmes tremor caused by a natalizumab-related progressive multifocal leukoencephalopathy: a case report and brief review of the literature<br>Magistrelli L, Vecchio D, Naldi P, Comi C, Cantello R<br>Neurological Sciences 2019<br>40(9):1943-1945 doi:10.1007/s10072-019-03779-6	Scopus	Discard<br>Reasons:<br>Not of interest
402.	HU-446 and HU-465, Derivatives of the Non-psychoactive Cannabinoid Cannabidiol, Decrease the Activation of Encephalitogenic T Cells	Pubmed<br>Scopus	Discard<br>Reasons:

	Kozela E, Haj C, HanuÅ¡ L, Chourasia M, Shurki A, Juknat A, Kaushansky N, Mechoulam R, Vogel Z.<br>Chem Biol Drug Des. 2016<br>87(1):143-53. doi: 10.1111/cbdd.12637	Web of Science	Not of interest
403.	Human complication of the foot and mouth crisis.<br>Harkness KAC, Staunton TG<br>JOURNAL OF NEUROLOGY NEUROSURGERY AND PSYCHIATRY 2002<br>72(1): 139	Web of Science	Discard<br>Reasons:<br>Not of interest
404.	Hypoxia mimetic activity of VCE-004.8, a cannabidiol quinone derivative: implications for multiple sclerosis therapy<br>Navarrete C, Carrillo-Salinas F, Palomares B, Mecha M, Jiménez-Jiménez C, Mestre L, Feliú A, Bellido ML, Fiebich BL, Appendino G, Calzado MA, Guaza C, Muñoz E.<br>J Neuroinflammation. 2018<br>15(1):64. doi: 10.1186/s12974-018-1103-y	Pubmed<br>Scopus<br>Web of Science	Discard<br>Reasons:<br>Not of interest
405.	IACM 2nd Conference on Cannabinoids in Medicine<br>Grotenhermen F, Müller-Vahl K<br>Expert Opinion on Pharmacotherapy 2003<br>4(12):2367-2371 doi:10.1517/14656566.4.12.2367	Scopus	Discard<br>Reasons:<br>Not of interest
406.	Identification of cytochrome P450 enzymes responsible for metabolism of cannabidiol by human liver microsomes.<br>Jiang RR, Yamaori S, Takeda S, Yamamoto I, Watanabe K<br>Life Sciences 2011<br>89(5-6): 165-170 doi: 10.1016/j.lfs.2011.05.018	Web of Science	Discard<br>Reasons:<br>Not of interest
407.	Immune Responses Regulated by Cannabidiol<br>Nichols JM, Kaplan BLF<br>Cannabis Cannabinoid Res. 2020<br>5(1): 12–31 doi: 10.1089/can.2018.0073	Pubmed<br>Scopus	Discard<br>Reasons:<br>Not of interest
408.	Immune system modulation in the central nervous system; a possible role for endocannabinoids<br>Abd-Allah ARA<br>Saudi Pharmaceutical Journal 2007<br>15(1):1-15	Scopus	Discard<br>Reasons:<br>Not of interest
409.	Immunomodulatory effects of orally administered cannabinoids in multiple sclerosis.<br>Killestein J, Hoogervorst ELJ, Reif M, Blauw B, Smits M, Uitdehaag BMJ, Nagelkerlen L, Polman CH<br>Journal of Neuroimmunology 2003<br>137(1-2): 140-143 doi: 10.1016/S0165-5728(03)00045-6	Scopus<br>Web of Science	Keep

410.	Immunosuppressive Effects of Cannabidiol in Mild and Moderate Disease States of Experimental Autoimmune Encephalomyelitis.<br>Nichols JM, Gilfeather K, Yray G, Kummari E, Dhital S, Kaplan BLF<br>Journal of Neuroimmune Pharmacology 2017<br>Meeting Abstract: W41 12: S36-S36 Supplement: 1	Web of Science	Discard<br>Reasons:<br>Not of interest.
411.	Impact of Sativex(®) on quality of life and activities of daily living in patients with multiple sclerosis spasticity<br>Arroyo R, Vila C, Dechant KL.<br>J Comp Eff Res. 2014<br>3(4):435-44. doi: 10.2217/cer.14.30. Review	Pubmed<br>Scopus	Discard<br>Reasons:<br>Not of interest
412.	IMPORTANCE OF CANNABINOIDS IN THE FUNCTIONING OF THE CENTRAL NERVOUS SYSTEM.<br>Wroblewska-Luczka P, Florek-Luszczki M, Luszczki JJ<br>HEALTH PROBLEMS OF CIVILIZATION 2018<br>12(3): 223-230 DOI: 10.5114/hpc.2018.75688	Web of Science	Discard<br>Reasons:<br>Not of interest
413.	Industry update covering April 2018<br>Simpson I<br>Therapeutic Delivery 2018<br>9(9):609-616 doi:10.4155/tde-2018-0039	Scopus	Discard<br>Reasons:<br>Not of interest
414.	Influence of Previous Failed Antispasticity Therapy on the Efficacy and Tolerability of THC:CBD Oromucosal Spray for Multiple Sclerosis Spasticity<br>Haupts M, Vila C, Jonas A, Witte K, Álvarez-Ossorio L.<br>Eur Neurol. 2016<br>75(5-6):236-43. doi: 10.1159/000445943.	Pubmed<br>Scopus	Discard<br>Reasons:<br>Not of interest
415.	Inhibitory effect of standardized cannabis sativa extract and its ingredient cannabidiol on rat and human bladder contractility<br>Capasso R, Aviello G, Borrelli F, Romano B, Ferro M, Castaldo L, Montanaro V, Altieri V, Izzo AA.<br>Urology. 2011<br>77(4):1006.e9-1006.e15. doi: 10.1016/j.urology.2010.12.006.	Pubmed<br>Scopus<br>Web of Science	Discard<br>Reasons:<br>Not of interest
416.	Initial experiences with medicinal extracts of cannabis for chronic pain: results from 34 'N of 1' studies<br>Notcutt W, Price M, Miller R, Newport S, Phillips C, Simmons S, Sansom C.<br>Anaesthesia. 2004<br>59(5):440-52. doi: 10.1111/j.1365-2044.2004.03674.x	Pubmed<br>Scopus<br>Web of Science	Discard<br>Reasons:<br>Not of interest
417.	Integrative therapies for multiple sclerosis<br>Maker-Clark G, Patel S	Scopus	Discard<br>Reasons:

	Disease-a-Month 2013<br>59(8):290-301 doi:10.1016/j.disamonth.2013.03.017		Not of interest
418.	Interaction between the protective effects of cannabidiol and palmitoylethanolamide in experimental model of multiple sclerosis in C57BL/6 mice<br>Rahimi A, Faizi M, Talebi F, Noorbakhsh F, Kahrizi F, Naderi N.<br>Neuroscience. 2015<br>290:279-87. doi: 10.1016/j.neuroscience.2015.01.030.	Pubmed<br>Scopus<br>Web of Science	Keep
419.	Interactions between THC and cannabidiol in mouse models of cannabinoid activity.<br>Varvel SA, Wiley JL, Yang R, Bridgen DT, Long K, Lichtman AH, Martin BR<br>Psychopharmacology 2006<br>186(2): 226-234 doi: 10.1007/s00213-006-0356-9	Web of Science	Discard<br>Reasons:<br>Not of interest
420.	Is cannabidiol a promising substance for new drug development? A review of its potential therapeutic applications<br>Noreen N, Muhammad F, Akhtar B, Azam F, Anwar MI<br>Critical Reviews in Eukaryotic Gene Expression 2018<br>28(1):73-86 doi:10.1615/CritRevEukaryotGeneExpr.2018021528	Scopus<br>Web of Science	Discard<br>Reasons:<br>Not of interest
421.	Is the Clinical Use of Cannabis by Oncology Patients Advisable?<br>Bar-Sela G, Avisar A, Batash R, Schaffer M<br>Current Medicinal Chemistry 2014<br>21(17): 1923-1930 doi: 10.2174/0929867321666140304151323	Web of Science	Discard<br>Reasons:<br>Not of interest
422.	Is the medical use of cannabis a therapeutic option for children?<br>Rieder MJ, Canadian Paediat Soc; Canadian Paediat Soc; Drug Therapy Hazardous Subst Comm.<br>PAEDIATRICS & CHILD HEALTH 2016<br>21(1): 31-34 doi: 10.1093/pch/21.1.31	Web of Science	Discard<br>Reasons:<br>Not of interest
423.	Is There a Legitimate Role for the Therapeutic Use of Cannabinoids for Symptom Management in Chronic Kidney Disease?<br>Davison SN, Davison JS<br>Journal of Pain and Symptom Management 2011<br>41(4): 768-778 doi: 10.1016/j.jpainsymman.2010.06.016	Web of Science	Discard<br>Reasons:<br>Not of interest
424.	Is there a need for weed? The role of cannabinoids in managing neuropathic pain<br>No author name available<br>Drugs and Therapy Perspectives 2009<br>25(4):11-13 doi:10.2165/0042310-200925040-00004	Scopus	Discard<br>Reasons:<br>Not of interest
425.	Is there any clinically relevant cannabinoid-induced analgesia?<br>Kraft B<br>Pharmacology 2012	Scopus	Discard<br>Reasons:<br>Not of interest

	89(5-6):237-246 doi:10.1159/000337376		
426.	Issues for pharmacists when patients use cannabis therapeutically at home<br>Green A<br>Pharmaceutical Journal 2012<br>289(7731):531-532	Scopus	Discard<br>Reasons:<br>Not of interest
427.	Italian Consensus on treatment of spasticity in multiple sclerosis<br>Comi G, Solari A, Leocani L, Centonze D, Otero-Romero S Italian Consensus Group on treatment of spasticity in multiple sclerosis.<br>Eur J Neurol. 2019 doi: 10.1111/ene.14110.	Pubmed<br>Web of Science	Discard<br>Reasons:<br>Not of interest
428.	Key considerations in reimbursement decision-making for multiple sclerosis drugs in Australia<br>Phan YHL, De Abreu Lourenco R, Haas M, van der Linden N.<br>Mult Scler Relat Disord. 2018<br>25:144-149. doi: 10.1016/j.msard.2018.07.020.	Pubmed<br>Scopus<br>Web of Science	Discard<br>Reasons:<br>Not of interest
429.	Lack of effect of cannabis-based treatment on clinical and laboratory measures in multiple sclerosis<br>Centonze D, Mori F, Koch G, Buttari F, Codecà C, Rossi S, Cencioni MT, Bari M, Fiore S, Bernardi G, Battistini L, Maccarrone M.<br>Neurol Sci. 2009<br>30(6):531-4. doi: 10.1007/s10072-009-0136-5	Pubmed<br>Scopus<br>Web of Science	Keep
430.	Latest advances in cannabinoid receptor agonists<br>Thakur GA, Tichkule R, Bajaj S, Makriyannis A<br>Expert Opinion on Therapeutic Patents 2009<br>19(12):1647-1673 doi:10.1517/13543770903436505	Scopus	Discard<br>Reasons:<br>Not of interest
431.	Legalizing marijuana<br>Leyton M<br>Journal of Psychiatry and Neuroscience 2016<br>41(2):75-76 doi:10.1503/jpn.160012	Scopus	Discard<br>Reasons:<br>Not of interest
432.	Lipid sensing G protein-coupled receptors in the CNS<br>Dev KK, Irving AJ<br>Neuropharmacology 2017<br>113:595-596 doi:10.1016/j.neuropharm.2016.10.030	Scopus	Discard<br>Reasons:<br>Not of interest
433.	Long-term assessment of the cognitive effects of nabiximols in patients with multiple sclerosis: A pilot study<br>Alessandria G, Meli R, Infante MT, Vestito L, Capello E, Bandini F.<br>Clin Neurol Neurosurg. 2020<br>196:105990 doi: 10.1016/j.clineuro.2020.105990	Pubmed<br>Scopus	Discard<br>Reasons:<br>Not of interest

434.	Long-Term Data of Efficacy, Safety, and Tolerability in a Real-Life Setting of THC/CBD Oromucosal Spray-Treated Multiple Sclerosis Patients<br>Paolicelli D, Direnzo V, Manni A, D'Onghia M, Tortorella C, Zoccolella S, Di Lecce V, Iaffaldano A, Trojano M.<br>J Clin Pharmacol. 2016<br>56(7):845-51. doi: 10.1002/jcph.670.	Pubmed<br>Scopus<br>Web of Science	Discard<br>Reasons:<br>Not of interest
435.	Long-term effectiveness and safety of nabiximols (tetrahydrocannabinol/cannabidiol oromucosal spray) in clinical practice<br>Flachenecker P, Henze T, Zettl UK.<br>Eur Neurol. 2014<br>72(1-2):95-102. doi: 10.1159/000360285.	Pubmed<br>Scopus<br>Web of Science	Discard<br>Reasons:<br>Not of interest
436.	Lost in the Haze: The Physician's Role in Cannabinoid Prescribing and Advising<br>Am J Med 2020<br>133(1):7-8. doi: 10.1016/j.amjmed.2019.05.049.	Scopus	Discard<br>Reasons:<br>Not of interest
437.	Lower Urinary Tract Dysfunction in Multiple Sclerosis<br>Seth JH, Sahai A, Panicker JN<br>Current Bladder Dysfunction Reports 2012<br>7(2):97-104 doi:10.1007/s11884-012-0129-8	Scopus	Discard<br>Reasons:<br>Not of interest
438.	Management of lower urinary tract dysfunction in multiple sclerosis: A systematic review and Turkish consensus report<br>Çetinel B, Tarcan T, Demirkesen O, Özyurt C, Şen I, Erdoğan S, Siva A<br>Neurourology and Urodynamics 2013<br>32(8):1047-1057 doi:10.1002/nau.22374	Scopus	Discard<br>Reasons:<br>Not of interest
439.	Management of neurogenic bladder in patients with multiple sclerosis<br>Phé V, Chartier-Kastler E, Panicker JN<br>Nature Reviews Urology 2016<br>13(5):275-288 doi:10.1038/nrurol.2016.53	Scopus	Discard<br>Reasons:<br>Not of interest
440.	Management of pain in multiple sclerosis: A pharmacological approach<br>Solaro C, Messmer Uccelli M<br>Nature Reviews Neurology 2011<br>7(9):519-527 doi:10.1038/nrneurol.2011.120	Scopus	Discard<br>Reasons:<br>Not of interest
441.	Managing neuropathic pain with Sativex®: A review of its pros and cons<br>Perez J, Ribera MV<br>Expert Opinion on Pharmacotherapy 2008,<br>9(7):1189-1195 doi:10.1517/14656566.9.7.1189	Scopus	Discard<br>Reasons:<br>Not of interest
442.	Marihuana and cannobinoids as medicaments. [Marihuana i kanabinoidy jako leki.]<br>Tkaczyk M, Florek E, Piekoszewski W	Pubmed<br>Scopus	Discard<br>Reasons:

	Przegląd lekarski 2012<br>69(10):1095-1097		Not of interest
443.	Marihuana: Medicine, addictive substance, or both? A common-sense approach to the place of cannabis in medicine<br>Kalant H<br>CJAM Canadian Journal of Addiction Medicine 2013<br>4(3):4-8	Scopus	Discard<br>Reasons:<br>Not of interest
444.	Marijuana legalization: Impact on physicians and public health<br>Wilkinson ST, Yarnell S, Radhakrishnan R, Ball SA, D'Souza DC<br>Annual Review of Medicine 2016<br>67:453-466 doi:10.1146/annurev-med-050214-013454	Scopus	Discard<br>Reasons:<br>Not of interest
445.	Marijuana use in America: Whither are we bound?<br>Devane CL<br>Pharmacotherapy 2013<br>3(10):1009-1011 doi:10.1002/phar.1363	Scopus	Discard<br>Reasons:<br>Not of interest
446.	Marijuana: Modern medical chimaera<br>Lamarine RJ<br>Journal of Drug Education 2012<br>42(1):1-11 doi:10.2190/DE.42.1.a	Scopus	Discard<br>Reasons:<br>Not of interest
447.	Mechanisms and pharmacology of neuropathic pain in multiple sclerosis<br>Iannitti T, Kerr BJ, Taylor BK<br>Current Topics in Behavioral Neurosciences 2014<br>20:75-97 doi:10.1007/7854_2014_288	Scopus	Discard<br>Reasons:<br>Not of interest
448.	Mechanisms of action of cannabidiol in adoptively transferred experimental autoimmune encephalomyelitis<br>González-García C, Torres IM, García-Hernández R, Campos-Ruíz L, Esparragoza LR, Coronado MJ, Grande AG, García-Merino A, Sánchez López AJ.<br>Exp Neurol. 2017<br>298(Pt A):57-67. doi: 10.1016/j.expneurol.2017.08.017	Pubmed<br>Scopus<br>Web of Science	Keep
449.	Medical cannabinoids<br>McLeod SA, Lemay J-F<br>CMAJ 2017<br>189(30):E995 doi:10.1503/cmaj.161395	Scopus	Discard<br>Reasons:<br>Not of interest
450.	Medical cannabis – The Canadian perspective<br>Ko GD, Bober SL, Mindra S, Moreau JM<br>Journal of Pain Research 2016,<br>9:735-744 doi:10.2147/JPR.S98182	Scopus	Discard<br>Reasons:<br>Not of interest

451.	Medical cannabis and cannabinoids in rheumatology: where are we now?<br>Expert Review of Clinical Immunology 2019<br>15(10):1019-1032 doi:10.1080/1744666X.2019.1665997	Scopus	Discard<br>Reasons:<br>Not of interest
452.	Medical cannabis: Pharmacy focus on treatment options for neurologic conditions<br>Berlekamp D<br>U.S. Pharmacist 2016<br>41(1):45-49	Scopus	Discard<br>Reasons:<br>Not of interest
453.	Medical cannabis: Time for clear thinking<br>Penington DG<br>Medical Journal of Australia 2015<br>202(2):74-76 doi:10.5694/mja14.01573	Scopus	Discard<br>Reasons:<br>Not of interest
454.	Medical cannabis: What practitioners need to know<br>Van Rensburg R, Pillay-Fuentes Lorente V, Blockman M, Moodley K, Wilmshurst JM, Decloedt EH.<br>S Afr Med J. 2020<br>110(3):192-196 doi:10.7196/SAMJ.2020.v110i3.14403	Pubmed<br>Web of Science	Discard<br>Reasons:<br>Not of interest
455.	Medical Marijuana and Chronic Pain: a Review of Basic Science and Clinical Evidence.<br>Jensen B, Chen J, Furnish T, Wallace M<br>CURRENT PAIN AND HEADACHE REPORTS 2015<br>19(10):50 DOI: 10.1007/s11916-015-0524-x	Web of Science	Discard<br>Reasons:<br>Not of interest
456.	Medical marijuana and pain management<br>Temple LM<br>Disease-a-Month 2016<br>62(9):346-352 doi:10.1016/j.disamonth.2016.05.014	Scopus	Discard<br>Reasons:<br>Not of interest
457.	Medical marijuana for cancer<br>Kramer JL<br>CA Cancer Journal for Clinicians 2015<br>65(2):110-122 doi:10.3322/caac.21260	Pubmed<br>Scopus<br>Web of Science	Discard<br>Reasons:<br>Not of interest
458.	Medical marijuana for the treatment of vismodegib-related muscle spasm<br>Yuan JT, Tello TL, Hultman C, Barker CA, Arron ST, Yom SS.<br>JAAD Case Rep. 2017<br>3(5):438-440. doi: 10.1016/j.jdcr.2017.06.012.	Pubmed	Discard<br>Reasons:<br>Not of interest
459.	Medical marijuana for treatment of chronic pain and other medical and psychiatric problems: A clinical review<br>Hill KP<br>JAMA - Journal of the American Medical Association 2015<br>313(24):2474-2483 doi:10.1001/jama.2015.6199	Scopus	Discard<br>Reasons:<br>Not of interest

460.	Medical marijuana in neurology<br>Benbadis SR, Sanchez-Ramos J, Bozorg A, Giarratano M, Kalidas K, Katzin L, Robertson D, Vu T, Smith A, Zesiewicz T<br>Expert Review of Neurotherapeutics 2014<br>14(12):1453-1465 doi:10.1586/14737175.2014.985209	Pubmed<br>Scopus<br>Web of Science	Discard<br>Reasons:<br>Not of interest
461.	Medical Marijuana is legal in most states, but physicians have little evidence to guide them<br>JAMA - Journal of the American Medical Association 2017<br>317(16):1611-1613 doi:10.1001/jama.2017.0813	Scopus	Discard<br>Reasons:<br>Not of interest
462.	Medical marijuana patient counseling points for health care professionals based on trends in the medical uses, efficacy, and adverse effects of cannabis-based pharmaceutical drugs<br>Parmar JR, Forrest BD, Freeman RA.<br>Res Social Adm Pharm. 2016<br>12(4):638-54. doi: 10.1016/j.sapharm.2015.09.002.	Pubmed<br>Scopus<br>Web of Science	Discard<br>Reasons:<br>Not of interest
463.	Medical marijuana potential and pitfalls<br>Zanni GR<br>Pharmacy Times 2013<br>79:3	Scopus	Discard<br>Reasons:<br>Not of interest
464.	Medical marijuana<br>Gloss DS, Maa EH<br>Neurology: Clinical Practice 2015<br>5(4):281-284 doi:10.1212/CPJ.0000000000000159	Scopus	Discard<br>Reasons:<br>Not of interest
465.	Medical marijuana: Clearing away the smoke<br>Grant I, Hampton Atkinson J, Gouaux B, Wilsey B<br>Open Neurology Journal 2012<br>6(1):18-25 doi:10.2174/1874205X01206010018	Scopus	Discard<br>Reasons:<br>Not of interest
466.	Medical marijuana: Do the benefits outweigh the risks?<br>Gupta S, Phalen T, Gupta S<br>Current Psychiatry 2018<br>17(1):34-41	Scopus	Discard<br>Reasons:<br>Not of interest
467.	Medical Use of Cannabinoids<br>Fraguas-Sánchez AI, Torres-Suárez AI<br>Drugs 2018<br>78(16):1665-1703 doi:10.1007/s40265-018-0996-1	Pubmed<br>Scopus<br>Web of Science	Discard<br>Reasons:<br>Not of interest
468.	Medical use of cannabis products Lessons to be learned from Israel and Canada.<br>Ablin J, Ste-Marie PA, Schafer M, Hauser W, Fitzcharles MA<br>SCHMERZ 2016<br>30(1): 3-13 doi: 10.1007/s00482-015-0083-4	Pubmed<br>Scopus<br>Web of Science	Discard<br>Reasons:<br>Not of interest

469.	Medical use of cannabis. Cannabidiol: A new light for schizophrenia?<br>Deiana S<br>Drug Testing and Analysis 2013<br>5(1):46-51 doi:10.1002/dta.1425	Scopus	Discard<br>Reasons:<br>Not of interest
470.	Medical use of cannabis: An addiction medicine perspective<br>Cook J, Lloyd-Jones DM, Ogden E, Bonomo Y<br>Internal Medicine Journal 2015,<br>45(6):677-680 doi:10.1111/imj.12761	Scopus	Discard<br>Reasons:<br>Not of interest
471.	Medical use of marijuana: Truth in evidence<br>Naguib M, Foss JF<br>Anesthesia and Analgesia 2015<br>121(5):1124-1127 doi:10.1213/ANE.0000000000000928	Scopus	Discard<br>Reasons:<br>Not of interest
472.	Medicinal cannabis [Medicinale cannabis]<br>Van Der Meersch H, Verschuere AP, Bottriaux F<br>Farmaceutisch Tijdschrift voor Belgie 2006<br>83(3):3-12	Scopus	Discard<br>Reasons:<br>Not of interest
473.	Medicinal cannabis extracts for the treatment of multiple sclerosis<br>Smith PF.<br>Curr Opin Investig Drugs. 2004<br>5(7):727-30	Pubmed<br>Scopus	Discard<br>Reasons:<br>Not of interest
474.	Medicinal cannabis in oncology practice: Still a bridge too far?<br>De Jong FA, Engels FK, Mathijssen RHJ, Van Zuylen L, Verweij J, Peters RPH, Sparreboom A<br>Journal of Clinical Oncology 2005<br>23(13):2886-2891 doi:10.1200/JCO.2005.04.150	Scopus	Discard<br>Reasons:<br>Not of interest
475.	Medicinal cannabis<br>Murnion B<br>Australian Prescriber 2015<br>38(6):212-215 doi:10.18773/austprescr.2015.072	Scopus<br>Web of Science	Discard<br>Reasons:<br>Not of interest
476.	Medicinal cannabis: History, pharmacology, and implications for the acute care setting<br>Bridgeman MB, Abazia DT<br>P and T 2017<br>42(3):180-188	Scopus	Discard<br>Reasons:<br>Not of interest
477.	Medicinal cannabis: is Delta(9)-tetrahydrocannabinol necessary for all its effects?.<br>Wilkinson JD, Whalley BJ, Baker D, Pryce G, Constanti A, Gibbons S, Williamson EM<br>JOURNAL OF PHARMACY AND PHARMACOLOGY 2003<br>55(12): 1687-1694 DOI: 10.1211/0022357022304	Web of Science	Discard<br>Reasons:<br>Not of interest

478.	Medicinal plants and dementia therapy: Herbal hopes for brain aging?<br>Perry E, Howes M-JR<br>CNS Neuroscience and Therapeutics 2011<br>17(6):683-698 doi:10.1111/j.1755-5949.2010.00202.x	Scopus	Discard<br>Reasons:<br>Not of interest
479.	Medicinal use of cannabis based products and cannabinoids<br>Freeman TP, Hindocha C, Green SF, Bloomfield MAP<br>BMJ (Online) 2019<br>365:l1141 doi:10.1136/bmj.l1141	Scopus	Discard<br>Reasons:<br>Not of interest
480.	Medicinal use of cannabis in the United States: Historical perspectives, current trends, and future directions<br>Aggarwal SK, Carter GT, Sullivan MD, ZumBrunnen C, Morrill R, Mayer JD<br>Journal of Opioid Management 2009<br>5(3):153-168	Scopus	Discard<br>Reasons:<br>Not of interest
481.	Medicinal Uses of Marijuana and Cannabinoids<br>Grotenhermen F, Muller-Vahl K<br>Critical Reviews in Plant Sciences 2016<br>35(5-6):378-405 doi:10.1080/07352689.2016.1265360	Scopus<br>Web of Science	Discard<br>Reasons:<br>Not of interest
482.	Medium and long term efficacy of nabiximols for the treatment of multiple sclerosis related spasticity: an Italian monocentric study.<br>Ferre L, Nuara A, Pavan G, Radaelli M, Moiola L, Rodegher M, Colombo B, Sarmiento IJK, Martinelli V, Leocani L, Comi G, Boneschi FM, Esposito F<br>MULTIPLE SCLEROSIS JOURNAL 2015<br>Meeting Abstract: EP1393 21: 728-729 Supplement: 11	Web of Science	Discard<br>Reasons:<br>Not of interest
483.	Meta-analysis of cannabis based treatments for neuropathic and multiple sclerosis-related pain<br>Iskedjian M, Bereza B, Gordon A, Piwko C, Einarson TR.<br>Curr Med Res Opin. 2007<br>23(1):17-24 doi: 10.1185/030079906X158066	Pubmed<br>Scopus<br>Web of Science	Discard<br>Reasons:<br>Not of interest
484.	Meta-analysis of the efficacy and safety of Sativex (nabiximols), on spasticity in people with multiple sclerosis<br>Wade DT, Collin C, Stott C, Duncombe P.<br>Mult Scler. 2010 Jun 16(6):707-14. doi: 10.1177/1352458510367462	Pubmed<br>Scopus<br>Web of Science	Discard<br>Reasons:<br>Not of interest
485.	Microarray and Pathway Analysis Reveal Distinct Mechanisms Underlying Cannabinoid-Mediated Modulation of LPS-Induced Activation of BV-2 Microglial Cells.<br>Juknat A, Pietr M, Kozela E, Rimmerman N, Levy R, Gao FY, Coppola G, Geschwind D, Vogel Z<br>PLOS ONE 2013	Web of Science	Discard<br>Reasons:<br>Not of interest

	8(4): e61462 DOI: 10.1371/journal.pone.0061462		
486.	Modulating the endocannabinoid system in human health and disease - Successes and failures<br>Pacher P, Kunos G<br>FEBS Journal 2013<br>280(9):1918-1943 doi:10.1111/febs.12260	Scopus	Discard<br>Reasons:<br>Not of interest
487.	Modulation of Astrocyte Activity by Cannabidiol, a Nonpsychoactive Cannabinoid.<br>Kozela E, Juknat A, Vogel Z<br>INTERNATIONAL JOURNAL OF MOLECULAR SCIENCES 2017<br>18(8) Article Number: 1669 DOI: 10.3390/ijms18081669	Pubmed<br>Scopus<br>Web of Science	Discard<br>Reasons:<br>Not of interest
488.	Monotherapy with nabiximols in multiple sclerosis-induced spasticity.<br>Koehler J, Gorodetzky H, Pollmann W, Meier M, Feneberg W<br>MULTIPLE SCLEROSIS JOURNAL 2013<br>19(11): 282-282 Supplement: S	Web of Science	Discard<br>Reasons:<br>Not of interest
489.	Multicenter, Double-Blind, Randomized, Placebo-Controlled, Parallel-Group Study of the Efficacy, Safety, and Tolerability of THC:CBD Extract and THC Extract in Patients with Intractable Cancer-Related Pain.<br>Johnson JR, Burnell-Nugent M, Lossignol D, Ganae-Motan ED, Potts R, Fallon MT<br>JOURNAL OF PAIN AND SYMPTOM MANAGEMENT 2010<br>39(2): 167-179 DOI: 10.1016/j.jpainsymman.2009.06.008	Web of Science	Discard<br>Reasons:<br>Not of interest
490.	Multiple sclerosis and pain<br>Nick ST, Robertsa C, Billiodeaux S, Davis DE, Zamanifekri B, Sahraian, MA, Alekseeva N, Munjampalli S, Roberts J, Minagar A<br>Neurological Research 2012<br>34(9):829-841 doi:10.1179/1743132812Y.0000000082	Scopus	Discard<br>Reasons:<br>Not of interest
491.	Multiple sclerosis and the blood-central nervous system barrier<br>Palmer AM.<br>Cardiovascular Psychiatry and Neurology 2013<br>2013:530356 doi:10.1155/2013/530356	Pubmed<br>Scopus	Discard<br>Reasons:<br>Not of interest
492.	Multiple sclerosis guideline production takes off<br>Maurice J<br>The Lancet 2014<br>384(9958):1914-1915 doi:10.1016/S0140-6736(14)62265-0	Scopus	Discard<br>Reasons:<br>Not of interest
493.	Multiple sclerosis spasticity management-key publications<br>Gold R<br>Expert Review of Neurotherapeutics 2013<br>13(12)SUPPL.:47-48 doi:10.1586/14737175.2013.865875	Scopus	Discard<br>Reasons:<br>Not of interest

494.	Multiple sclerosis spasticity symptoms management. Endocannabinoid system modulator data beyond clinical trials. Foreword<br>Trojano M.<br>Expert Rev Neurother. 2013<br>13(3 Suppl 1):1. doi: 10.1586/ern.13.2. No abstract available.	Pubmed<br>Scopus	Discard<br>Reasons:<br>Not of interest
495.	Multiple sclerosis symptoms and spasticity management: new data<br>Izquierdo G<br>Neurodegenerative disease management 2017<br>7(6):7-11 doi:10.2217/nmt-2017-0034	Pubmed<br>Scopus<br>Web of Science	Discard<br>Reasons:<br>Not of interest
496.	Multiple sclerosis treatment options<br>Hedley L<br>Pharmaceutical Journal 2012<br>288(7694):247-250	Scopus	Discard<br>Reasons:<br>Not of interest
497.	Multiple sclerosis<br>Nafee T, Watanabe R, Fregni F<br>Neuromethods 2018<br>138(263):295 doi:10.1007/978-1-4939-7880-9_8	Scopus	Discard<br>Reasons:<br>Not of interest
498.	Multiple sclerosis: New insights and trends<br>Koriem KMM<br>Asian Pacific Journal of Tropical Biomedicine 2016<br>6(5):429-440 doi:10.1016/j.apjtb.2016.03.009	Scopus	Discard<br>Reasons:<br>Not of interest
499.	Multiple sclerosis: Oromucosal spray relieves multiple sclerosis-induced spasms [Multiple sklerose: Oromukosalspray lindert ms-induzierte spasmen]<br>Bischoff M<br>Neurologie und Rehabilitation 2012<br>18(3):197	Scopus	Discard<br>Reasons:<br>Not of interest
500.	MyD88-dependent and -independent signalling via TLR3 and TLR4 are differentially modulated by Delta(9)-tetrahydrocannabinol and cannabidiol in human macrophages<br>Fitzpatrick J-M, Minogue E, Curham L, Tyrrell H, Gavigan P, Hind W, Downer EJ<br>J Neuroimmunol 2020<br>343:577217. doi: 10.1016/j.jneuroim.2020.577217	Web of Science	Discard<br>Reasons:<br>Not of interest
501.	Multiple sclerosis-related central pain disorders<br>Nurmikko TJ, Gupta S, Maclver K<br>Current Pain and Headache Reports 2010<br>14(3):189-195 doi:10.1007/s11916-010-0108-8	Scopus	Discard<br>Reasons:<br>Not of interest
502.	Nabilone increases choreatic movements in Huntington's disease.<br>Muller-Vahl KR, Schneider U, Emrich HM	Web of Science	Discard<br>Reasons:

	MOVEMENT DISORDERS 1999<br>14(6): 1038-1040 doi: 10.1002/1531-8257(199911)14:6<1038::AID-MDS1024>3.0.CO;2-7		Not of interest
503.	Nabiximols (THC/CBD Oromucosal Spray, Sativex (R)) in Clinical Practice - Results of a Multicenter, Non-Interventional Study (MOVE 2) in Patients with Multiple Sclerosis Spasticity.<br>Flachenecker P, Henze T, Zettl UK<br>EUROPEAN NEUROLOGY 2014<br>71(5-6):271-279 DOI: 10.1159/000357427	Pubmed<br>Scopus<br>Web of Science	Discard<br>Reasons:<br>Not of interest
504.	Nabiximols as an agonist replacement therapy during cannabis withdrawal: a randomized clinical trial<br>Allsop DJ, Copeland J, Lintzeris N, Dunlop AJ, Montebello M, Sadler C, Rivas GR, Holland RM, Muhleisen P, Norberg MM, Booth J, McGregor IS.<br>JAMA Psychiatry. 2014<br>71(3):281-91. doi: 10.1001/jamapsychiatry.2013.3947	Pubmed<br>Scopus<br>Web of Science	Discard<br>Reasons:<br>Not of interest
505.	Nabiximols discontinuation rate in a large population of patients with multiple sclerosis: a 18-month multicentre study<br>Chisari CG, Solaro C, Annunziata P, Bergamaschi R, Bianco A, Bonavita S, Brescia Morra V, Bruno Bossio R, Capello E, Castelli L, Cavalla P, Costantino G, Centonze D, Cottone S, Danni MC, Esposito F, Gajofatto A, Gasperini C, Guareschi A, Lanzillo R, Lus G, Maniscalco GT, Matta M, Paolicelli D, Petrucci L, Pontecorvo S, Righini I, Rovaris M, Sessa E, Spinicci G, Spitaleri D, Valentino P, Zaffaroni M, Zappia M, Patti F.<br>J Neurol Neurosurg Psychiatry. 2020<br>doi: 10.1136/jnnp-2019-322480	Pubmed	Discard<br>Reasons:<br>Not of interest
506.	Nabiximols for multiple sclerosis<br>[No authors listed]","Aust Prescr. 2018<br>41(6):203-204. doi: 10.18773/austprescr.2018.066.	Pubmed	Discard<br>Reasons:<br>Not of interest
507.	Nabiximols for Opioid-Treated Cancer Patients With Poorly-Controlled Chronic Pain: A Randomized, Placebo-Controlled, Graded-Dose Trial.<br>Portenoy RK, Ganae-Motan ED, Allende S, Yanagihara R, Shaiova L, Weinstein S, McQuade R, Wright S, Fallon MT<br>JOURNAL OF PAIN 2012<br>13(5): 438-449 DOI: 10.1016/j.jpain.2012.01.003	Web of Science	Discard<br>Reasons:<br>Not of interest
508.	Nabiximols plus robotic assisted gait training in improving motor performances in people with Multiple Sclerosis<br>Calabrò RS, Russo M, Naro A, Ciurleo R, D'Aleo G, Rifici C, Balletta T, La Via C, Destro M, Bramanti P, Sessa E	Pubmed<br>Scopus<br>Web of Science	Discard<br>Reasons:<br>Not of interest

	Mult Scler Relat Disord. 2020<br>43, 102177 doi: 10.1016/j.msard.2020.102177		
509.	Nabiximols in the treatment of spasticity, pain and urinary symptoms due to multiple sclerosis<br>Podda G, Constantinescu CS.<br>Expert Opin Biol Ther. 2012<br>12(11):1517-31. doi: 10.1517/14712598.2012.721765.	Pubmed<br>Scopus<br>Web of Science	Discard<br>Reasons:<br>Not of interest
510.	N-arachidonoyl glycine, an abundant endogenous lipid, potently drives directed cellular migration through GPR18, the putative abnormal cannabidiol receptor.<br>McHugh D, Hu SSJ, Rimmerman N, Juknat A, Vogel Z, Walker JM, Bradshaw HB<br>BMC NEUROSCIENCE 2010<br>11 Article Number: 44 DOI: 10.1186/1471-2202-11-44	Web of Science	Discard<br>Reasons:<br>Not of interest
511.	Nasal administration of drugs as a new non-invasive strategy for efficient treatment of multiple sclerosis<br>Duchi S, Ovadia H, Touitou E.<br>J Neuroimmunol. 2013<br>258(1-2):32-40. doi: 10.1016/j.jneuroim.2013.02.013	Pubmed<br>Scopus<br>Web of Science	Keep
512.	Natural products as a potential modulator of microglial polarization in neurodegenerative diseases<br>Jin X, Liu M-Y, Zhang D-F, Zhong X, Du K, Qian P, Gao H, Wei M-J<br>Pharmacological Research 2019<br>145:104253 doi:10.1016/j.phrs.2019.104253	Scopus	Discard<br>Reasons:<br>Not of interest
513.	Natural products: Potential therapeutic agents in multiple sclerosis<br>Yu S, Liu M, Hu K<br>International Immunopharmacology 2019<br>67(87):97 doi:10.1016/j.intimp.2018.11.036	Scopus	Discard<br>Reasons:<br>Not of interest
514.	Naturally occurring and related synthetic cannabinoids and their potential therapeutic applications<br>Galal AM, Slade D, Gul W, El-Alfy AT, Ferreira D, Elsohly MA<br>Recent Patents on CNS Drug Discovery 2009<br>4(2):112-136 doi:10.2174/157488909788453031	Scopus	Discard<br>Reasons:<br>Not of interest
515.	Neurological aspects of medical use of cannabidiol<br>Mannucci C, Navarra M, Calapai F, Ventura-Spagnolo E, Busardò FP, Cas RD, Ippolito FM, Calapai G<br>CNS and Neurological Disorders - Drug Targets 2017<br>16(5):541-553 doi:10.2174/1871527316666170413114210	Pubmed<br>Scopus<br>Web of Science	Discard<br>Reasons:<br>Not of interest
516.	Neurological rehabilitation and the management of spasticity	Scopus	Discard

	Stevenson V, Playford D<br>Medicine (United Kingdom) 2012<br>40(9):513-517 doi:10.1016/j.mpmed.2012.06.008		Reasons:<br>Not of interest
517.	Neuronal preservation and reactive gliosis attenuation following neonatal sciatic nerve axotomy by a fluorinated cannabidiol derivative.<br>Perez M, Cartarozzi LP, Chiarotto GB, de Oliveira SA, Guimaraes FS, de Oliveira ALR<br>NEUROPHARMACOLOGY 2018<br>140:201-208 DOI: 10.1016/j.neuropharm.2018.08.009	Web of Science	Discard<br>Reasons:<br>Not of interest
518.	Neurophysiological and subjective profile of marijuana with varying concentrations of cannabinoids.<br>Ilan AB, Gevins A, Coleman M, ElSohly MA, de Wit H<br>BEHAVIOURAL PHARMACOLOGY 2005<br>16(5-6): 487-497 DOI: 10.1097/00008877-200509000-00023	Web of Science	Discard<br>Reasons:<br>Not of interest
519.	Neuroprotection in Experimental Autoimmune Encephalomyelitis and Progressive Multiple Sclerosis by Cannabis-Based Cannabinoids<br>Pryce G, Riddall DR, Selwood DL, Giovannoni G, Baker D.<br>J Neuroimmune Pharmacol. 2015<br>10(2):281-92. doi: 10.1007/s11481-014-9575-8.	Pubmed<br>Scopus<br>Web of Science	Discard<br>Reasons:<br>Not of interest
520.	Neuroprotective agents: Cannabinoids<br>Sánchez AJ, García-Merino A<br>Clinical Immunology 2012,<br>142(1):57-67 doi:10.1016/j.clim.2011.02.010	Scopus	Discard<br>Reasons:<br>Not of interest
521.	Neuroprotective effect of cannabidiol on NTF-3 and IGF-1 genes expression<br>Miandashti N, Safaralizadeh R, Hosseinpourfeizi MA, Mahdavi M<br>Indian Journal of Traditional Knowledge 2019<br>18(4):739-743	Scopus	Discard<br>Reasons:<br>Not of interest
522.	Neuroprotective Effect of Cannabinoids in Neurodegenerative Diseases.<br>Suero-Garcia C, Martin-Banderas L, Holgado MA<br>ARS PHARMACEUTICA 2015<br>56(2): 77-87	Web of Science	Discard<br>Reasons:<br>Not of interest
523.	Neuroprotective effect of cannabidiol on NTF-3 and IGF-1 genes expression<br>Safaralizadeh R, Miandashti N, Hosseinpourfeizi MA, Mahdavi M<br>Indian Journal of Traditional Knowledge (IJTK) 2019<br>18(4):739-743	Web of Science	Discard<br>Reasons:<br>Not of interest
524.	New and Emerging Medications for Treatment of Pediatric Epilepsy<br>M Scott Perry<br>Pediatr Neurol 2020	Scopus	Discard<br>Reasons:<br>Not of interest

	107:24-27. doi: 10.1016/j.pediatrneurol.2019.11.008		
525.	New approaches to pain management<br>Wilcock A<br>Progress in Palliative Care 2001<br>9(3):100-101 doi:10.1080/09699260.2001.11746914	Scopus	Discard<br>Reasons:<br>Not of interest
526.	New approaches to the treatment of inflammatory disease: Focus on small-molecule inhibitors of signal transduction pathways<br>Drugs in R and D 2008<br>9(6):397-434 doi:10.2165/0126839-200809060-00005	Scopus	Discard<br>Reasons:<br>Not of interest
527.	Newest evidence for tetrahydrocannabinol: cannabidiol oromucosal spray from randomized clinical trials<br>Markova J<br>NEURODEGENERATIVE DISEASE MANAGEMENT 2019<br>9:2 Supplement: S DOI: 10.2217/nmt-2018-0050	Pubmed<br>Scopus<br>Web of Science	Discard<br>Reasons:<br>Not of interest
528.	Newest evidence for tetrahydrocannabinol:cannabidiol oromucosal spray from postapproval pragmatic studies<br>Patti F.<br>Neurodegener Dis Manag. 2019<br>9(2s):3-7. doi: 10.2217/nmt-2018-0049.	Pubmed<br>Web of Science	Discard<br>Reasons:<br>Not of interest
529.	News about therapeutic use of Cannabis and endocannabinoid system [Novedades sobre las potencialidades terapéuticas del Cannabis y el sistema cannabinoide]<br>Duran M, Laporte JR, Capellà D<br>Medicina Clinica 2004<br>122(10):390-398 doi:10.1157/13059327	Scopus	Discard<br>Reasons:<br>Not of interest
530.	NFKF is a synthetic fragment derived from rat hemopressin that protects mice from neurodegeneration<br>Heimann AS, Giardini AC, Sant'Anna MB, Dos Santos NB, Gewehr MCF, Munhoz CD, Castro LM, Picolo G, Remer RA, Ferro ES<br>Neurosci Lett. 2020<br>721:134765 doi: 10.1016/j.neulet.2020.134765	Pubmed<br>Scopus<br>Web of Science	Discard<br>Reasons:<br>Not of interest
531.	Nonpsychotropic cannabinoid receptors regulate microglial cell migration.<br>Walter L, Franklin A, Witting A, Wade C, Xie YH, Kunos G, Mackie K, Stella N<br>JOURNAL OF NEUROSCIENCE 2003<br>23(4): 1398-1405 doi: 10.1523/JNEUROSCI.23-04-01398.2003	Web of Science	Discard<br>Reasons:<br>Not of interest.
532.	Nose-to-brain Delivery of Natural Compounds for the Treatment of Central Nervous System Disorders<br>Bicker J, Fortuna A, Alves G, Falcão,A	Scopus	Discard<br>Reasons:<br>Not of interest

	Curr Pharm Des 2020<br>26(5):594-619. doi: 10.2174/1381612826666200115101544		
533.	Nothing Ventured, Nothing Gained: Regulations Cripple Potentially Life-Saving Research of Illicit Substances<br>Kodye L. Abbott, Kristina S. Gill, Patrick C. Flannery, Dawn M. Boothe, Muralikrishnan Dhanasekaran, and Satyanarayana R. Pondugula<br>ACS Chem. Neurosci. 2020<br>11, 10, 1382–1384 doi: 10.1021/acschemneuro.0c00241	Scopus	Discard<br>Reasons:<br>Not of interest
534.	Novel approaches and current challenges with targeting the endocannabinoid system<br>Morales P, Jagerovic N.<br>Expert Opin Drug Discov. 2020<br>15(8): 917-930 doi:10.1080/17460441.2020.1752178	Pubmed<br>Scopus<br>Web of Science	Discard<br>Reasons:<br>Not of interest
535.	Novel role for cannabidiol in circadian clock function<br>Bifulco M, Navarra G, Laezza C, Pagano C<br>Addict Biol 2020<br>25(2):e12772. doi: 10.1111/adb.12772	Scopus	Discard<br>Reasons:<br>Not of interest
536.	Novel targets in multiple sclerosis: To oxidative stress and beyond<br>Chiurchiù V<br>Current Topics in Medicinal Chemistry 2014<br>14(22):2590-2599 doi:10.2174/1568026614666141203143801	Scopus	Discard<br>Reasons:<br>Not of interest
537.	Off-label use of cannabinoids efficacy and safety<br>Julia SG, Marta VR, Lourdes GR, Isabel GM, Amparo FG, Dolores BH<br>European Journal of Clinical Pharmacy 2017<br>19(3):158-163	Scopus	Discard<br>Reasons:<br>Not of interest
538.	On healthcare by popular appeal: critical assessment of benefit and risk in cannabidiol based dietary supplements.<br>Cogan PS<br>EXPERT REVIEW OF CLINICAL PHARMACOLOGY 2019<br>12(6): 501-511 DOI: 10.1080/17512433.2019.1612743	Web of Science	Discard<br>Reasons:<br>Not of interest
539.	On the pharmacological properties of Δ 9-tetrahydrocannabinol (THC)<br>Costa B<br>Chemistry and Biodiversity 2007<br>4(8):1664-1677 doi:10.1002/cbdv.200790146	Scopus	Discard<br>Reasons:<br>Not of interest
540.	Opioids and opioid receptors in multiple sclerosis<br>Mirshafiey A, Mohsenzadegan M<br>Journal of Chinese Clinical Medicine 2010<br>5(3):171-177	Scopus	Discard<br>Reasons:<br>Not of interest

541.	Opportunities in systems biology to discover mechanisms and repurpose drugs for CNS diseases<br>Mei H, Xia T, Feng G, Zhu J, Lin SM, Qiu Y<br>Drug Discovery Today 2012<br>17(21-22):1208-1216 doi:10.1016/j.drudis.2012.06.015	Scopus	Discard<br>Reasons:<br>Not of interest
542.	Oral administration of cannabis with lipids leads to high levels of cannabinoids in the intestinal lymphatic system and prominent immunomodulation<br>Zgair A, Lee JB, Wong JCM, Taha DA, Aram J, Di Virgilio D, McArthur JW, Cheng YK, Hennig IM, Barrett DA, Fischer PM, Constantinescu CS, Gershkovich P.<br>Sci Rep. 2017<br>7(1):14542. doi: 10.1038/s41598-017-15026-z	Pubmed<br>Scopus<br>Web of Science	Keep
543.	Oral cannabinoids for spasticity in multiple sclerosis: will attitude continue to limit use?<br>Metz L, Page S.<br>Lancet. 2003<br>362(9395):1513. No abstract available.	Pubmed	Discard<br>Reasons:<br>Not of interest
544.	Oromucosal Delta (9)-tetrahydrocannabinol/cannabidiol for neuropathic pain associated with multiple sclerosis: An uncontrolled, open-label, 2-year extension trial.<br>Rog DJ, Nurmikko TJ, Young CA<br>CLINICAL THERAPEUTICS 2007<br>29(9): 2068-2079 doi: 10.1016/j.clinthera.2007.09.013	Pubmed<br>Scopus<br>Web of Science	Discard<br>Reasons:<br>Not of interest
545.	Overview of cannabidiol (CBD) and its analogues: Structures, biological activities, and neuroprotective mechanisms in epilepsy and Alzheimer's disease.<br>Li H, Liu Y, Tian D, Tian L, Ju X, Qi L, Wang Y, Liang C<br>European Journal of Medicinal Chemistry 2020<br>192:112163 doi: 10.1016/j.ejmech.2020.112163	Web of Science	Discard<br>Reasons:<br>Not of interest
546.	Overview of MS spasticity<br>Pozzilli C<br>European Neurology 2014<br>71SUPPL.1:1-3 doi:10.1159/000357739	Pubmed<br>Scopus	Discard<br>Reasons:<br>Not of interest
547.	Pain and multiple sclerosis: Pathophysiology and treatment topical collection on demyelinating disorders<br>Solaro C, Trabucco E, Messmer Uccelli M<br>Current Neurology and Neuroscience Reports 2013<br>13(1):320 doi:10.1007/s11910-012-0320	Scopus	Discard<br>Reasons:<br>Not of interest
548.	Pain associated with multiple sclerosis: Systematic review and proposed classification<br>O'Connor AB, Schwid SR, Herrmann DN, Markman JD, Dworkin RH<br>Pain 2008	Scopus	Discard<br>Reasons:<br>Not of interest

	137(1):96-111 doi:10.1016/j.pain.2007.08.024		
549.	Pain in multiple sclerosis: Prevalence, mechanisms, types and treatment [El dolor en la esclerosis múltiple: Prevalencia, mecanismos, tipos y tratamiento]<br>Bermejo PE, Oreja-Guevara C, Díez-Tejedor E<br>Revista de Neurologia 2010<br>50(2):101-108	Scopus	Discard<br>Reasons:<br>Not of interest
550.	Pain Modulation after Oromucosal Cannabinoid Spray (SATIVEX(Â®)) in Patients with Multiple Sclerosis: A Study with Quantitative Sensory Testing and Laser-Evoked Potentials<br>Turri M, Teatini F, Donato F, Zanette G, Tugnoli V, Deotto L, Bonetti B, Squintani G.<br>Medicines (Basel). 2018<br>5(3). pii: E59. doi: 10.3390/medicines5030059	Pubmed	Discard<br>Reasons:<br>Not of interest
551.	Pain relief with cannabinoids-- the importance of endocannabinoids and cannabinoids for pain therapy<br>Karst M, Bernateck M.<br>Anasthesiol Intensivmed Notfallmed Schmerzther. 2008<br>43(7-8):522-8. doi: 10.1055/s-0028-1083094	Pubmed<br>Scopus	Discard<br>Reasons:<br>Not of interest
552.	Pain Therapeutics - SMi Conference 11-12 June 2003, London, UK<br>De La Rue SA<br>IDrugs 2003<br>6(7):652-655	Scopus	Discard<br>Reasons:<br>Not of interest
553.	Pain therapy in multiple sclerosis [Schmerzbehandlung bei multipler sklerose]<br>Van Schayck RH<br>Neurologie und Rehabilitation 2006<br>12(4):180-194	Scopus	Discard<br>Reasons:<br>Not of interest
554.	Palatability and oral cavity tolerability of THC: CBD oromucosal spray and possible improvement measures in multiple sclerosis patients with resistant spasticity: a pilot study.<br>Lus G, Cantello R, Danni MC, Rini A, Sarchielli P, Tassinari T, Signoriello E<br>Neurodegenerative Disease Management 2018<br>8(2): 105-113 doi: 10.2217/nmt-2017-0056	Pubmed<br>Scopus<br>Web of Science	Discard<br>Reasons:<br>Not of interest
555.	Pathophysiology, assessment and management of multiple sclerosis spasticity: an update<br>Haas J.<br>Expert Rev Neurother. 2011<br>11(4 Suppl):3-8. Review	Pubmed<br>Scopus	Discard<br>Reasons:<br>Not of interest
556.	Pathways and gene networks mediating the regulatory effects of cannabidiol, a nonpsychoactive cannabinoid, in autoimmune T cells<br>Kozela E, Juknat A, Gao F, Kaushansky N, Coppola G, Vogel Z.	Pubmed<br>Scopus<br>Web of Science	Keep

	J Neuroinflammation. 2016<br>13(1):136. doi: 10.1186/s12974-016-0603-x		
557.	Patient Counseling Guidelines for the Use of Cannabis for the Treatment of Chemotherapy-Induced Nausea/Vomiting and Chronic Pain<br>Makary P, Parmar JR, Mims N, Khanfar NM, Freeman RA<br>Journal of Pain and Palliative Care Pharmacotherapy 2018<br>32(4):216-225 doi:10.1080/15360288.2019.1598531	Scopus	Discard<br>Reasons:<br>Not of interest
558.	Patterns of medicinal cannabis use, strain analysis, and substitution effect among patients with migraine, headache, arthritis, and chronic pain in a medicinal cannabis cohort<br>Baron EP, Lucas P, Eades J, Hogue O<br>Journal of Headache and Pain 2018,<br>19(1):37 doi:10.1186/s10194-018-0862-2	Scopus<br>Web of Science	Discard<br>Reasons:<br>Not of interest
559.	Perspectives on Cannabis-Based Therapy of Multiple Sclerosis: A Mini-Review<br>Mecha M, Carrillo-Salinas FJ, Feliú A, Mestre L, Guaza C<br>Front. Cell. Neurosci. 2020<br>doi:10.3389/fncel.2020.00034	Scopus	Discard<br>Reasons:<br>Not of interest
560.	Pharmacist’s Guide to CBD Oil<br>Treese NM<br>US Pharm. 2020<br>45(3):20-23	Web of Science	Discard<br>Reasons:<br>Not of interest
561.	Pharmacokinetic evaluation of nabiximols for the treatment of multiple sclerosis pain<br>Tanasescu R, Constantinescu CS.<br>Expert Opin Drug Metab Toxicol. 2013<br>9(9):1219-28. doi: 10.1517/17425255.2013.795542.	Pubmed<br>Scopus<br>Web of Science	Discard<br>Reasons:<br>Not of interest
562.	Pharmacokinetics and pharmacodynamics of cannabinoids.<br>Grotenhermen F<br>Clinical Pharmacokinetics 2003<br>42(4): 327-360 DOI: 10.2165/00003088-200342040-00003	Web of Science	Discard<br>Reasons:<br>Not of interest
563.	Pharmacokinetics of Sativex® in Dogs: Towards a Potential Cannabinoid-Based Therapy for Canine Disorders<br>Fernàndez-Trapero M, Pèrez-Dìaz C, Espejo-Porras F, de Lago E, Fernàndez-Ruiz J. Biomolecules. 2020<br>10(2):279. doi: 10.3390/biom10020279	Pubmed<br>Scopus<br>Web of Science	Discard<br>Reasons:<br>Not of interest
564.	Pharmacologic Treatment Tools: Systemic Medications and Toxins, Opportunities, and Pitfalls<br>Riedel P, Marino MH<br>Physical Medicine and Rehabilitation Clinics of North America 2018	Scopus	Discard<br>Reasons:<br>Not of interest

	29(3):501-517 doi:10.1016/j.pmr.2018.04.008		
565.	Pharmacological and therapeutic targets for Delta(9)-tetrahydrocannabinol and cannabidiol.<br>Pertwee RG<br>EUPHYTICA 2004<br>140(1-2): 73-82 doi: 10.1007/s10681-004-4756-9	Web of Science	Discard<br>Reasons:<br>Not of interest
566.	Pharmacological management of pain in patients with multiple sclerosis<br>Solaro C, Messmer Uccelli M<br>Drugs 2010<br>70(10):1245-1254 doi:10.2165/11537930-000000000-00000	Scopus	Discard<br>Reasons:<br>Not of interest
567.	Pharmacological management of spasticity in multiple sclerosis: Systematic review and consensus paper<br>Otero-Romero S, Sastre-Garriga J, Comi G, Hartung HP, Soelberg SÃ¸rensen P, Thompson AJ, Vermersch P, Gold R, Montalban X.<br>Mult Scler. 2016<br>22(11):1386-1396 doi: 10.1177/1352458516643600	Pubmed<br>Scopus<br>Web of Science	Discard<br>Reasons:<br>Not of interest
568.	Pharmacological properties and therapeutic possibilities for drugs acting upon endocannabinoid receptors<br>Fowler CJ<br>Current Drug Targets: CNS and Neurological Disorders 2005<br>4(6):685-696 doi:10.2174/156800705774933041	Scopus	Discard<br>Reasons:<br>Not of interest
569.	Pharmacology and potential therapeutic uses of cannabis<br>Hirst RA, Lambert DG, Nottcutt WG<br>British Journal of Anaesthesia 1998<br>81(1):77-84 doi:10.1093/bja/81.1.77	Scopus	Discard<br>Reasons:<br>Not of interest
570.	Pharmacology and toxicology of cannabis derivatives and endocannabinoid agonists<br>Gerra G, Zaimovic A, Gerra ML, Ciccocioppo R, Cippitelli A, Serpelloni G, Somaini L<br>Recent Patents on CNS Drug Discovery 2010<br>5(1):46-52 doi:10.2174/157488910789753521	Scopus	Discard<br>Reasons:<br>Not of interest
571.	Pharmacology of Medical Cannabis<br>Md Ruhul AminDeclan W. Ali<br>Recent Advances in Cannabinoid Physiology and Pathology 2019<br>151-165	Web of Science	Discard<br>Reasons:<br>Not of interest
572.	Pharmacotherapeuetic options for the treatment of Multiple sclerosis<br>Palmer AM<br>Clinical Medicine Insights: Therapeutics 2012<br>4(145):168 doi:10.4137/CMT.S8661	Scopus	Discard<br>Reasons:<br>Not of interest

573.	Pharmacotherapeutic considerations for use of cannabinoids to relieve pain in patients with malignant diseases.<br>Darkovska-Serafimovska M, Serafimovska T, Arsova-Sarafinovska Z, Stefanoski S, Keskovski Z, Balkanov T<br>Journal of Pain Research 2018<br>11:837-842 doi: 10.2147/JPR.S160556	Web of Science	Discard<br>Reasons:<br>Not of interest
574.	Phytocannabinoids and cannabimimetic drugs: Recent patents in central nervous system disorders<br>Ranieri R, Marasco D, Bifulco M, Malfitano AM<br>Recent Patents on CNS Drug Discovery 2016<br>10(2):157-177 doi:10.2174/1574889810666160517123938	Scopus	Discard<br>Reasons:<br>Not of interest
575.	Phytocannabinoids as novel therapeutic agents in CNS disorders.<br>Hill AJ, Williams CM, Whalley BJ, Stephens GJ<br>PHARMACOLOGY & THERAPEUTICS 2012<br>133(1): 79-97 doi: 10.1016/j.pharmthera.2011.09.002	Web of Science	Discard<br>Reasons:<br>Not of interest
576.	Phytochemistry of Cannabis sativa L<br>ElSohly MA, Radwan, MM, Gul W, Chandra S, Galal A<br>Progress in the chemistry of organic natural products 2017<br>103(1):36-55 doi:10.1007/978-3-319-45541-9_1	Pubmed<br>Scopus	Discard<br>Reasons:<br>Not of interest
577.	Phytotherapy as a complementary medicine for multiple sclerosis [Multipl sklerozda tamamlayıcı tedavi olarak fitoterapi]<br>Rabiei Z<br>Turkish Journal of Pharmaceutical Sciences 2019<br>16(2):246-251 doi:10.4274/tjps.galenos.2018.90522	Scopus	Discard<br>Reasons:<br>Not of interest
578.	Place of cannabinoids in medicine [Stellenwert der Cannabinoide in der Medizin]<br>Müller-Vahl K, Grotenhermen F<br>Padiatrische Praxis 2017<br>88(1):147-155	Scopus	Discard<br>Reasons:<br>Not of interest
579.	Placebo effects in a multiple sclerosis spasticity enriched clinical trial with the oromucosal cannabinoid spray (THC/CBD): dimension and possible causes<br>Di Marzo V, Centonze D.<br>CNS Neurosci Ther. 2015<br>21(3):215-21. doi: 10.1111/cns.12358.	Pubmed<br>Scopus<br>Web of Science	Discard<br>Reasons:<br>Not of interest
580.	Plant, synthetic, and endogenous cannabinoids in medicine<br>Di Marzo V, De Petrocellis L<br>Annual Review of Medicine 2006<br>57:553-574 doi:10.1146/annurev.med.57.011205.135648	Scopus	Discard<br>Reasons:<br>Not of interest

581.	Plasma Cannabinoid Pharmacokinetics following Controlled Oral Delta(9)-Tetrahydrocannabinol and Oromucosal Cannabis Extract Administration.<br>Karschner EL, Darwin WD, Goodwin RS, Wright S, Huestis MA<br>CLINICAL CHEMISTRY 2011<br>57(1): 66-75 doi: 10.1373/clinchem.2010.152439	Web of Science	Discard<br>Reasons:<br>Not of interest
582.	Plastic and neuroprotective mechanisms involved in the therapeutic effects of cannabidiol in psychiatric disorders<br>Campos AC, Fogaça MV, Scarante FF, Joca SRL, Sales AJ, Gomes FV, Sonego AB, Rodrigues NS, Galve-Roperh I, Guimarães FS<br>Frontiers in Pharmacology 2017<br>269 doi:10.3389/fphar.2017.00269	Scopus	Discard<br>Reasons:<br>Not of interest
583.	Poly-epsilon-caprolactone microspheres as a drug delivery system for cannabinoid administration: Development, characterization and in vitro evaluation of their antitumoral efficacy.<br>de la Ossa DHP, Ligresti A, Gil-Alegre ME, Aberturas MR, Molpeceres J, Di Marzo V, Suarez AIT<br>JOURNAL OF CONTROLLED RELEASE 2012<br>161(3): 927-932 doi: 10.1016/j.jconrel.2012.05.003	Web of Science	Discard<br>Reasons:<br>Not of interest
584.	Polypharmacology Shakes Hands with Complex Aetiopathology.<br>Brodie JS, Di Marzo V, Guy GW<br>TRENDS IN PHARMACOLOGICAL SCIENCES 2015<br>36(12): 802-821 doi: 10.1016/j.tips.2015.08.010	Web of Science	Discard<br>Reasons:<br>Not of interest
585.	Pot and pain<br>Miller G<br>Science 2016<br>354(6312):566-568 doi:10.1126/science.354.6312.566	Scopus	Discard<br>Reasons:<br>Not of interest
586.	Potential control of multiple sclerosis by cannabis and the endocannabinoid system<br>Pryce G, Baker D<br>CNS and Neurological Disorders - Drug Targets 2012<br>11(5):624-641 doi:10.2174/187152712801661310	Scopus	Discard<br>Reasons:<br>Not of interest
587.	Preclinical and clinical assessment of cannabinoids as anti-cancer agents<br>Ladin DA, Soliman E, Griffin L, Van Dross R<br>Frontiers in Pharmacology 2016<br>7:361 doi:10.3389/fphar.2016.00361	Scopus	Discard<br>Reasons:<br>Not of interest
588.	Predictors of Nabiximols (Sativex(®)) discontinuation over long-term follow-up: a real-life study	Pubmed<br>Scopus	Discard<br>Reasons:<br>Not of interest

	Carotenuto A, Costabile T, De Lucia M, Moccia M, Falco F, Petruzzo M, De Angelis M, Russo CV, Saccà F, Lanzillo R, Brescia Morra V.<br>J Neurol. 2020<br>267(6):1737-1743. doi: 10.1007/s00415-020-09739-x.		
589.	Preparation and characterization of Delta (9)-tetrahydrocannabinol-loaded biodegradable polymeric microparticles and their antitumoral efficacy on cancer cell lines.<br>de la Ossa DHP, Gil-Alegre ME, Ligresti A, Aberturas MD, Molpeceres J, Torres AI, Di Marzo V<br>JOURNAL OF DRUG TARGETING 2013<br>21(8): 710-718 doi: 10.3109/1061186X.2013.809089	Web of Science	Discard<br>Reasons:<br>Not of interest
590.	Prescribing cannabinoids for multiple sclerosis - Current issues.<br>Pertwee RG<br>CNS DRUGS 1999<br>11(5): 327-334 doi: 10.2165/00023210-199911050-00001	Web of Science	Discard<br>Reasons:<br>Not of interest
591.	Presymptomatic diagnosis with MRI and adequate treatment ameliorate the outcome after natalizumab-associated progressive multifocal leukoencephalopathy<br>Lindå H, von Heijne A<br>Frontiers in Neurology 2013<br>11:31 doi:10.3389/fneur.2013.00011	Scopus	Discard<br>Reasons:<br>Not of interest
592.	Product update<br>Rempel P<br>Canadian Pharmacists Journal 2010<br>143(6):268-270 doi:10.3821/1913-701X-143.6.268	Scopus	Discard<br>Reasons:<br>Not of interest
593.	Progress in cannabis research from a pharmaceutical chemist's point of view.<br>Muller CE<br>BUNDESGESUNDHEITSBLATT-GESUNDHEITSFORSCHUNG-GESUNDHEITSSCHUTZ 2019<br>62(7): 818-824 doi: 10.1007/s00103-019-02964-4	Web of Science	Discard<br>Reasons:<br>Not of interest
594.	Progress in study of new drugs for treatment of multiple sclerosis<br>Chen Z-Z<br>Pharmaceutical Biotechnology 2009<br>16(2):186-188	Scopus	Discard<br>Reasons:<br>Not of interest
595.	Pros and cons of medical cannabis use by people with chronic brain disorders<br>Suryadevara U, Bruijnzeel DM, Nuthi M, Jagnarine DA, Tandon R, Bruijnzeel AW<br>Current Neuropharmacology 2017<br>15(6):800-814 doi:10.2174/1570159X14666161101095325	Scopus	Discard<br>Reasons:<br>Not of interest

596.	Protective Effects of Cannabidiol against Seizures and Neuronal Death in a Rat Model of Mesial Temporal Lobe Epilepsy.<br>Do Val-da Silva RA, Peixoto-Santos JE, Kandratavicius L, De Ross JB, Esteves I, De Martinis BS, Alves MNR, Scandiuzzi RC, Hallak JEC, Zuardi, AW, Crippa JA, Leite JP<br>Frontiers in Pharmacology 2017<br>8 Article Number: 131 doi: 10.3389/fphar.2017.00131	Web of Science	Discard<br>Reasons:<br>Not of interest
597.	Protective effects of cannabidiol on cuprizone-induced demyelination in C57BL/6 mice.<br>Sajjadian M, Kashani IR, Pasbakhsh P, Hassani M, Omidi A, Takzare N, Clarner T, Beyer C, Zendedel A<br>Journal of Contemporary Medical Sciences 2017<br>3(11): 278-283 doi: 10.22317/jcms.09201707	Web of Science	Keep
598.	Protective Effects of Cannabidiol on Lesion-Induced Intervertebral Disc Degeneration.<br>Silveira JW, Issy AC, Castania VA, Salmon CEG, Nogueira-Barbosa MH Guimaraes, FS, Defino HLA, Del Bel E<br>PLOS ONE 2014<br>9(12) Article Number: e113161 doi: 10.1371/journal.pone.0113161	Web of Science	Discard<br>Reasons:<br>Not of interest
599.	Psychopathological and cognitive effects of therapeutic cannabinoids in multiple sclerosis: a double-blind, placebo controlled, crossover study<br>Aragona M, Onesti E, Tomassini V, Conte A, Gupta S, Gilio F, Pantano P, Pozzilli C, Inghilleri M.<br>Clin Neuropharmacol. 2009<br>32(1):41-7. doi: 10.1097/WNF.0B013E3181633497	Pubmed<br>Scopus	Discard<br>Reasons:<br>Not of interest
600.	PTL401, a New Formulation Based on Pro-Nano Dispersion Technology, Improves Oral Cannabinoids Bioavailability in Healthy Volunteers. Atsmon J, Cherniakov I, Izgelov D, Hoffman A, Domb AJ, Deutsch L, Deutsch F, Heffetz D, Sacks H<br>Journal of Pharmaceutical Sciences 2018<br>107(5): 1423-1429 doi: 10.1016/j.xphs.2017.12.020	Web of Science	Discard<br>Reasons:<br>Not of interest
601.	Purified Cannabidiol, the main non-psychotropic component of Cannabis sativa, alone, counteracts neuronal apoptosis in experimental multiple sclerosis<br>Giacoppo S, Soundara Rajan T, Galuppo M, Pollastro F, Grassi G, Bramanti P, Mazzon E.<br>Eur Rev Med Pharmacol Sci. 2015<br>19(24):4906-19	Pubmed<br>Scopus<br>Web of Science	Discard<br>Reasons:<br>Not of interest
602.	Randomized controlled trial of cannabis-based medicine in spasticity caused by multiple sclerosis<br>Collin C, Davies P, Mutiboko IK, Ratcliffe S<br>Eur J Neurol. 2007	Pubmed<br>Scopus<br>Web of Science	Discard<br>Reasons:<br>Not of interest

	14(3):290-6 doi: 10.1111/j.1468-1331.2006.01639.x		
603.	Randomized controlled trial of Sativex to treat detrusor overactivity in multiple sclerosis<br>Kavia RB, De Ridder D, Constantinescu CS, Stott CG, Fowler CJ.<br>Mult Scler. 2010<br>16(11):1349-59. doi: 10.1177/1352458510378020.	Pubmed<br>Scopus<br>Web of Science	Discard<br>Reasons:<br>Not of interest
604.	Randomized, controlled trial of cannabis-based medicine in central pain in multiple sclerosis<br>Rog DJ, Nurmikko TJ, Friede T, Young CA.<br>Neurology. 2005<br>65(6):812-9 doi: 10.1212/01.wnl.0000176753.45410.8b	Pubmed<br>Scopus<br>Web of Science	Discard<br>Reasons:<br>Not of interest
605.	Real world experience of patients with amyotrophic lateral sclerosis (ALS) in the treatment of spasticity using tetrahydrocannabinol:cannabidiol (THC:CBD)<br>Meyer T, Funke A, MÃ¼nch C, Kettemann D, Maier A, Walter B, Thomas A, Spittel S.<br>BMC Neurol. 2019<br>19(1):222. doi: 10.1186/s12883-019-1443-y	Pubmed<br>Scopus<br>Web of Science	Discard<br>Reasons:<br>Not of interest
606.	Reassessment of the role of cannabinoids in the management of pain<br>Beaulieu P, Ware M<br>Current Opinion in Anaesthesiology 2007<br>20(5):473-477 doi:10.1097/ACO.0b013e3282efd175	Scopus	Discard<br>Reasons:<br>Not of interest
607.	Refractory trigeminal neuralgia responsive to nabiximols in a patient with multiple sclerosis<br>Gajofatto A.<br>Mult Scler Relat Disord. 2016<br>8:64-5. doi: 10.1016/j.msard.2016.05.004.	Pubmed<br>Scopus<br>Web of Science	Discard<br>Reasons:<br>Not of interest
608.	Report of objective clinical responses of cancer patients to pharmaceutical-grade synthetic cannabidiol<br>Kenyon J, Liu W, Dalgleish A<br>Anticancer Research 2018<br>38(10):5831-5835 doi:10.21873/anticanres.12924	Scopus	Discard<br>Reasons:<br>Not of interest
609.	Research into cannabinoid medicines may suggest potential therapeutic opportunities<br>Knott C<br>Pharmacy in Practice 2008<br>18(3):96-109	Scopus	Discard<br>Reasons:<br>Not of interest
610.	Restatement. Therapeutic use of cannabis derivatives [Mise au point. Usage therapeutique des dérivés du cannabis]<br>Benyamina A, Reynaud M<br>Revue du Praticien 2014	Scopus	Discard<br>Reasons:<br>Not of interest

	64(2):165-168		
611.	Resveratrol: More than a phytochemical<br>Hassanzadeh P, Atyabi F, Dinarvand R<br>Biomedical Reviews 2015<br>26(13):21 doi:10.14748/bmr.v26.1540	Scopus	Discard<br>Reasons:<br>Not of interest
612.	Review of the neurological benefits of phytocannabinoids<br>Maroon J, Bost J.<br>Surg Neurol Int. 2018<br>9:91. doi: 10.4103/sni.sni_45_18.	Pubmed	Discard<br>Reasons:<br>Not of interest
613.	Review:Weakevidence of benefits of cannabis for chronic neuropathic pain; Moderate to weak evidence of adverse effects<br>Murff HJ<br>Annals of Internal Medicine 2017<br>167(12): JC62 doi:10.7326/ACPJC-2017-167-12-062	Scopus	Discard<br>Reasons:<br>Not of interest
614.	Role of adenosine in diabetic retinopathy<br>Liou GI, Ahmad S, Naime M, Fatteh N, Ibrahim AS<br>Journal of Ocular Biology, Diseases, and Informatics 2011<br>4(1-2):19-24 doi:10.1007/s12177-011-9067-5	Scopus	Discard<br>Reasons:<br>Not of interest
615.	Role of cannabinoids in multiple sclerosis<br>Zajicek JP, Apostu VI<br>CNS Drugs 2011<br>25(3):187-201 doi:10.2165/11539000-000000000-00000	Scopus	Discard<br>Reasons:<br>Not of interest
616.	Role of cannabinoids in the management of neuropathic pain<br>Martín Fontelles MI, Goicoechea García C<br>CNS Drugs 2008<br>22(8):645-653 doi:10.2165/00023210-200822080-00003	Scopus	Discard<br>Reasons:<br>Not of interest
617.	Role of Myeloid-Derived Suppressor Cells in Amelioration of Experimental Autoimmune Hepatitis Following Activation of TRPV1 Receptors by Cannabidiol.<br>Hegde VL, Nagarkatti PS, Nagarkatti M<br>PLOS ONE 2011<br>6(4) Article Number: e18281 doi: 10.1371/journal.pone.0018281	Web of Science	Discard<br>Reasons:<br>Not of interest
618.	Role of the cannabinoid system in pain control and therapeutic implications for the management of acute and chronic pain episodes<br>Manzanares J, Julian M, Carrascosa A.<br>Curr Neuropharmacol. 2006<br>4(3):239-57. doi: 10.2174/157015906778019527	Pubmed<br>Scopus<br>Web of Science	Discard<br>Reasons:<br>Not of interest

619.	Safety and efficacy of nabiximols on spasticity symptoms in patients with motor neuron disease (CANALS): a multicentre, double-blind, randomised, placebo-controlled, phase 2 trial<br>Riva N, Mora G, SorarÃ¹ G, Lunetta C, Ferraro OE, Falzone Y, Leocani L, Fazio R, Comola M, Comi G<br>Lancet Neurol. 2019<br>18(2):155-164. doi: 10.1016/S1474-4422(18)30406-X.	Pubmed<br>Scopus<br>Web of Science	Discard<br>Reasons:<br>Not of interest
620.	Safety and Toxicology of Cannabinoids.<br>Sachs J, McGlade E, Yurgelun Todd D<br>NEUROTHERAPEUTICS 2015<br>12(4): 735-746 doi: 10.1007/s13311-015-0380-8	Web of Science	Discard<br>Reasons:<br>Not of interest
621.	Safety, efficacy, and mechanisms of action of cannabinoids in neurological disorders<br>Friedman D, French JA, Maccarrone M.<br>Lancet Neurol. 2019<br>18(5):504-512. doi: 10.1016/S1474-4422(19)30032-8	Pubmed<br>Scopus<br>Web of Science	Discard<br>Reasons:<br>Not of interest
622.	Safety, tolerability, and efficacy of orally administered cannabinoids in MS<br>Russo EB.<br>Neurology. 2003<br>60(4):729-30 author reply 729-30. No abstract available.	Pubmed	Discard<br>Reasons:<br>Not of interest
623.	Sativex (R) in multiple sclerosis spasticity: a cost-effectiveness model.<br>Slof J, Gras A<br>Expert Review of Pharmacoeconomics & Outcomes Research 2012<br>12(4): 525-538 doi: 10.1586/ERP.12.40	Pubmed<br>Scopus<br>Web of Science	Discard<br>Reasons:<br>Not of interest
624.	Sativex (R): clinical efficacy and tolerability in the treatment of symptoms of multiple sclerosis and neuropathic pain.<br>Barnes MP<br>Expert Opinion on Pharmacotherapy 2006<br>7(5): 607-615 doi: 10.1517/14656566.7.5.607	Scopus<br>Web of Science	Discard<br>Reasons:<br>Not of interest
625.	Sativex ® as add-on therapy vs. further optimized first-line ANTispastics (SAVANT) in resistant multiple sclerosis spasticity: a double-blind, placebo-controlled randomised clinical trial<br>MarkovÃ J, Essner U, Akmaz B, Marinelli M, Trompke C, Lentschat A, Vila C<br>International Journal of Neuroscience 2019<br>129(2):119-128 doi:10.1080/00207454.2018.1481066	Pubmed<br>Scopus<br>Web of Science	Discard<br>Reasons:<br>Not of interest
626.	Sativex for the management of multiple sclerosis symptoms<br>Perras C.<br>Issues Emerg Health Technol. 2005	Pubmed<br>Scopus	Discard<br>Reasons:<br>Not of interest

	(72):1-4		
627.	Sativex in resistant multiple sclerosis spasticity: Discontinuation study in a large population of Italian patients (SA.FE. study)<br>Messina S, Solaro C, Righini I, Bergamaschi R, Bonavita S, Bossio RB, Brescia Morra V, Costantino G, Cavalla P, Centonze D, Comi G, Cottone S, Danni MC, Francia A, Gajofatto A, Gasperini C, Zaffaroni M, Petrucci L, Signoriello E, Maniscalco GT, Spinicci G, Matta M, et al.<br>PLoS One. 2017<br>12(8):e0180651. doi: 10.1371/journal.pone.0180651.	Pubmed<br>Scopus	Discard<br>Reasons:<br>Not of interest
628.	Sativex in the management of multiple sclerosis-related spasticity: An overview of the last decade of clinical evaluation<br>Giacoppo S, Bramanti P, Mazzon E.<br>Mult Scler Relat Disord. 2017<br>17:22-31. doi: 10.1016/j.msard.2017.06.015.	Pubmed<br>Scopus<br>Web of Science	Discard<br>Reasons:<br>Not of interest
629.	Sativex in the management of multiple sclerosis-related spasticity: role of the corticospinal modulation<br>Russo M, CalabrÃ² RS, Naro A, Sessa E, Rifici C, D'Aleo G, Leo A, De Luca R, Quartarone A, Bramanti P.<br>Neural Plast. 2015<br>2015:656582. doi: 10.1155/2015/656582.	Pubmed<br>Scopus	Discard<br>Reasons:<br>Not of interest
630.	Sativex long-term use: an open-label trial in patients with spasticity due to multiple sclerosis<br>Serpell MG, Notcutt W, Collin C.<br>J Neurol. 2013<br>260(1):285-95. doi: 10.1007/s00415-012-6634-z.	Pubmed<br>Scopus<br>Web of Science	Discard<br>Reasons:<br>Not of interest
631.	Sativex(®) and clinical-neurophysiological measures of spasticity in progressive multiple sclerosis.<br>Leocani L, Nuara A, Houdayer E, Schiavetti I, Del Carro U, Amadio S, Straffi L, Rossi P, Martinelli V, Vila C, Sormani MP, Comi G<br>Journal of Neurology 2015<br>262(11): 2520-2527 doi: 10.1007/s00415-015-7878-1	Pubmed<br>Scopus<br>Web of Science	Discard<br>Reasons:<br>Not of interest
632.	Sativex(®) (tetrahydrocannabinol + cannabidiol), an endocannabinoid system modulator: basic features and main clinical data<br>Vermersch P.<br>Expert Rev Neurother. 2011<br>11(4 Suppl):15-9. Review doi: 10.1586/ern.11.27	Pubmed<br>Scopus	Discard<br>Reasons:<br>Not of interest

633.	Sativex: clinical efficacy and tolerability in the treatment of symptoms of multiple sclerosis and neuropathic pain<br>Barnes MP.<br>Expert Opin Pharmacother. 2006<br>7(5):607-15. Review doi: 10.1517/14656566.7.5.607	Pubmed<br>Scopus<br>Web of Science	Discard<br>Reasons:<br>Not of interest
634.	Sativex® effects on promoter methylation and on CNR1/CNR2 expression in peripheral blood mononuclear cells of progressive multiple sclerosis patients<br>Santoro M, Mirabella M, De Fino C, Bianco A, Lucchini M, Losavio F, Sabino A, Nociti V<br>Journal of the Neurological Sciences 2017<br>379(298):303 doi:10.1016/j.jns.2017.06.017	Pubmed<br>Scopus	Keep
635.	Sativex-induced neurobehavioral effects: causal or concausal? A practical advice!<br>Russo M, Rifici C, Sessa E, D'Aleo G, Bramanti P, CalabrÃ² RS.<br>Daru. 2015<br>23:25. doi: 10.1186/s40199-015-0109-6	Pubmed<br>Scopus<br>Web of Science	Discard<br>Reasons:<br>Not of interest
636.	Schmerzlinderung durch Cannabinoide? Bedeutung des Endocannabinoidsystems und der Cannabinoide für die Schmerztherapie<br>Karst M, Bernateck M<br>Anasthesiologie Intensivmedizin Notfallmedizin Schmerztherapie 2008<br>43:522-528 doi:10.1055/s-0028-1083094	Pubmed<br>Scopus	Discard<br>Reasons:<br>Not of interest
637.	Scientific Opinion on the risks for human health related to the presence of tetrahydrocannabinol (THC) in milk and other food of animal origin. Benford D, Ceccatelli S, Cottrill B, DiNovi M, Dogliotti E, Edler L, Farmer P, Furst P, Hoogenboom L, Knutsen HK, Lundebye AK, Metzler M, Mutti A, Nebbia CS, O'Keeffe M, Petersen A, Rietjens I, Schrenk D, Silano V, van Loveren H, Vleminckx C, Wester P<br>EFSA JOURNAL 2015<br>13(6) Article Number: UNSP 4141 doi: 10.2903/j.efsa.2015.4141	Web of Science	Discard<br>Reasons:<br>Not of interest
638.	Selective Cannabinoids for Chronic Neuropathic Pain: A Systematic Review and Meta-analysis.<br>Meng H, Johnston B, Englesakis M, Moulin DE, Bhatia A<br>ANESTHESIA AND ANALGESIA 2017<br>125(5): 1638-1652 doi: 10.1213/ANE.0000000000002110	Web of Science	Discard<br>Reasons:<br>Not of interest
639.	Short-Term Efficacy of CBD-Enriched Hemp Oil in Girls with Dysautonomic Syndrome after Human Papillomavirus Vaccination<br>Palmieri B, Laurino C, VadalÃ M.<br>Isr Med Assoc J. 2017<br>19(2):79-84	Pubmed<br>Scopus<br>Web of Science	Discard<br>Reasons:<br>Not of interest

640.	Should cannabis be prescribed on the NHS?<br>Layward L<br>Pharmaceutical Journal 1996<br>256(6885):457	Scopus	Discard<br>Reasons:<br>Not of interest
641.	Should doctors prescribe cannabinoids?<br>Farrell M, Buchbinder R, Hall W<br>BMJ (Online) 2014<br>48:g2737 doi:10.1136/bmj.g2737	Scopus<br>Web of Science	Discard<br>Reasons:<br>Not of interest
642.	Should patient groups be more transparent about their funding?<br>Arie S, Mahony C.<br>BMJ. 2014<br>349:g5892. doi: 10.1136/bmj.g5892. No abstract available	Pubmed<br>Scopus	Discard<br>Reasons:<br>Not of interest
643.	Should we care about sativex-induced neurobehavioral effects? A 6-month follow-up study<br>Russo M, De Luca R, Torrisi M, Rifici C, Sessa E, Bramanti P, Naro A, CalabrÃ² RS.<br>Eur Rev Med Pharmacol Sci. 2016<br>20(14):3127-33	Pubmed<br>Scopus	Discard<br>Reasons:<br>Not of interest
644.	Source of cannabinoids: what is available, what is used, and where does it come from?<br>Specchio N, Pietrafusa N, Cross HJ.<br>Epileptic Disord. 2020<br>22 (S1): S1-S9 doi: 10.1684/epd.2019.1121	Pubmed<br>Scopus<br>Web of Science	Discard<br>Reasons:<br>Not of interest
645.	Special Considerations and Assessment in Patients with Multiple Sclerosis<br>Maitin IB, Cruz E.<br>Phys Med Rehabil Clin N Am. 2018<br>29(3):473-481. doi: 10.1016/j.pmr.2018.03.003.	Pubmed<br>Scopus<br>Web of Science	Discard<br>Reasons:<br>Not of interest
646.	Splendor in the Grass? A Pilot Study Assessing the Impact of Medical Marijuana on Executive Function.<br>Gruber SA, Sagar KA, Dahlgren MK, Racine MT, Smith RT, Lukas SE<br>FRONTIERS IN PHARMACOLOGY 2016<br>7 Article Number: 355 doi: 10.3389/fphar.201E00355	Web of Science	Discard<br>Reasons:<br>Not of interest
647.	Summary of evidence-based guideline: complementary and alternative medicine in multiple sclerosis: report of the guideline development subcommittee of the American Academy of Neurology<br>Yadav V, Bever C Jr, Bowen J, Bowling A, Weinstock-Guttman B, Cameron M, Bourdette D, Gronseth GS, Narayanaswami P.<br>Neurology. 2014<br>82(12):1083-92. doi: 10.1212/WNL.0000000000000250	Pubmed<br>Scopus<br>Web of Science	Discard<br>Reasons:<br>Not of interest

648.	Summary of Evidence-based Guideline: Complementary and Alternative Medicine in Multiple Sclerosis: Report of the Guideline Development Subcommittee of the American Academy of Neurology<br>Wright S<br>Neurology 2014<br>83(16): 1485-1486 doi: 10.1212/01.wnl.0000455935.13606.91	Scopus<br>Web of Science	Discard<br>Reasons:<br>Not of interest
649.	Summary<br>Tomillero A, Moral MA<br>Methods and Findings in Experimental and Clinical Pharmacology 2010<br>32(2):133-147	Scopus	Discard<br>Reasons:<br>Not of interest
650.	Summary<br>Tomillero A, Moral MA<br>Methods and Findings in Experimental and Clinical Pharmacology 2010<br>32(5):331-388 doi:10.1358/mf.2010.32.5.1520420	Scopus	Discard<br>Reasons:<br>Not of interest
651.	Summary<br>Tomillero A, Moral MA<br>Methods and Findings in Experimental and Clinical Pharmacology 2010<br>32(4):247-288 doi:10.1358/mf.2010.32.4.1507200	Scopus	Discard<br>Reasons:<br>Not of interest
652.	Symptomatic therapy in multiple sclerosis: the role of cannabinoids in treating spasticity<br>Leussink VI, Husseini L, Warnke C, Broussalis E, Hartung HP, Kieseier BC.<br>Ther Adv Neurol Disord. 2012<br>5(5):255-66. doi: 10.1177/1756285612453972	Pubmed<br>Scopus<br>Web of Science	Discard<br>Reasons:<br>Not of interest
653.	Symptomatic treatment of multiple sclerosis using cannabinoids: recent advances<br>Smith PF.<br>Expert Rev Neurother. 2007<br>7(9):1157-63. Review doi: 10.1586/14737175.7.9.1157	Pubmed<br>Scopus	Discard<br>Reasons:<br>Not of interest
654.	Synthesis of (-)-Cannabimovone and Structural Reassignment of Anhydrocannabimovone through Gold(I)-Catalyzed Cycloisomerization.<br>Carreras J, Kirillova MS, Echavarren AM<br>ANGEWANDTE CHEMIE-INTERNATIONAL EDITION 2016<br>55(25): 7121-7125 doi: 10.1002/anie.201601834	Web of Science	Discard<br>Reasons:<br>Not of interest
655.	Systematic Review of the Costs and Benefits of Prescribed Cannabis-Based Medicines for the Management of Chronic Illness: Lessons from Multiple Sclerosis<br>Herzog S, Shanahan M, Grimison P, Tran A, Wong N, Lintzeris N, Simes J, Stockler M, Morton RL.<br>Pharmacoeconomics. 2018<br>36(1):67-78. doi: 10.1007/s40273-017-0565-6	Pubmed<br>Scopus<br>Web of Science	Discard<br>Reasons:<br>Not of interest

656.	Systematic Review of the Use of Tetrahydrocannabinol and Cannabidiol as a Medicinal Alternative in Multiple Sclerosis<br>Arellano D, Díaz O, Soto Molina H, Escobar Juárez Y<br>Value in Health 2020<br>23:10 doi: 10.1016/j.jval.2020.04.054	Web of Science	Discard<br>Reasons:<br>Not of interest
657.	Systematic review: efficacy and safety of medical marijuana in selected neurologic disorders: report of the Guideline Development Subcommittee of the American Academy of Neurology<br>Koppel BS, Brust JC, Fife T, Bronstein J, Youssof S, Gronseth G, Gloss D.<br>Neurology. 2014<br>82(17):1556-63. doi: 10.1212/WNL.0000000000000363.	Pubmed<br>Scopus<br>Web of Science	Discard<br>Reasons:<br>Not of interest
658.	Systemic Injections of Cannabidiol Enhance Acetylcholine Levels from Basal Forebrain in Rats.<br>Murillo-Rodriguez E, Arankowsky-Sandoval G, Rocha NB, Peniche-Amante R, Veras AB, Machado S, Budde H<br>NEUROCHEMICAL RESEARCH 2018<br>43(8): 1511-1518 DOI: 10.1007/s11064-018-2565-0	Web of Science	Discard<br>Reasons:<br>Not of interest
659.	Taming the cannabinoids: New potential in the pharmacologic control of lower urinary tract dysfunction<br>Apostolidis A<br>European Urology 2012<br>61(1):107-109 doi 10.1016/j.eururo.2011.09.025	Scopus	Discard<br>Reasons:<br>Not of interest
660.	Target regulation of PI3K/Akt/mTOR pathway by cannabidiol in treatment of experimental multiple sclerosis<br>Giacoppo S, Pollastro F, Grassi G, Bramanti P, Mazzon E.<br>Fitoterapia. 2017<br>116:77-84. doi: 10.1016/j.fitote.2016.11.010.	Pubmed<br>Scopus<br>Web of Science	Keep
661.	Targeting Cannabinoid Signaling in the Immune System: "High"-ly Exciting Questions, Possibilities, and Challenges.<br>Olah A, Szekanecz Z, Biro T<br>FRONTIERS IN IMMUNOLOGY 2017<br>8 Article Number: 1487 DOI: 10.3389/fimmu.2017.01487	Pubmed<br>Scopus<br>Web of Science	Discard<br>Reasons:<br>Not of interest
662.	Targeting the CB2 receptor for immune modulation<br>Lunn CA, Reich EP, Bober L<br>Expert Opinion on Therapeutic Targets 2006<br>10(5):653-663 doi:10.1517/14728222.10.5.653	Scopus	Discard<br>Reasons:<br>Not of interest
663.	Targeting the Endocannabinoid System in Psychiatric Illness	Scopus	Discard

	Katzman MA, Furtado M, Anand L<br>Journal of Clinical Psychopharmacology 2016,<br>36(6):691-703 doi:10.1097/JCP.0000000000000581	Web of Science	Reasons:<br>Not of interest
664.	Targeting the endocannabinoid system with cannabinoid receptor agonists: pharmacological strategies and therapeutic possibilities<br>Pertwee RG.<br>Philos Trans R Soc Lond B Biol Sci. 2012<br>367(1607):3353-63. doi: 10.1098/rstb.2011.0381. Review	Pubmed<br>Scopus<br>Web of Science	Discard<br>Reasons:<br>Not of interest
665.	Techniques and technologies for the bioanalysis of Sativex (R), metabolites and related compounds.<br>Molnar A, Fu SL<br>Bioanalysis 2016<br>8(8): 829-845 doi: 10.4155/bio-2015-0021	Pubmed<br>Scopus<br>Web of Science	Discard<br>Reasons:<br>Not of interest
666.	Tetrahydrocannabinol plus cannabidiol oromucosal spray for multiple sclerosis resistant spasticity on daily practice, new data.<br>Vermersch P, Trojano M<br>Multiple Sclerosis Journal 2016<br>Meeting Abstract: P757 Volume: 22 Pages: 377-377 Supplement: 3	Web of Science	Discard<br>Reasons:<br>Not of interest
667.	Tetrahydrocannabinol/Cannabidiol Oromucosal Spray in Patients With Multiple Sclerosis: A Pilot Study on the Plasma Concentration-Effect Relationship<br>Contin M, Mancinelli L, Perrone A, Sabattini L, Mohamed S, Scandellari C, Foschi M, Vacchiano V, Lugaresi A, Riva R.<br>Clin Neuropharmacol. 2018<br>41(5):171-176. doi: 10.1097/WNF.0000000000000294	Pubmed<br>Scopus<br>Web of Science	Discard<br>Reasons:<br>Not of interest
668.	Tetrahydrocannabinol: Cannabidiol oromucosal spray for multiple sclerosis-related resistant spasticity in daily practice<br>Vermersch, P, Trojano M<br>European Neurology 2016<br>76(5-6):216-226 doi:10.1159/000449413	Scopus<br>Web of Science	Discard<br>Reasons:<br>Not of interest
669.	Tetrahydrocannabinol: cannabidiol oromucosal spray for treating symptoms of multiple sclerosis spasticity: newest evidence<br>Ziemssen T.<br>Neurodegener Dis Manag. 2019<br>9(2s):1-2. doi: 10.2217/nmt-2018-0048.	Pubmed<br>Scopus<br>Web of Science	Discard<br>Reasons:<br>Not of interest
670.	THC and CBD oromucosal spray (Sativex ®) in the management of spasticity associated with multiple sclerosis.<br>Sastre-Garriga,J, Vila C, Clissold S, Montalban, X	Pubmed<br>Scopus<br>Web of Science	Discard<br>Reasons:<br>Not of interest

	Expert Review of Neurotherapeutics 2011<br>11(5): 627-637 doi: 10.1586/ERN.11.47		
671.	Tetrahydrocannabinol and cannabidiol oromucosal spray in resistant multiple sclerosis spasticity: consistency of response across subgroups from the SAVANT randomized clinical trial<br>Meuth SG, Henze T, Essner U, Trompke C, Vila SilvÃ¡n C.<br>Int J Neurosci. 2020<br>1-7. doi: 10.1080/00207454.2020.1730832	Pubmed<br>Scopus<br>Web of Science	Discard<br>Reasons:<br>Not of interest
672.	THC/CBD oromucosal spray in patients with multiple sclerosis overactive bladder: a pilot prospective study<br>Maniscalco GT, Aponte R, Bruzzese D, Guarcello G, Manzo V, Napolitano M, Moreggia O, Chiariello F, Florio C.<br>Neurol Sci. 2018<br>39(1):97-102. doi: 10.1007/s10072-017-3148-6.	Pubmed<br>Scopus<br>Web of Science	Discard<br>Reasons:<br>Not of interest
673.	THC: CBD (Nabiximols) has a beneficial effect on multiple sclerosis related spasticity and delays the need for intrathecal Baclofen.<br>De Trane S, Buchanan K, Keenan L, Valentine C, Liddicut M, Stevenson V, Farrell R<br>JOURNAL OF NEUROLOGY NEUROSURGERY AND PSYCHIATRY 2017<br>Meeting Abstract: PO123 88: A44-A44 DOI: 10.1136/jnnp-2017-ABN.153 Supplement: 1	Web of Science	Discard<br>Reasons:<br>Not of interest
674.	THC: CBD spray and MS spasticity symptoms: Data from latest studies<br>Rekand T<br>European Neurology 2014<br>71(SUPPL.1):4-9-15 doi:10.1159/000357742	Pubmed<br>Scopus<br>Web of Science	Discard<br>Reasons:<br>Not of interest
675.	THC:CBD in Daily Practice: Available Data from UK, Germany and Spain<br>Fernández Ó<br>Eur Neurol. 2016<br>75 Suppl 1:1-3. doi: 10.1159/000444234.	Pubmed<br>Scopus	Discard<br>Reasons:<br>Not of interest
676.	THC:CBD Observational Study Data: Evolution of Resistant MS Spasticity and Associated Symptoms<br>Trojano M.<br>Eur Neurol. 2016<br>75 Suppl 1:4-8. doi: 10.1159/000444235.	Pubmed<br>Scopus	Discard<br>Reasons:<br>Not of interest
677.	THC:CBD oromucosal spray (nabiximols) in the long term treatment of multiple sclerosis spasticity. The MOVE 2 long-term study.<br>Flachenecker P, Zettl U, Henze T<br>Multiple Sclerosis Journal 2013	Web of Science	Discard<br>Reasons:<br>Not of interest

	19(11): 527-527 Supplement: S		
678.	The anti-inflammatory and analgesic effects of formulated full-spectrum cannabis extract in the treatment of neuropathic pain associated with multiple sclerosis<br>Maayah ZH, Takahara S, Ferdaoussi M, Dyck JRB.<br>Inflamm Res. 2020<br>69(6):549-558. doi: 10.1007/s00011-020-01341-1	Pubmed<br>Scopus<br>Web of Science	Discard<br>Reasons:<br>Not of interest
679.	The analgesic potential of cannabinoids<br>Elikottil J, Gupta P, Gupta K<br>Journal of Opioid Management 2009<br>5(6):341-357	Scopus	Discard<br>Reasons:<br>Not of interest
680.	The arguments for and against cannabinoids application in glaucomatous retinopathy.<br>Panah, Y, Manayi A, Nikan M, Vazirian M<br>BIOMEDICINE & PHARMACOTHERAPY 2017<br>86: 620-627 doi: 10.1016/j.biopha.2016.11.106	Web of Science	Discard<br>Reasons:<br>Not of interest
681.	The biology that underpins the therapeutic potential of cannabis-based medicines for the control of spasticity in multiple sclerosis<br>Multiple Sclerosis and Related Disorders 2012<br>1(2):64-75 doi:10.1016/j.msard.2011.11.001	Scopus	Discard<br>Reasons:<br>Not of interest
682.	The Broad Concept of "Spasticity-Plus Syndrome" in Multiple Sclerosis: A Possible New Concept in the Management of Multiple Sclerosis Symptoms<br>Fernàndez Ó, Costa-Frossard L, Martìnez-Ginès M, Montero P, Prieto JM, Ramiò L.<br>Front Neurol. 2020<br>11:152. doi: 10.3389/fneur.2020.00152	Pubmed<br>Scopus<br>Web of Science	Discard<br>Reasons:<br>Not of interest
683.	The cannabinergic system as a target for anti-inflammatory therapies<br>Lu D, Vemuri VK, Duclos Jr. RI, Makriyannis A<br>Current Topics in Medicinal Chemistry 2006<br>6(13):1401-1426 doi:10.1002/mop.21636	Scopus	Discard<br>Reasons:<br>Review
684.	The cannabinoids: an overview. Therapeutic implications in vomiting and nausea after cancer chemotherapy, in appetite promotion, in multiple sclerosis and in neuroprotection<br>Mechoulam R, Hanu L.<br>Pain Res Manag. 2001<br>6(2):67-73. Review doi: 10.1155/2001/183057	Pubmed<br>Scopus	Discard<br>Reasons:<br>Not of interest
685.	The case for medicinal cannabis<br>Irish Journal of Medical Science 2018,<br>187(2):539-540 doi:10.1007/s11845-018-1770-9	Scopus	Discard<br>Reasons:<br>Not of interest
686.	The challenge of clinical application of FM2 cannabis oil produced in Italy for the treatment of neuropathic pain	Scopus	Discard<br>Reasons:

	Pichini S, Pacifici R, Busardò FP, Tagliabracci A, Giorgetti R<br>European Review for Medical and Pharmacological Sciences 2018<br>22(4):863-865		Not of interest
687.	The chromenopyrazole scaffold in the modulation of the endocannabinoid system: A broad therapeutic prospect<br>Morales P, Goya P, Jagerovic N<br>Anales de la Real Academia Nacional de Farmacia 2018<br>84(2):164-184	Scopus	Discard<br>Reasons:<br>Not of interest
688.	The current situation with cannabinoids<br>Kicman AT, King LA<br>Drug Testing and Analysis 2014<br>6(1-2):1-6 doi:10.1002/dta.1597	Scopus	Discard<br>Reasons:<br>Not of interest
689.	The detection of THC, CBD and CBN in the oral fluid of Sativex (R) patients using two on-site screening tests and LC-MS/MS<br>Molnar A, Fu SL, Lewis J, Allsop DJ, Copeland J<br>FORENSIC SCIENCE INTERNATIONAL 2014<br>238: 113-119 doi: 10.1016/j.forsciint.2014.03.004	Pubmed<br>Scopus<br>Web of Science	Discard<br>Reasons:<br>Not of interest
690.	The disease-modifying effects of a Sativex-like combination of phytocannabinoids in mice with experimental autoimmune encephalomyelitis are preferentially due to Δ9-tetrahydrocannabinol acting through CB1 receptors<br>Moreno-Martet M, Feliú A, Espejo-Porras F, Mecha M, Carrillo-Salinas FJ, Fernández-Ruiz J, Guaza C, de Lago E.<br>Mult Scler Relat Disord. 2015<br>4(6):505-11. doi: 10.1016/j.msard.2015.08.001.	Pubmed<br>Scopus<br>Web of Science	Keep
691.	The effect of cannabinoids on the stretch reflex in multiple sclerosis spasticity<br>Marinelli L, Mori L, Canneva S, Colombano F, CurrÃ A, Fattapposta F, Bandini F, Capello E, Abbruzzese G, Trompetto C.<br>Int Clin Psychopharmacol. 2016<br>31(4):232-9. doi: 10.1097/YIC.0000000000000126	Pubmed<br>Scopus<br>Web of Science	Discard<br>Reasons:<br>Not of interest
692.	The effect of cannabis on urge incontinence in patients with multiple sclerosis: a multicentre, randomised placebo-controlled trial (CAMS-LUTS<br>Freeman RM, Adekanmi O, Waterfield MR, Waterfield AE, Wright D, Zajicek J.<br>Int Urogynecol J Pelvic Floor Dysfunct. 2006<br>17(6):636-41. doi: 10.1007/s00192-006-0086-x	Pubmed<br>Scopus	Discard<br>Reasons:<br>Not of interest
693.	The Effectiveness of Cannabinoids in the Management of Chronic Nonmalignant Neuropathic Pain: A Systematic Review.<br>Boychuk DG, Goddard G, Mauro G, Orellana MF	Web of Science	Discard<br>Reasons:<br>Not of interest

	Journal of Oral & Facial Pain and Headache 2015<br>29(1): 7-14 DOI: 10.11607/ofph.1274		
694.	The effects of cannabis, cannabinoids, and their administration routes on pain control efficacy and safety: A systematic review and network meta-analysis<br>Rabgay K, Waranuch N, Chaiyakunapruk N, Sawangjit R, Ingkaninan K, Dilokthornsakul P<br>J Am Pharm Assoc. 2020<br>60(1):225-234.e6. doi: 10.1016/j.japh.2019.07.015	Web of Science	Discard<br>Reasons:<br>Not of interest
695.	The effects of chronic Δ-9-tetrahydrocannabinol (THC) and cannabidiol (CBD) use on cerebral glucose metabolism in multiple sclerosis: A pilot study<br>Workman CD, Kindred JH, Boles Ponto LL, Kamholz J, Rudroff T.<br>Appl Physiol Nutr Metab. 2020<br>45(4):450-452. doi: 10.1139/apnm-2019-0634	Pubmed<br>Scopus<br>Web of Science	Discard<br>Reasons:<br>Not of interest
696.	The effects of clinical interventions on health-related quality of life in multiple sclerosis: A meta-analysis<br>Kuspinar A, Rodriguez AM, Mayo NE<br>Multiple Sclerosis Journal 2012<br>18(12):1686-1704 doi:10.1177/1352458512445201	Scopus	Discard<br>Reasons:<br>Not of interest
697.	The Emerging Role of the Cannabinoid Receptor Family in Peripheral and Neuro-immune Interactions.<br>Haugh O, Penman J, Irving AJ, Campbell VA<br>Current Drug Targets 2016<br>17(16): 1834-1840 doi: 10.2174/1389450117666160112113703	Web of Science	Discard<br>Reasons:<br>Not of interest
698.	The endocannabinoid pathway in Huntington's disease: A comparison with other neurodegenerative diseases<br>Maccarrone M, Battista N, Centonze D<br>Progress in Neurobiology 2007<br>81(5-6):349-379 doi:10.1016/j.pneurobio.2006.11.006	Scopus	Discard<br>Reasons:<br>Not of interest
699.	The endocannabinoid system and its therapeutic exploitation in multiple sclerosis: Clues for other neuroinflammatory diseases<br>Chiurchiù V, van der Stelt M, Centonze D, Maccarrone M<br>Progress in Neurobiology 2018<br>160(82):100 doi:10.1016/j.pneurobio.2017.10.007	Scopus	Discard<br>Reasons:<br>Not of interest
700.	The endocannabinoid system as a potential therapeutic target for pain modulation<br>Ulugöl A<br>Balkan Medical Journal 2014<br>31(2):115-120 doi:10.5152/balkanmedj.2014.13103	Scopus	Discard<br>Reasons:<br>Not of interest

701.	The endocannabinoid system as a target for the treatment of neurodegenerative disease<br>Scotter EL, Abood ME, Glass M<br>British Journal of Pharmacology 2010<br>160(3):480-498 doi:10.1111/j.1476-5381.2010.00735.x	Scopus	Discard<br>Reasons:<br>Not of interest
702.	The endocannabinoid system as an emerging target of pharmacotherapy<br>Pacher P, Bátkai S, Kunos G<br>Pharmacological Reviews 2006<br>58(3):389-462 doi:10.1124/pr.58.3.2	Scopus	Discard<br>Reasons:<br>Not of interest
703.	The endocannabinoid system in the inflammatory and neurodegenerative processes of multiple sclerosis and of amyotrophic lateral sclerosis<br>Rossi S, Bernardi G, Centonze D<br>Experimental Neurology 2010<br>224(1):92-102 doi:10.1016/j.expneurol.2010.03.030	Scopus	Discard<br>Reasons:<br>Not of interest
704.	The Endocannabinoid System: A Dynamic Signalling System at the Crossroads Between Metabolism and Disease<br>Witkamp RF<br>Pharma-Nutrition 2014<br>155-187 doi; 10.1007/978-3-319-06151-1_9	Scopus	Discard<br>Reasons:<br>Not of interest
705.	The endocannabinoid system: A new player in the neurochemical control of vestibular function?<br>Smith PF, Ashton JC, Darlington CL<br>Audiology and Neurotology 2006<br>11(4):207-212 doi:10.1159/000092588	Scopus	Discard<br>Reasons:<br>Not of interest
706.	The endocannabinoid system: A revolving plate in neuro-immune interaction in health and disease<br>Tanasescu R, Gran B, Constantinescu CS<br>Amino Acids 2013<br>45(1):95-112 doi:10.1007/s00726-012-1252-8	Scopus	Discard<br>Reasons:<br>Not of interest
707.	The endocannabinoid system: Overview of an emerging multi-faceted therapeutic target<br>Chanda D, Neumann D, Glatz JFC<br>Prostaglandins Leukotrienes and Essential Fatty Acids 2019<br>140(51):56 doi:10.1016/j.plefa.2018.11.016	Scopus	Discard<br>Reasons:<br>Not of interest
708.	The endogenous cannabinoid system. Therapeutic implications for neurologic and psychiatric disorders [Das endogene cannabinoidsystem. Therapeutische implikationen der cannabinoide bei neurologisch-psychiatrischen erkrankungen]<br>Schneider U, Seifert J, Karst M, Schlimme J, Cimander K, Müller-Vahl KR<br>Nervenarzt 2005	Scopus<br>Web of Science	Discard<br>Reasons:<br>Not of interest

	76(9):1062-1076 doi:10.1007/s00115-005-1888-7		
709.	The entourage effect of the phytocannabinoids<br>Sanchez-Ramos J<br>Annals of Neurology 2015<br>77(6):1083 doi:10.1002/ana.24402	Scopus	Discard<br>Reasons:<br>Not of interest
710.	The Impact of Cannabinoid Receptor 2 Deficiency on Neutrophil Recruitment and Inflammation<br>Hussain,MT, Greaves DR, Iqbal AJ<br>DNA and Cell Biology 2019<br>38(10): 1025-1029 doi: 10.1089/dna.2019.5024	Web of Science	Discard<br>Reasons:<br>Not of interest
711.	The impact of cannabis and cannabinoids for medical conditions on health-related quality of life: A systematic review and meta-analysis<br>Goldenberg M, Reid MW, IsHak WW, Danovitch, I<br>Drug and Alcohol Dependence 2017<br>174:80-90 doi:10.1016/j.drugalcdep.2016.12.030	Scopus<br>Web of Science	Discard<br>Reasons:<br>Not of interest
712.	The impact of drugs for multiple sclerosis on sleep<br>Lanza G, Ferri R, Bella R, Ferini-Strambi L<br>Multiple Sclerosis 2017<br>23(1):5-13 doi:10.1177/1352458516664034	Scopus	Discard<br>Reasons:<br>Not of interest
713.	The influence of physiotherapy intervention on patients with multiple sclerosis-related spasticity treated with nabiximols (THC:CBD oromucosal spray).<br>Grimaldi AE, De Giglio L, Haggiag S, Bianco A, Cortese A, Crisafulli SG, Monteleone F, Marfia G, Prosperini L, Galgani S, Mirabella M, Centonze D, Pozzilli C, Castelli L.<br>PLoS One. 2019<br>14(7):e0219670. doi: 10.1371/journal.pone.0219670.	Pubmed<br>Scopus<br>Web of Science	Discard<br>Reasons:<br>Not of interest
714.	The influence of THC:CBD oromucosal spray on driving ability in patients with multiple sclerosis-related spasticity<br>Celius EG, Vila C.<br>Brain Behav. 2018<br>8(5):e00962. doi: 10.1002/brb3.962.	Pubmed<br>Scopus<br>Web of Science	Discard<br>Reasons:<br>Not of interest
715.	The medical letter®: On drugs and therapeutics<br>[No author name available]<br>Medical Letter on Drugs and Therapeutics 2010<br>52(1330):5-6	Scopus	Discard<br>Reasons:<br>Not of interest
716.	The Medicinal Use of Cannabis and Cannabinoids-An International Cross-Sectional Survey on Administration Forms.<br>Hazekamp A, Ware MA, Muller-Vahl KR, Abrams D, Grotenhermen F (	Web of Science	Discard<br>Reasons:<br>Not of interest

	Journal of Psychoactive Drugs 2013<br>45(3): 199-210 doi: 10.1080/02791072.2013.805976		
717.	The molecular mechanisms that underpin the biological benefits of full-spectrum cannabis extract in the treatment of neuropathic pain and inflammation.<br>Maayah ZH, Takahara S, Ferdaoussi M, Dyck JRB<br>Biochimica et Biophysica acta. Molecular Basis of Disease 2020<br>1866(7):165771 doi: 10.1016/j.bbadis.2020.165771	Web of Science	Discard<br>Reasons:<br>Not of interest
718.	The multiplicity of action of cannabinoids: Implications for treating neurodegeneration<br>Gowran A, Noonan J, Campbell, V.A<br>CNS Neuroscience and Therapeutics 2011<br>17(6):637-644 doi:10.1111/j.1755-5949.2010.00195.x	Scopus	Discard<br>Reasons:<br>Not of interest
719.	The non-psychoactive plant cannabinoid, cannabidiol affects cholesterol metabolism-related genes in microglial cells<br>Rimmerman N, Juknat A, Kozela E, Levy R, Bradshaw HB, Vogel Z.<br>Cell Mol Neurobiol. 2011<br>31(6):921-30. doi: 10.1007/s10571-011-9692-3.	Pubmed<br>Scopus<br>Web of Science	Discard<br>Reasons:<br>Not of interest
720.	The path to the first FDA-approved cannabis-derived treatment and what comes next<br>Rubin R<br>JAMA - Journal of the American Medical Association 2018<br>320(12):1227-1229 doi:10.1001/jama.2018.11914	Scopus	Discard<br>Reasons:<br>Not of interest
721.	The Pharmacologic and Clinical Effects of Medical Cannabis.<br>Borgelt LM, Franson KL, Nussbaum AM, Wang GS<br>PHARMACOTHERAPY 2013<br>33(2): 195-209 doi: 10.1002/phar.1187	Web of Science	Discard<br>Reasons:<br>Not of interest
722.	The pharmacology of cannabinoid receptors and their ligands: An overview<br>Pertwee RG<br>International Journal of Obesity 2006<br>30:S13-S18 doi:10.1038/sj.ijo.0803272	Scopus	Discard<br>Reasons:<br>Not of interest
723.	The profile of immune modulation by cannabidiol (CBD) involves deregulation of nuclear factor of activated T cells (NFAT).<br>Kaplan,BLF, Springs AEB, Kaminski NE<br>Biochemical Pharmacology 2008<br>76 (6) 726-737 doi: 10.1016/j.bcp.2008.06.022	Web of Science	Discard<br>Reasons:<br>Not of interest
724.	The role of cannabinoids to manage pain [El papel de los canabinoides en el manejo del dolor<br>Covarrubias-Gómez A<br>Revista Mexicana de Anestesiologia 2008	Scopus	Discard<br>Reasons:<br>Not of interest

	31(3):191-200		
725.	The role of macrophages in neuroinflammatory and neurodegenerative pathways of alzheimer’s disease, amyotrophic lateral sclerosis, and multiple sclerosis: Pathogenetic cellular effectors and potential therapeutic targets<br>International Journal of Molecular Sciences 2018<br>19(3):831 doi:10.3390/ijms19030831	Scopus	Discard<br>Reasons:<br>Not of interest
726.	The role of medicinal cannabis in clinical therapy: Pharmacists' perspectives<br>Isaac S, Saini B, Chaar BB<br>PLoS ONE 2016<br>11(5):e0155113 doi:10.1371/journal.pone.0155113	Scopus	Discard<br>Reasons:<br>Not of interest
727.	The role of Sativex in robotic rehabilitation in individuals with multiple sclerosis: Rationale, study design, and methodology<br>Russo M, Dattola V, Logiudice AL, Ciurleo R, Sessa E, De Luca R, Bramanti P, Bramanti A, Naro A, Calabrò² RS.<br>Medicine (Baltimore). 2017<br>96(46):e8826. doi: 10.1097/MD.0000000000008826	Pubmed<br>Scopus<br>Web of Science	Discard<br>Reasons:<br>Not of interest
728.	The role of the endocannabinoid system in Alzheimer's disease: facts and hypotheses<br>Bisogno T, Di Marzo V.<br>Curr Pharm Des. 2008<br>14(23):2299-3305. Review doi: 10.2174/138161208785740027	Pubmed<br>Scopus<br>Web of Science	Discard<br>Reasons:<br>Not of interest
729.	The role of the endocannabinoid system in eating disorders: Pharmacological implications<br>Marco EM, Romero-Zerbo SY, Viveros MP, Bermudez-Silva F.J<br>Behavioural Pharmacology 2012<br>23(5-6):526-536 doi:10.1097/FBP.0b013e328356c3c9	Scopus	Discard<br>Reasons:<br>Not of interest
730.	The safety of cannabinoids for the treatment of multiple sclerosis<br>Smith PF<br>Expert Opinion on Drug Safety 2005<br>4(3):443-456 doi:10.1517/14740338.4.3.443	Scopus	Discard<br>Reasons:<br>Not of interest
731.	The spectrum of psychosis in multiple sclerosis: A clinical case series<br>Gilberthorpe TG, O’Connell KE, Carolan A, Silber E, Brex PA, Sibtain NA, David AS<br>Neuropsychiatric Disease and Treatment 2017<br>13:303-318 doi:10.2147/NDT.S116772	Scopus	Discard<br>Reasons:<br>Not of interest
732.	The synthetic cannabinoid WIN 55,212-2 increases COX-2 expression and PGE2 release in murine brain-derived endothelial cells following Theiler's virus infection<br>Mestre L, Correa F, Docagne F, Clemente D, Guaza C<br>Biochemical Pharmacology 2006<br>72(7):869-880 doi:10.1016/j.bcp.2006.06.037	Pubmed<br>Scopus<br>Web of Science	Discard<br>Reasons:<br>Not of interest

733.	The texicology of cannabis and cannabis prohibition<br>Grotenhermen F<br>Chemistry and Biodiversity 2007<br>4(8):1744-1769 doi:10.1002/cbdv.200790151	Scopus	Discard<br>Reasons:<br>Not of interest
734.	The therapeutic potential of the cannabinoids in neuroprotection<br>Grundy RI<br>Expert Opinion on Investigational Drugs 2002<br>11(10):1365-1374 doi:10.1517/13543784.11.10.1365	Scopus	Discard<br>Reasons:<br>Not of interest
735.	The therapeutic use of cannabinoids: Forensic aspects<br>Indorato F, Liberto A, Ledda C, Romano G, Barbera N<br>Forensic Science International 2016<br>265:200-203 doi:10.1016/j.forsciint.2016.03.031	Pubmed<br>Scopus<br>Web of Science	Discard<br>Reasons:<br>Not of interest
736.	The therapeutic value of cannabinoids in MS: Real or imaginary?<br>Killestein J, Polman C<br>Multiple Sclerosis 2004<br>10(4):339-340 doi:10.1191/1352458504ms1064ed	Scopus	Discard<br>Reasons:<br>Not of interest
737.	The treatment of lower urinary tract symptoms in patients with multiple sclerosis: A systematic review<br>Tubaro A, Puccini F, De Nunzio C, Digesu GA, Elneil S, Gobbi C, Khullar, V<br>Current Urology Reports 2012<br>13(5):335-342 doi:10.1007/s11934-012-0266-9	Scopus	Discard<br>Reasons:<br>Not of interest
738.	The use of cannabinoids for sleep: A critical review on clinical trials<br>Kuhathasan N, Dufort A, MacKillop J, Gottschalk R, Minuzzi L, Frey BN.<br>Exp Clin Psychopharmacol. 2019<br>27(4):383-401. doi: 10.1037/pha0000285.	Pubmed<br>Scopus<br>Web of Science	Discard<br>Reasons:<br>Not of interest
739.	The use of cannabinoids in multiple sclerosis<br>Teare L, Zajicek J<br>Expert Opinion on Investigational Drugs 2005<br>14(7):859-869 doi:10.1517/13543784.14.7.859	Pubmed<br>Scopus<br>Web of Science	Discard<br>Reasons:<br>Not of interest
740.	The Use of Cannabis and Cannabinoids in Treating Symptoms of Multiple Sclerosis: a Systematic Review of Reviews<br>Nielsen S, Germanos R, Weier M, Pollard J, Degenhardt L, Hall W, Buckley N, Farrell M.<br>Curr Neurol Neurosci Rep. 2018<br>18(2):8. doi: 10.1007/s11910-018-0814-x. Review	Pubmed<br>Scopus<br>Web of Science	Discard<br>Reasons:<br>Not of interest
741.	The Use of Medical Cannabis with Other Medications: A Review of Safety and Guidelines - An Update [Internet].	Pubmed	Discard<br>Reasons:

	MacDonald E, Adams A.<br>Ottawa (ON): Canadian Agency for Drugs and Technologies in Health 2019 "		Not of interest
742.	The Void in Clinician Counseling of Cannabis Use<br>Calcaterra SL, Cunningham CO, Hopfer CJ.<br>J Gen Intern Med. 2020<br>35:1875–1878 doi:10.1007/s11606-019-05612-4	Pubmed<br>Scopus<br>Web of Science	Discard<br>Reasons:<br>Not of interest
743.	The use of medical-grade cannabis in patients non-responders to Nabiximols<br>Saccà F, Pane C, Carotenuto A, Massarelli M, Lanzillo R, Florio EB, Brescia Morra V.<br>J Neurol Sci. 2016<br>368:349-51. doi: 10.1016/j.jns.2016.07.059.	Pubmed<br>Scopus<br>Web of Science	Discard<br>Reasons:<br>Not of interest
744.	The year's new drugs & biologics<br>Graul AI, Cruces E<br>Drugs of Today 2010,2011<br>47(1):27-51 doi:10.1358/dot.2011.47.1.1587820	Scopus	Discard<br>Reasons:<br>Not of interest
745.	Therapeutic action of cannabidiol in a murine model of Multiple Sclerosis: Protective effects on oligodendrocyte progenitor cultures.<br>Miriam M, Paula I, Leyre M, Miriam H, Katie G, Carmen G<br>JOURNAL OF NEUROIMMUNOLOGY 2010<br>228(1-2) Special Issue: SI Pages: 203-203	Web of Science	Discard<br>Reasons:<br>Not of interest
746.	Therapeutic applications by cannabinoid action [Aplicaciones terapéuticas por acción de los cannabinoides]<br>Plancarte-Sánchez R, Mansilla-Olivares A, De los Reyes-Pacheco VA, Meneses-González F<br>Gaceta Medica de Mexico 2019<br>155(3):307-318 doi:10.24875/GMM.18004928	Pubmed<br>Scopus<br>Web of Science	Discard<br>Reasons:<br>Not of interest
747.	Therapeutic cannabinoids in multiple sclerosis: immunomodulation revisited<br>Constantinescu CS, Gershkovich P.<br>Eur J Neurol. 2018<br>25(7):905-906. doi: 10.1111/ene.13658.	Pubmed<br>Scopus<br>Web of Science	Discard<br>Reasons:<br>Not of interest
748.	Therapeutic impact of orally administered cannabinoid oil extracts in an experimental autoimmune encephalomyelitis animal model of multiple sclerosis<br>Zhou T, Ahmad TK, Alrushaid S, Pozdirca M, Ethans K, Intrater H, Le T, Burczynski F, Kong J, Namaka M.<br>Biochem Biophys Res Commun. 2019<br>516(2):373-380. doi: 10.1016/j.bbrc.2019.06.033.	Pubmed<br>Scopus<br>Web of Science	Keep
749.	Therapeutic potential of cannabinoid medicines<br>Robson PJ	Pubmed<br>Scopus	Discard<br>Reasons:

	Drug Testing and Analysis 2014<br>6(1-2):24-30 doi:10.1002/dta.1529	Web of Science	Not of interest
750.	Therapeutic potential of cannabinoid-based drugs<br>Klein TW, Newton CA<br>Advances in Experimental Medicine and Biology 2007<br>601:395-413 doi:10.1007/978-0-387-72005-0_43	Scopus	Discard<br>Reasons:<br>Not of interest
751.	Therapeutic potential of cannabinoids in CNS disease<br>Croxford JL<br>CNS Drugs 2003<br>17(3):179-202 doi:10.2165/00023210-200317030-00004	Pubmed<br>Scopus<br>Web of Science	Discard<br>Reasons:<br>Not of interest
752.	Therapeutic potential of cannabis in pain medicine<br>Hosking RD, Zajicek JP<br>British Journal of Anaesthesia 2008<br>101(1):59-68 doi:10.1093/bja/aen119	Scopus	Discard<br>Reasons:<br>Not of interest
753.	Therapeutic potential of cannabis-related drugs<br>Alexander SPH<br>Progress in Neuro-Psychopharmacology and Biological Psychiatry 2016<br>64:157-166 doi:10.1016/j.pnpbp.2015.07.001	Scopus	Discard<br>Reasons:<br>Not of interest
754.	Therapeutic potential of CB2 targeting in multiple sclerosis<br>Docagne F, Mestre L, Loría F, Hernangómez M, Correa F, Guaza C<br>Expert Opinion on Therapeutic Targets 2008<br>12(2):185-195 doi:10.1517/14728222.12.2.185	Scopus	Discard<br>Reasons:<br>Not of interest
755.	Therapeutic potential of medicinal marijuana: An educational primer for health care professionals<br>Mouhamed Y, Vishnyakov A, Qorri B, Sambi M, Frank SMS, Nowierski C, Lamba A, Bhatti U, Szewczuk MR<br>Drug, Healthcare and Patient Safety 2018<br>10:45-66 doi:10.2147/DHPS.S158592	Scopus	Discard<br>Reasons:<br>Not of interest
756.	Therapeutic potential of phytocannabinoids and synthetic derivatives affecting human endocannabinoid system<br>Peč J, Riedingerová E, Martin J, Kršková, Z, Dušek, J<br>Ceska a Slovenska Farmacie 2008<br>57(5):195-207	Scopus	Discard<br>Reasons:<br>Not of interest
757.	Therapeutic satisfaction and subjective effects of different strains of pharmaceutical-grade cannabis<br>Brunt TM, van Genugten M, Höner-Snoeken K, van de Velde MJ, Niesink RJ.<br>J Clin Psychopharmacol. 2014	Pubmed<br>Scopus<br>Web of Science	Discard<br>Reasons:<br>Not of interest

	34(3):344-9. doi: 10.1097/JCP.0000000000000129		
758.	Therapeutic use of cannabinoids in neurology [Therapeutischer Einsatz von Cannabinoiden bei neurologischen Erkrankungen]<br>Schwenkreis P, Tegenthoff M<br>Schmerz 2003<br>17(5):367-373 doi:10.1007/s00482-003-0240-z	Scopus	Discard<br>Reasons:<br>Not of interest
759.	Therapeutic use of cannabis derivatives<br>Benyamina A, Reynaud M.<br>Rev Prat. 2014<br>64(2):165-8. French.	Pubmed<br>Scopus	Discard<br>Reasons:<br>Not of interest
760.	Therapeutic uses of cannabinoides [Uso terapéutico de los cannabinoides]<br>Duran Delmàs M, Capellà Hereu D<br>Adicciones 2004<br>16(2):143-152	Scopus	Discard<br>Reasons:<br>Not of interest
761.	Therapeutic utility of cannabinoid receptor type 2 (CB2) selective agonists<br>Han S, Thatte J, Buzard DJ, Jones RM<br>Journal of Medicinal Chemistry 2013<br>56(21):8224-8256 doi:10.1021/jm4005626	Scopus	Discard<br>Reasons:<br>Not of interest
762.	There is evidence for the use of cannabinoids for symptomatic treatment of multiple sclerosis<br>Bazinski H, Jensen HB, Stenager E.<br>Ugeskr Laeger. 2015<br>177(20):956-60. Review. Danish.	Pubmed<br>Scopus	Discard<br>Reasons:<br>Not of interest
763.	Toll-like receptor signalling as a cannabinoid target in Multiple Sclerosis<br>Fitzpatrick J-MK., Downer EJ<br>Neuropharmacology 2017<br>113:618.626 doi:10.1016/j.neuropharm.2016.04.009	Scopus	Discard<br>Reasons:<br>Not of interest
764.	Towards a better understanding of the cannabinoid-related orphan receptors GPR3, GPR6, and GPR12<br>Morales P, Isawi I, Reggio PH<br>Drug Metabolism Reviews 2018<br>50(1):74-93 doi:10.1080/03602532.2018.1428616	Scopus	Discard<br>Reasons:<br>Not of interest
765.	Toxicological properties of Delta (9)-tetrahydrocannabinol and cannabidiol<br>Cerne K<br>Archives of Industrial Hygiene and Toxicology 2020<br>71(1):1-11 doi: 10.2478/aiht-2020-71-3301	Web of Science	Discard<br>Reasons:<br>Not of interest

766.	Traditional marijuana, high-potency cannabis and synthetic cannabinoids: increasing risk for psychosis<br>Murray RM, Quigley H, Quattrone D, Englund A, Di Forti M<br>World Psychiatry 2016<br>15(3):195-204 doi:10.1002/wps.20341	Scopus	Discard<br>Reasons:<br>Not of interest
767.	Transdermal/transmucosal drug delivery: New technologies and outsourcing opportunities<br>Di Filippo P<br>Contract Pharma 2013<br>1-2	Scopus	Discard<br>Reasons:<br>Not of interest
768.	Transparency of funding of patient groups is mandatory but is not enough<br>Colombo C, Mosconi P.<br>BMJ. 2014<br>349:g6301. doi: 10.1136/bmj.g6301. No abstract available.	Pubmed<br>Scopus	Discard<br>Reasons:<br>Not of interest
769.	Treatment failure of intrathecal baclofen and supra-additive effect of nabiximols in multiple sclerosis-related spasticity: a case report<br>Stroet A, Trampe N, Chan A.<br>Ther Adv Neurol Disord. 2013<br>6(3):199-203. doi: 10.1177/1756285613475835	Pubmed<br>Scopus<br>Web of Science	Discard<br>Reasons:<br>Not of interest
770.	Treatment of gastrointestinal disorders: EGS-21<br>Mealy NE, Bayés M<br>Drugs of the Future 2005<br>30(6):608-609	Scopus	Discard<br>Reasons:<br>Not of interest
771.	Treatment of Periodontal Ligament Stem Cells with MOR and CBD Promotes Cell Survival and Neuronal Differentiation via the PI3K/Akt/mTOR Pathway.<br>Cariccio VL, Scionti D, Raffa A, Iori R, Pollastro F, Diomede F, Bramanti P, Trubiani O, Mazzon E<br>Iinternational Journal of Molecular Sciences 2018<br>19(8) Article Number: 2341 DOI: 10.3390/ijms19082341	Web of Science	Discard<br>Reasons:<br>Not of interest
772.	Treatment of spasticity in multiple sclerosis: new perspectives regarding the use of cannabinoids<br>Oreja-Guevara C.<br>Rev Neurol. 2012<br>55(7):421-30. Review. Spanish. doi: 10.33588/rn.5507.2012362	Pubmed<br>Scopus<br>Web of Science	Discard<br>Reasons:<br>Not of interest
773.	Treatment of symptoms in multiple sclerosis<br>Schwendimann RN<br>Neurological Research 2006<br>28(3):306-315 doi:10.1179/016164106X98206	Scopus	Discard<br>Reasons:<br>Not of interest

774.	Treatment with Nabiximols induces short-term and long-term transcriptional changes at peripheral level in patients with multiple sclerosis.<br>Boneschi FM, Ferre L, Sorosina M, Mascia E, Pavan G, Nuara A, Martinelli V, Clarelli F, Esposito F, Comi G<br>Multiple Sclerosis Journal Meeting Abstract: EP1287 Volume: 21 Pages: 672-673 Supplement: 11 Published: SEP 2015	Web of Science	Discard<br>Reasons:<br>Not of interest
775.	Trials a step forward for medicinal cannabis but what comes next?<br>Penington D<br>Australasian Biotechnology 2015<br>25(2):16-17	Scopus	Discard<br>Reasons:<br>Not of interest
776.	U.S. policy responses to calls for the medical use of Cannabis<br>Hall W<br>Yale Journal of Biology and Medicine 2015<br>88(3):257-264	Scopus	Discard<br>Reasons:<br>Not of interest
777.	Upper motor neuron evaluation in multiple sclerosis patients treated with Sativex(Â®).<br>Carotenuto A, Iodice R, Petracca M, Inglese M, Cerillo I, Cocozza S, Saiote C, Brunetti A, Tedeschi E, Manganelli F, Orefice G.<br>Acta Neurol Scand. 2017<br>135(4):442-448. doi: 10.1111/ane.12660	Pubmed<br>Scopus	Discard<br>Reasons:<br>Not of interest
778.	Use of Cannabinoids for Spasticity and Pain Management in MS<br>Chohan H, Greenfield AL, Yadav V, Graves J<br>Current Treatment Options in Neurology 2016<br>18(1):1-14 doi:10.1007/s11940-015-0385-y	Pubmed<br>Scopus<br>Web of Science	Discard<br>Reasons:<br>Not of interest
779.	Using cannabis in oncology: facts and myths<br>Küronya Z, Martin T, Kiss E, Szőnyi M, Bíró K<br>Orv Hetil 2020<br>161(25):1035-1041 doi:10.1556/650.2020.31733	Web of Science	Discard<br>Reasons:<br>Not of interest
780.	Variability of Multiple Sclerosis Spasticity Symptoms in Response to THC:CBD Oromucosal Spray: Tracking Cases through Clinical Scales and Video Recordings<br>Flachenecker P, SaccÃ F, Vila C.","Case Rep Neurol. 2018<br>10(2):169-176. doi: 10.1159/000490376.	Pubmed<br>Scopus<br>Web of Science	Discard<br>Reasons:<br>Not of interest
781.	Voiding function and dysfunction, bladder physiology and pharmacology, and female urology<br>Brady CM, DasGupta R, Dalton C, Wiseman OJ, Berkley KJ, Fowler CJ, Wein AJ, Ostaszkiewicz J, Johnston L, Roe B, Wallace SA, Roe B, Williams K, Palmer M, Seif C, Jünemann KP, Braun PM., Goldberg RP, Tchetgen MB, Sand PK, Koduri S, Rackley R, Appell R, Culligan PJ	Scopus	Discard<br>Reasons:<br>Not of interest

	Journal of Urology 2005<br>173(49):1262-1266 doi:10.1097/01.ju.0000155014.15023.57		
782.	Walking improvements with nabiximols in patients with multiple sclerosis<br>Coghe G, Pau M, Corona F, Frau J, Lorefice L, Fenu G, Spinicci G, Mamusa E, Musu L, Massole S, Massa R, Marrosu MG, Cocco E.<br>J Neurol. 2015<br>262(11):2472-7. doi: 10.1007/s00415-015-7866-5.	Pubmed<br>Scopus<br>Web of Science	Discard<br>Reasons:<br>Not of interest
783.	What is medicinal cannabis?<br>Glass M, Ashton JC<br>New Zealand Medical Journal 2019<br>132(1494):49-56	Scopus	Discard<br>Reasons:<br>Not of interest
784.	What is new in multiple sclerosis spasticity research? Poster session highlights<br>Ghezzi A.<br>Neurodegener Dis Manag.<br>6(6s):45-47.<br>Neurodegener Dis Manag. 2016 doi 10.2217/nmt-2016-0056:	Pubmed<br>Scopus<br>Web of Science	Discard<br>Reasons:<br>Not of interest
785.	What is new in symptomatic MS treatment: Part 2-gait disorder and spasticity.<br>Henze T, Feneberg W, Flachenecker P, Seidel D, Albrecht H, Starck M, Meuth SG<br>NERVENARZT 2017<br>88(12): 1428-1434 DOI: 10.1007/s00115-017-0439-3	Web of Science	Discard<br>Reasons:<br>Not of interest
786.	What place for ▼ cannabis extract in MS?<br>[No authors listed]<br>Drug and Therapeutics Bulletin 2012<br>50(12):141-144 doi:10.1136/dtb.2012.11.0150	Pubmed<br>Scopus	Discard<br>Reasons:<br>Not of interest
787.	What regulation for therapeutic cannabis? [Quelle réglementation pour le cannabis thérapeutique?]<br>Michon F<br>Soins 2019<br>64:833 doi:10.1016/j.soin.2019.01.017	Scopus	Discard<br>Reasons:<br>Not of interest
788.	What to make of cannabis and cognition in MS: In search of clarity amidst the haze<br>Feinstein A, Banwell E, Pavisian B<br>Multiple Sclerosis 2015<br>21(14):1755-1760 doi:10.1177/1352458515607652	Scopus	Discard<br>Reasons:<br>Not of interest
789.	What's new in multiple sclerosis spasticity research? Poster session highlights<br>Linker R<br>Neurodegener Dis Manag.<br>7(6s):51-53. doi: 10.2217/nmt-2017-0043	Pubmed<br>Scopus<br>Web of Science	Discard<br>Reasons:<br>Not of interest

790.	Who benefits most from THC:CBD spray? Learning from clinical experience<br>Koehler J.<br>Eur Neurol. 2014<br>71 Suppl 1:10-5. doi: 10.1159/000357743.	Pubmed<br>Scopus<br>Web of Science	Discard<br>Reasons:<br>Not of interest
791.	Who will support independent patient groups?<br>Batt S.<br>BMJ. 2014<br>349:g6306. doi: 10.1136/bmj.g6306	Pubmed<br>Scopus	Discard<br>Reasons:<br>Not of interest.
792.	Whole plant cannabis extracts in the treatment of spasticity in multiple sclerosis: a systematic review<br>Lakhan SE, Rowland M.<br>BMC Neurol. 2009<br>9:59. doi: 10.1186/1471-2377-9-59	Pubmed<br>Scopus<br>Web of Science	Discard<br>Reasons:<br>Not of interest
793.	Wider use of cannabinoids likely soon?<br>Jack DB<br>Drug News and Perspectives 1997<br>10(7):440-442	Scopus	Discard<br>Reasons:<br>Not of interest
794.	Will medicinal cannabinoids prove to be useful clinically?<br>Smith PF<br>Current Drug Therapy 2007<br>2(2):143-150 doi:10.2174/157488507780619059	Scopus	Discard<br>Reasons:<br>Not of interest
795.	Willingness to pay for a treatment for pain in multiple sclerosis<br>Iskedjian M, Desjardins O, Piwko C, Bereza B, Jaszewski B, Einarson TR.<br>Pharmacoeconomics. 2009<br>27(2):149-58. doi: 10.2165/00019053-200927020-00005	Pubmed<br>Scopus	Discard<br>Reasons:<br>Not of interest
796.	Yield and cannabinoids contents in different cannabis (Cannabis sativa L.) genotypes for medical use<br>Janatová A, Fraňková A, Tlustoš P, Hamouz K, Božik M, Klouček P<br>Industrial Crops and Products 2018<br>112:363-367 doi:10.1016/j.indcrop.2017.12.006	Sopus<br>Web of Science	Discard<br>Reasons:<br>Not of interest

Note: “Not of interest” means that the reference is not an original research which does not deal with CBD and immune system/neuroinflammation in multiple sclerosis
